# Neoadjuvant pyrotinib and trastuzumab in HER2-positive breast cancer with no early response (NeoPaTHer): efficacy, safety and biomarker analysis of a prospective, multicentre, response-adapted study

**DOI:** 10.1038/s41392-025-02138-6

**Published:** 2025-01-29

**Authors:** Fei Wang, Yongjiu Wang, Bin Xiong, Zhenlin Yang, Jingfen Wang, Yumin Yao, Lixiang Yu, Qinye Fu, Liang Li, Qiang Zhang, Chao Zheng, Shuya Huang, Liyuan Liu, Chun Liu, Huaibo Sun, Beibei Mao, Dong-Xu Liu, Zhigang Yu

**Affiliations:** 1 Breast Center, The Second Hospital of Shandong University, Jinan, China; 2 Shandong Key Laboratory of Cancer Digital Medicine, Jinan, China; 3 Shandong Provincial Engineering Laboratory of Translational Research on Prevention and Treatment of Breast Disease, Jinan, China; 4 Institute of Translational Medicine of Breast Disease Prevention and Treatment, Shandong University, Jinan, China; 5 Department of Breast Surgery, Affiliated Hospital of Jining Medical University, Jining, China; 6 Department of Breast Surgery, Binzhou Medical University Hospital, Binzhou, China; 7 Department of Breast Surgery, Linyi Cancer Hospital, Linyi, China; 8 Department of Breast Surgery, Liaocheng People’s Hospital, Liaocheng, China; 9 Genecast Biotechnology Co.Ltd., Wuxi, China

**Keywords:** Breast cancer, Drug regulation

## Abstract

The potential benefits of pyrotinib for patients with trastuzumab-insensitive, HER2-positive early-stage breast cancer remain unclear. This prospective, multicentre, response-adapted study evaluated the efficacy and safety of adding pyrotinib to the neoadjuvant treatment of HER2-positive breast cancer patients with a poor response to initial docetaxel plus carboplatin and trastuzumab (TCbH). Early response was assessed using magnetic resonance imaging (MRI) after two cycles of treatment. Patients showing poor response, as defined by RECIST 1.1, could opt to receive additional pyrotinib or continue standard therapy. The primary endpoint was the total pathological complete response (t*p*CR; ypT0/isN0) rate. Of the 129 patients enroled, 62 (48.1%) were identified as MRI-responders (cohort A), 26 non-responders continued with four more cycles of TCbH (cohort B), and 41 non-responders received additional pyrotinib (cohort C). The t*p*CR rate was 30.6% (95% CI: 20.6–43.0%) in cohort A, 15.4% (95% CI: 6.2–33.5%) in cohort B, and 29.3% (95% CI: 17.6–44.5%) in cohort C. Multivariable logistic regression analyses demonstrated comparable odds of achieving t*p*CR between cohorts A and C (odds ratio = 1.04, 95% CI: 0.40–2.70). No new adverse events were observed with the addition of pyrotinib. Patients with co-mutations of *TP53* and *PIK3CA* exhibited lower rates of early partial response compared to those without or with a single gene mutation (36.0% vs. 60.0%, *P* = 0.08). These findings suggest that adding pyrotinib may benefit patients who do not respond to neoadjuvant trastuzumab plus chemotherapy. Further investigation is warranted to identify biomarkers predicting patients’ benefit from the addition of pyrotinib.

## Introduction

Human epidermal growth factor receptor 2 (HER2)-positive breast cancer, which accounts for ~15% to 25% of all breast cancers,^1^ is recognised as a distinct and aggressive subtype. It is characterised by the overexpression of HER2, a transmembrane receptor tyrosine kinase that promotes cancer cell proliferation and survival. Patients with HER2-positive breast cancer face a significantly higher risk of recurrence and mortality compared to those with other breast cancer subtypes, making it a particularly challenging disease to treat. However, the landscape of HER2-positive breast cancer treatment has undergone remarkable transformation in the last two decades, primarily due to the introduction of HER2-targeted therapies. In particular, the advent of trastuzumab has fundamentally changed the trajectory of HER2-positive breast cancer from a highly fatal disease to one with more manageable and less dire outcomes.^2,3^

Neoadjuvant therapy, which is administered before surgery, allows for the assessment of treatment efficacy by monitoring tumour response, and it has become a standard of care for patients with early-stage HER2-positive breast cancer.^4,5^ Over the years, there has been increasing interest in optimising the use of neoadjuvant HER2-targeted therapies by exploring new agents and combining them with trastuzumab to further enhance patient outcomes. The combination of trastuzumab and pertuzumab, for instance, has demonstrated notable increases in total pathological complete response (t*p*CR) rates, rising to 39.3% compared to trastuzumab monotherapy.^6,7^ As the landscape of HER2-targeted therapies continues to evolve, newer agents such as pyrotinib, a small-molecule irreversible pan-ErbB TKI,^8,9^ have emerged and shown promising results in preclinical and clinical studies. The combination of trastuzumab with pyrotinib has demonstrated a similar therapeutic response in patients with HER2-positive breast cancer, with the PHEDRA study reporting a t*p*CR rate of 41.0%.^10^ However, despite these promising results, there remains a lack of direct comparative data regarding the efficacy of combining trastuzumab with pertuzumab versus trastuzumab with pyrotinib or other TKIs in the neoadjuvant setting. This absence of comparative data is particularly challenging given the variability in baseline tumour characteristics across different clinical trials, which complicates the determination of patients who may benefit more from trastuzumab plus pyrotinib compared to trastuzumab plus pertuzumab. In the metastatic setting, the efficacy of pyrotinib has been well-documented. Data from the PHILA study, for example, demonstrated that pyrotinib significantly improved progression-free survival in patients with metastatic HER2-positive breast cancer who had previously been treated with adjuvant trastuzumab.^11^ Additionally, preclinical models, including those conducted by our research team, have shown that pyrotinib can enhance the effectiveness of trastuzumab in trastuzumab-resistant cell lines. Notably, pertuzumab did not exhibit the same synergistic effect,^12–14^ suggesting that TKIs such as pyrotinib may be crucial for overcoming resistance in patients who fail to respond to trastuzumab. This observation points to the potential role of pyrotinib in the neoadjuvant setting, particularly for patients with primary resistance or limited sensitivity to trastuzumab. However, clinical evidence regarding the use of neoadjuvant pyrotinib in this population is still lacking, and further studies are needed to determine its role in this context.

Neoadjuvant treatment not only aims to reduce tumour size but also offers a unique opportunity to evaluate drug sensitivity, which is vital for determining the most appropriate subsequent adjuvant treatment strategy.^15^ For example, patients with HER2-positive breast cancer who do not achieve a pathological complete response (*p*CR) after neoadjuvant therapy may benefit from an escalation of their adjuvant treatment regimen, such as T-DM1.^4^ Furthermore, numerous studies have underscored the importance of early clinical responses to neoadjuvant therapy, as these responses can be predictive of long-term outcomes.^16^ Radiological assessments, particularly those using magnetic resonance imaging (MRI), have shown that early response can guide treatment decisions and improve treatment outcomes.^17,18^ Despite the benefits of neoadjuvant therapy, there remains significant debate regarding the optimal management of patients who exhibit poor early responses. Traditionally, patients with inadequate responses have been transitioned to early definitive surgery, but this approach can potentially hinder the evaluation of drug sensitivity, thereby complicating subsequent treatment decisions.^19^ Emerging evidence, however, suggests that modifying the treatment regimen based on early response assessments may yield better survival outcomes. The phase 3 GeparTrio trial, for example, demonstrated that response-guided neoadjuvant therapy significantly improved long-term survival in breast cancer patients.^20^ Similarly, the PHERGain trial found that patients who responded early to dual HER2 blockade, as assessed by ^18^F-FDG-PET imaging, had a higher rate of *p*CR, further supporting the potential benefits of a response-adapted strategy that could deliver an individualised chemotherapy de-escalation.^21^ This growing body of evidence raises an important question: should patients with suboptimal early responses continue on single-agent trastuzumab, or should they escalate to dual HER2 blockade? This area remains underexplored, and further research is needed to determine the most effective approach for managing patients with poor early responses to neoadjuvant therapy.

To address some of these critical gaps, we proposed the prospective, response-adapted NeoPaTHer trial. In this study, we evaluated the efficacy and safety of adding pyrotinib to a neoadjuvant regimen of trastuzumab plus chemotherapy in patients with HER2-positive breast cancer who did not exhibit an early response to trastuzumab-based therapy alone. Our findings indicate that the addition of pyrotinib significantly improves the t*p*CR rate in these patients, offering valuable insights into personalised treatment strategies for this challenging patient population. Furthermore, biomarker analyses from the NeoPaTHer trial may provide a better understanding of which patients are most likely to benefit from the addition of pyrotinib, paving the way for more tailored and effective treatment strategies in HER2-positive breast cancer.

## Results

### Patients

**Fig. 1:**
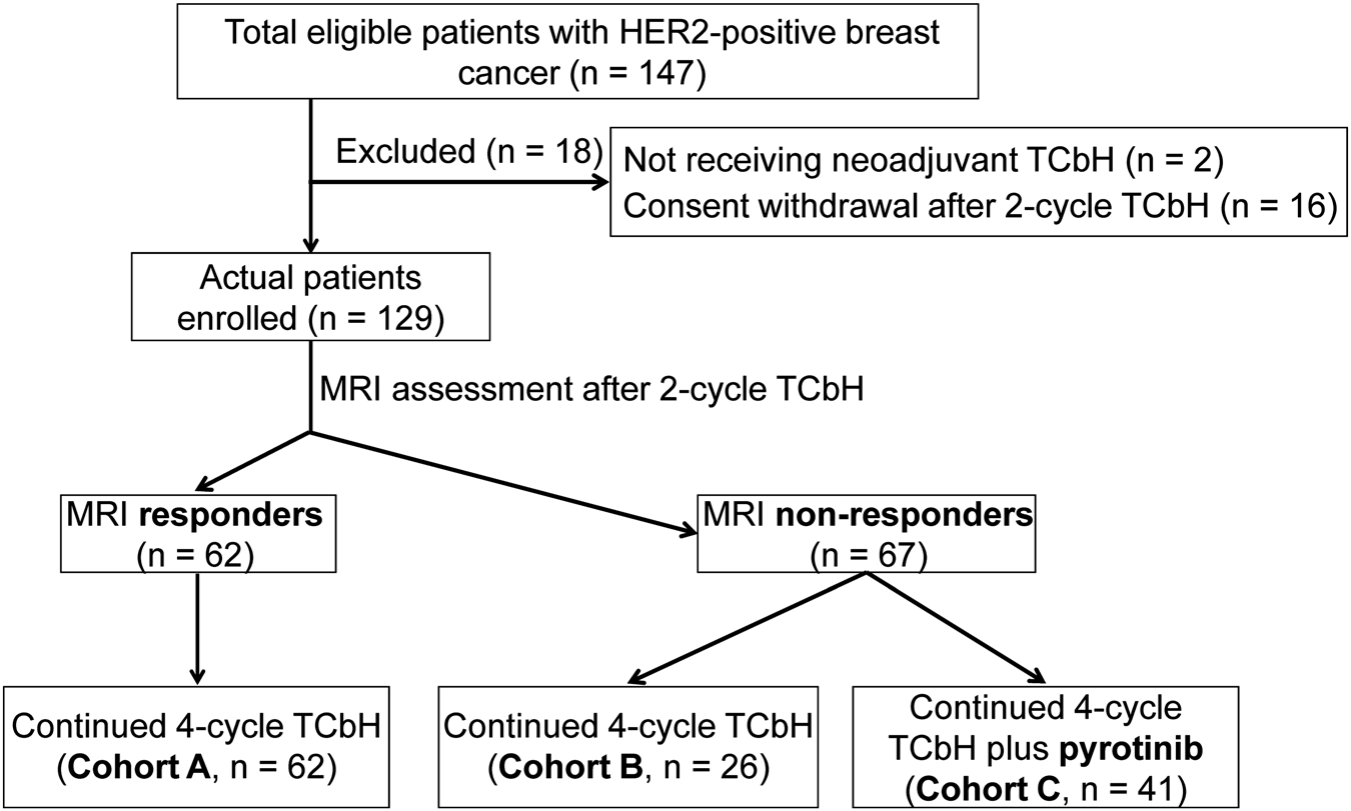
CONSORT diagram. Patients with HER2-positive breast cancer who received neoadjuvant therapy across five hospitals were assessed for eligibility. Breast magnetic resonance imaging (*MRI*) was performed at baseline, after every two cycles of neoadjuvant therapy, and prior to surgery. Early response was evaluated after the first two cycles of docetaxel plus carboplatin and trastuzumab (*TCbH*) treatment according to the Response Evaluation Criteria in Solid Tumours (RECIST) version 1.1. Responders continued with an additional four cycles of TCbH treatment (cohort A). Non-responders were further divided into cohorts B and C based on their preferences. Cohort B received four more cycles of neoadjuvant TCbH treatment, and cohort C received TCbH combined with oral pyrotinib for four cycles

Between October 2020 and March 2022, a total of 129 patients from five hospitals were enroled in this study. After two cycles of neoadjuvant treatment, 62 patients (48.1%) were identified as MRI-responders and assigned to cohort A. Among the remaining 67 MRI non-responders, 26 patients continued with four additional cycles of docetaxel plus carboplatin and trastuzumab (TCbH; cohort B), while 41 patients received four cycles of TCbH in combination with pyrotinib (cohort C; Fig. 1).

As shown in Table 1, baseline demographic and disease characteristics were generally comparable across the study cohorts. A large proportion of patients enroled had stage II disease (62.6% in cohort A, 61.5% in cohort B, and 73.2% in cohort C, *P* = 0.57), hormone receptor (HR)-positive cancer (67.7% in cohort A, 76.9% in cohort B, and 61.0% in cohort C, *P* = 0.40), and Ki67 levels ≥20% (82.3% in cohort A, 73.1% in cohort B, and 78.0% in cohort C, *P* = 0.62). Additionally, HER2 protein 3+, as confirmed by immunohistochemistry (IHC), was prevalent across the study cohorts (88.7% in cohort A, 96.2% in cohort B, and 80.5% in cohort C, *P* = 0.16).

**Table 1:** Baseline characteristics of patients in the three cohorts stratified by MRI response and treatment regimens

Characteristics	Patients (*N* = 129)			*P*
	Cohort A (*n* = 62)	Cohort B (*n* = 26)	Cohort C (*n* = 41)	
Median age of years (range)	50.0 (43.2–54.8)	52.5 (47.2–59.8)	50.0 (44.0–54.0)	0.15
Menopausal status, *n* (%)				0.21
Postmenopausal	30 (48.4)	17 (65.4)	18 (43.9)	
Premenopausal	32 (51.6)	9 (34.6)	23 (56.1)	
Clinical T stage, *n* (%)				0.94
T1	6 (9.7)	2 (7.7)	4 (9.8)	
T2	50 (80.6)	19 (73.1)	31 (75.6)	
T3	4 (6.5)	3 (11.5)	4 (9.8)	
T4	2 (3.2)	2 (7.7)	2 (4.9)	
Lymph node status, *n* (%)				0.27
N0	22 (35.5)	12 (46.2)	19 (46.3)	
N1	32 (51.6)	8 (30.8)	13 (31.7)	
N2	7 (11.3)	5 (19.2)	9 (22.0)	
N3	1 (1.6)	1 (3.8)	0 (0.0)	
TNM status, *n* (%)				0.57
II	45 (72.6)	16 (61.5)	30 (73.2)	
III	17 (27.4)	10 (38.5)	11 (26.8)	
HR status, *n* (%)				0.40
ER^+^ and/or PR^+^	42 (67.7)	20 (76.9)	25 (61.0)	
ER^-^ and PR^-^	20 (32.3)	6 (23.1)	16 (39.0)	
HER2 status, *n* (%)				0.16
HER2 (IHC score 3+)	55 (88.7)	25 (96.2)	33 (80.5)	
HER2 (IHC score 2+)	7 (11.3)	1 (3.8)	8 (19.5)	
Ki67 level, *n* (%)				0.62
<20%	11 (17.7)	7 (26.9)	9 (22.0)	
≥20%	51 (82.3)	19 (73.1)	32 (78.0)	

*HR* hormone receptor, *ER*^*+*^ estrogen receptor-positive, *ER*^*-*^ estrogen receptor-negative, *PR*^*+*^ progesterone receptor-positive, *PR*^*-*^ progesterone receptor-negative, *HER2* human epidermal growth factor receptor 2, *IHC* immunohistochemistry

### Efficacy analysis

**Fig. 2:**
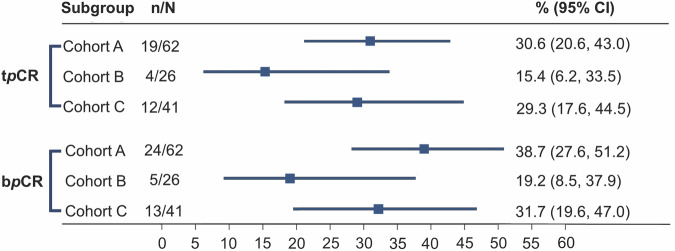
Pathological complete response (*p*CR) in the intention-to-treat population according to magnetic resonance imaging (*MRI*) response and treatment assignment. Total *p*CR (t*p*CR, ypT0/isN0) and breast *p*CR (b*p*CR, ypT0/is) in the intention-to-treat population (*N* = 129), stratified by MRI response and treatment: Cohort A (MRI responders treated with docetaxel, carboplatin, and trastuzumab), Cohort B (MRI non-responders treated with docetaxel, carboplatin, and trastuzumab), and Cohort C (MRI non-responders treated with docetaxel, carboplatin, trastuzumab, and pyrotinib). The number (n) of patients who achieved *p*CR in a subgroup (N) are presented. The *p*CR rates were calculated with a 95% confidence interval (*CI*)

All patients completed the planned six cycles of neoadjuvant therapy without experiencing disease progression, and subsequently proceeded to surgery. Among the MRI responders in cohort A, a total of 19 out of 62 patients achieved t*p*CR, resulting in a rate of 30.6% (95% confidence interval [CI]: 20.6–43.0%). Among the MRI non-responders, cohort C exhibited a higher t*p*CR rate of 29.3% (12 out of 41 patients, 95% CI: 17.6–44.5%) compared to cohort B, which had a rate of 15.4% (4 out of 26 patients, 95% CI: 6.2–33.5%). Similar trends were observed in exploratory analyses for the breast pathological complete response (b*p*CR) rate. The highest b*p*CR rate was found in cohort A at 38.7% (95% CI: 27.6–51.2%), followed by cohort C at 31.7% (95% CI: 19.6–47.0%), whereas cohort B had the lowest rate of 19.2% (95% CI: 8.5–37.9%; Fig. 2).

Subgroup analyses also suggested the differences in both t*p*CR and b*p*CR rates across the study cohorts, regardless of HR status, HER2 IHC status, TNM stage, Ki67 level and menopausal status. The rates were generally lower in patients with HR-positive breast cancer compared to those with HR-negative disease, regardless of treatment group (Supplementary Table 1).

As shown in Supplementary Fig. 1, after adjustment for age, menopausal status, HR status, HER2 IHC status, and Ki67 level, there was a non-significant trend indicating that cohort B exhibited a reduced likelihood of achieving t*p*CR (odds ratio [OR] = 0.40, 95% CI: 0.11 to 1.42) compared to cohort A, while cohort C showed a comparable likelihood of achieving t*p*CR (OR = 1.04, 95% CI: 0.40 to 2.70). Similar association patterns were observed regarding b*p*CR in the exploratory analyses (Supplementary Fig. 1). However, multivariable logistic regression analyses showed that no significant associations between clinicopathological variables and early response to neoadjuvant TCbH treatment (Supplementary Fig. 2).

With a median follow-up of 3.2 years, survival data remain immature, with only five disease-free survival (DFS) events recorded (three events in cohort A, one in cohort B, and one in cohort C): three visceral metastases, one axillary nodal recurrence, and one chest recurrence. No deaths were observed.

### Safety analysis

Adverse events (AEs) reported in all 129 patients are summarised in Table 2. In cohort C, which received pyrotinib in addition to TCbH treatment, the most common AEs were diarrhoea (24 out of 41 patients, 58.5%), erythropenia (19 patients, 46.3%), and anaemia (18 patients, 43.9%). Grade 3–4 AEs were rare, and no fatalities were reported. Furthermore, no patients had a left ventricular ejection fraction (LVEF) below 50%, nor did any experience a reduction greater than 10% from baseline prior to surgery. Among the 41 patients in cohort C, 13 (31.7%) required a pyrotinib dose reduction due to diarrhoea; however, no patients required dose reductions for docetaxel, carboplatin, trastuzumab, or pyrotinib for other reasons. These findings are consistent with previous clinical trials involving pyrotinib, where diarrhoea was the most frequently reported AE leading to dose reductions of pyrotinib.^10,22^

**Table 2:** Adverse events recorded in the NeoPaTHer trial^a^

Adverse events	Cohort A (*n* = 62)		Cohort B (*n* = 26)		Cohort C (*n* = 41)		*P*
	Grade 1–2	Grade 3	Grade 1–2	Grade 3	Grade 1–2	Grade 3	
Haematological toxicity, *n* (%)							
Anaemia	36 (58.1)	2 (3.2)	10 (38.5)	0	18 (43.9)	1 (2.4)	0.13
Leukopenia	29 (46.8)	15 (24.2)	14 (53.9)	2 (7.7)	10 (24.4)	2 (4.9)	<0.01
Neutropenia	24 (38.7)	12 (19.4)	12 (46.2)	3 (11.5)	4 (9.8)	2 (4.9)	<0.01
Thrombocytopenia	12 (19.4)	0	4 (15.4)	1 (3.9)	4 (9.8)	1 (2.4)	0.45
Erythropenia	43 (69.4)	3 (4.8)	12 (46.2)	0	19 (46.3)	0	0.01
Non-haematological toxicity, *n* (%)							
Diarrhoea	5 (8.1)	0	1 (3.9)	0	24 (58.5)	3 (7.3)	<0.01
Vomiting	12 (19.4)	0	5 (19.2)	0	8 (19.5)	0	0.16
Nausea	14 (22.6)	0	9 (34.6)	0	9 (22.0)	0	0.43
Fatigue	2 (3.2)	0	4 (15.4)	0	4 (9.8)	0	0.13
Weight loss	0	0	0	0	3 (7.3)	0	0.04^#^
Alanine aminotransferase increased	5 (8.1)	0	7 (26.9)	0	2 (4.9)	0	0.01
Aspartate aminotransferase increased	1 (1.6)	0	2 (7.69)	0	1 (2.4)	0	0.16^#^
Creatinine increased	4 (6. 5)	0	0	0	5 (12.2)	0	0.16
Hyponatremia	5 (8.1)	1 (1.6)	1 (3.9)	0	3 (7.3)	0	0.77
Hypokalaemia	4 (6. 5)	0	1 (3.9)	0	3 (7.3)	0	0.84
Hypomagnesaemia	5 (8.1)	0	1 (3.9)	0	3 (7.3)	0	0.77
Hyperglycaemia	4 (6. 5)	0	2 (7.7)	0	1 (2.4)	0	0.58
Hypertriglyceridemia	7 (11.3)	0	4 (15.4)	0	1 (2.4)	0	0.16
Hypocalcaemia	1 (1.6)	0	0	0	7 (17.1)	0	<0.01^#^
γ-glutamyl transferase increased	9 (14.5)	0	2 (7.7)	0	6 (14.6)	1 (2.4)	0.64
Hypoalbuminemia	31 (50.0)	0	1 (3.9)	0	10 (24.4)	0	<0.01
Hyperphosphatemia	3 (4.8)	0	0	0	2 (4.9)	0	0.71^#^

^a^Adverse events of any grade occurring in more than 10% of patients, as well as grade ≥3 adverse events, were reported for all enroled patients. No grade 4 adverse events were recorded in any of the three cohorts

*P*-values represent the comparisons of grade 1–2 adverse events among the cohorts, calculated using the chi-square test (or Fisher’s exact test, as indicated by the hash symbol #). Due to the low frequency of grade ≥3 adverse events, statistical analysis of differences in these events across the cohorts was not conducted

### Biomarker analysis

Pre-treatment samples of tumour tissue and peripheral blood were collected from 70 patients, 37 of whom achieved a partial response after the initial 2 cycles of TCbH treatment, while 33 did not respond. Twelve patients, eight of whom had stable disease (non-responders), withdrew from the study after the initial treatment. Among the 58 patients included in the biomarker analysis, 33 patients were from cohort A, three from cohort B, and 22 patients from cohort C. Detailed characteristics of these patients are provided in Supplementary Tables 2 and 3.

**Fig. 3:**
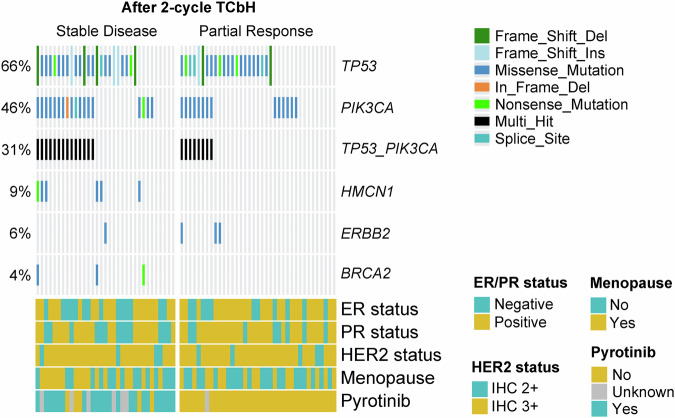
Landscape of somatic single nucleotide variants in 70 patients with HER2-positive breast cancer, stratified by early response assessed using MRI after two cycles of neoadjuvant treatment. DNA sequencing was conducted using a custom-designed gene panel encompassing 769 frequently mutated genes in solid tumours. Gene mutations were analysed in relation to early response, which was assessed by MRI after the first two cycles of TCbH treatment according to the Response Evaluation Criteria in Solid Tumours (RECIST) version 1.1. Patients with a partial response (*n* = 37) were defined as those showing at least a 30% reduction in the sum of the longest diameters of all target lesions from baseline; patients not meeting this criterion were classified as having stable disease (*n* = 33). The types of gene mutations identified in each patient are annotated, with the mutation rate for each type presented on the left. Additionally, the status of pathological markers, including estrogen receptor (*ER*), progesterone receptor (*PR*), and HER2, as determined by immunohistochemistry (*IHC*), as well as menopause status and pyrotinib treatment, are also shown

The most frequent somatic mutations observed before treatment were *TP53* (46/70 patients; 66%), *PIK3CA* (32/70; 46%), *HMCN1* (6/70; 9%), *ERBB2* (4/70; 6%), and *BRCA2* (3/70; 4%). Co-mutations of *TP53* and *PIK3CA* were present in 22 (31%) patients (Fig. 3). Non-responders showed higher frequencies of *HMCN1* mutations (18.0% vs. 0.0%, *P* = 0.008) and *BRCA2* mutations (9.0% vs. 0.0%, *P* = 0.10) compared to early responders (data not shown). Although the frequencies of *TP53* and *PI3KCA* mutations were similar between responders and non-responders, patients with co-mutations of *TP53* and *PIK3CA* were less likely to achieve an early partial response compared to those with single-gene mutations or no mutations (36.0% vs. 60.0%, *P* = 0.08) (Supplementary Fig. 3a). However, no significant difference in the t*p*CR rate was observed between patients with and without co-mutations (Supplementary Fig. 3b).

Previous research has demonstrated that genetic mutational characteristics, such as tumour mutation burden (TMB) and genetic heterogeneity, serve as predictive biomarkers for cancer outcomes across various cancer types.^23,24^ However, in our study, no significant differences were observed in TMB (*P* = 0.19) or mutant allele tumour heterogeneity (MATH) scores (*P* = 0.09) between early responders and non-responders (Supplementary Fig. 4a, b). Nevertheless, patients with t*p*CR exhibited significantly higher TMB compared to those without t*p*CR (*P* = 0.01, Supplementary Fig. 4c).

Pathway analyses revealed that the frequency of mutations in the RTK-RAS pathway was significantly higher in the t*p*CR group compared to the non-t*p*CR group (56.0% vs. 21.0%, *P* = 0.02, data not shown). Multivariable logistic regression analysis, adjusted for MRI early response, TMB, RTK-RAS pathway mutation, and DNA damage response pathway mutation, revealed that TMB (OR = 5.38, 95% CI: 1.24–23.36, *P* = 0.03) and RTK-RAS pathway mutation (OR = 3.24, 95% CI: 0.82–12.77, *P* = 0.09) were associated with t*p*CR (Supplementary Table 4).

**Fig. 4:**
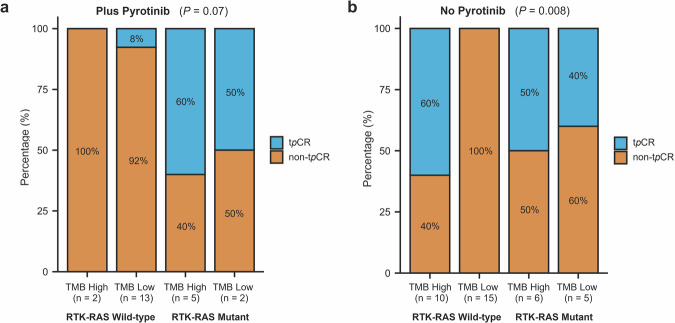
Total pathological complete response (t*p*CR) rates in HER2-positive breast cancer patients, stratified by tumour mutation burden (TMB) levels, RTK-RAS pathway mutation status, and pyrotinib treatment. The t*p*CR and non-t*p*CR rates were calculated for patients who completed six cycles of neoadjuvant treatment with TCbH plus pyrotinib in cohort C (**a**) and those who received TCbH alone (*no pyrotinib*) in cohorts A and B (**b**). Stratification was based on the combined status of TMB levels (*high* or *low*) and RTK-RAS pathway mutations (*wild-type* or *mutant*). *P* values for each comparison are presented

Stratified analyses based on the combined status of TMB and RTK-RAS pathway mutations revealed that patient responses were associated with both TMB levels and the presence of mutations in the RTK-RAS pathway (Supplementary Fig. 5). Patients with RTK-RAS mutations had a remarkable high t*p*CR rate, ranging from 40% to 60%, irrespective of TMB levels or the addition of pyrotinib (Fig. 4). In contrast, patients without RTK-RAS mutations and low TMB exhibited a significantly lower t*p*CR rate, with 0% (0/15) in those receiving trastuzumab and chemotherapy alone, compared to 8% (1/13) in those receiving additional pyrotinib to trastuzumab and chemotherapy (Fig. 4).

## Discussion

In this prospective, multicentre, response-adapted study of HER2-positive breast cancer, we observed that patients who were non-responders to trastuzumab-based neoadjuvant therapy, as determined by breast MRI after two cycles of treatment, had a significantly lower t*p*CR rate compared to those who showed early response. Interestingly, the addition of pyrotinib to trastuzumab increased the t*p*CR rate in non-responders, bringing it in line with that of the early responders, while maintaining good tolerability. This finding offers a potential response-guided strategy for identifying patients insensitive to trastuzumab plus chemotherapy who may benefit from the addition of pyrotinib to trastuzumab as dual HER2 blockade in the neoadjuvant setting. In addition, exploratory biomarker analyses indicated that co-mutations of *TP53* and *PIK3CA* might be negatively associated with early response to initial trastuzumab plus chemotherapy. This insight underscores the importance of response-guided selection in optimising HER2-blocking regimens at an early stage.

Over the past three decades, substantial advancements have been made in the treatment of HER2-positive breast cancer. We are now in an era of individualised treatment based on molecular classification and tumour burden.^4^ Despite numerous studies investigating de-escalation strategies for women with small, node-negative HER2-positive breast cancer,^21,25^ dual HER2 blockade remains the cornerstone of neoadjuvant treatment for this population.^26–28^ Clinical trials have demonstrated that dual HER2 blockade with trastuzumab in combination with either pertuzumab or pyrotinib result in comparable rates of *p*CR.^6,7,10^ However, there is currently a lack of robust evidence on the optimal methodology for administering personalised dual anti-HER2 blockade regimens, such as trastuzumab plus pertuzumab or trastuzumab plus pyrotinib, on individual patient basis.

It has been suggested that assessing early response, such as changes in Ki67 level or radiographic response after 2 or 4 cycles of neoadjuvant treatment, can predict *p*CR following treatment.^29,30^ The PHERGain trial was a strategy-based study designed to investigate the potential omission of chemotherapy while maintaining a backbone of dual HER2 blockade in patients with HER2-positive breast cancer.^21^ Data from this trial showed that patients exhibiting an early response (at cycle 2) to TCbH plus pertuzumab, as assessed by ^18^F-FDG-PET, had significantly higher *p*CR rates compared to non-responders (65.6% vs. 10.0%). However, the study did not evaluate whether altering the treatment regimen based on early response status could further enhance the *p*CR rates.

Similarly, the GeparTrio trial^31^ utilised a response-guided design, in which early responders were identified by ultrasound after 2 cycles of chemotherapy. This trial found that early responders experienced only a modest 2.5% increase in the *p*CR rates, while non-responders had very low *p*CR rates regardless of whether the treatment was switched (6% vs. 5.3%). However, exploratory analyses revealed that both DFS and overall survival were significantly longer in patients receiving response-guided chemotherapy compared to those receiving conventional chemotherapy, emphasising the long-term benefit of an early-response-guided strategy.^20^

Based on these observations, we designed the NeoPaTHer study with the hypothesis that adding pyrotinib to trastuzumab would improve the *p*CR rate in non-responders. As anticipated, our findings showed that early responders, assessed using breast MRI, had a higher *p*CR rate than non-responders. Notably, the addition of pyrotinib resulted in nearly a twofold increase in the *p*CR rate among non-responders. This trend was consistent regardless of HR status, HER2 IHC status, and Ki67 level. However, non-responders with HR-negative cancer or high expression levels of HER2 or Ki67 were more likely to benefit from the addition of pyrotinib, aligning with previous reports.^10^ This indicates that patients with more advanced cancer may be better candidates for a response-guided strategy. Our findings are further supported by in vivo animal studies, which showed that pyrotinib inhibited tumour growth and HER2 downstream pathways more effectively than pertuzumab in HER2-positive breast cancer cells with primary resistance to trastuzumab.^12^ Nevertheless, additional clinical trials are needed to validate these results.

Several previous studies have evaluated the combination of pyrotinib and trastuzumab in the neoadjuvant setting for HER2-postive breast cancer.^32,33^ In the NeoATP study by Yin and colleagues, the combination of neoadjuvant pyrotinib and trastuzumab with weekly paclitaxel-cisplatin resulted in a remarkable *p*CR rate of 69.8%.^32^ Similarly, Xuhong’s study reported a *p*CR rate of 73.7%.^33^ However, both studies employed a single-arm design with a relatively small sample size. In contrast, larger prospective clinical trials, including the NeoSphere (dual HER2 blockade with trastuzumab plus pertuzumab),^6^ NeoALTTO (trastuzumab plus lapatinib),^34^ and PHEDRA (trastuzumab plus pyrotinib),^10^ reported *p*CR rates around 40.0%. In the present study, approximately 30% of patients receiving the combination of pyrotinib and trastuzumab achieved t*p*CR, which was somewhat lower than the rates reported in these previous studies. Importantly, our study focused specifically on a subgroup of patients who were resistant to trastuzumab therapy. This finding underscores the potential complementary role of TKIs in treating trastuzumab-insensitive breast cancer. In vivo studies have showed that TKIs, rather than pertuzumab, may enhance the anticancer activity of trastuzumab in trastuzumab-resistant cell lines.^35^ Future research is needed to explore the underlying mechanisms driving this interaction. Nevertheless, our study provides direct evidence that the addition of pyrotinib may serve as a viable alternative to early definitive surgery for patients who do not respond adequately to trastuzumab.

While our study supports the feasibility of a response-guided approach for HER2-positive breast cancer in the neoadjuvant setting, we were unable to identify specific clinicopathological factors that could reliably predict which patients would benefit from the addition of pyrotinib to trastuzumab. Notably, the predictive value of radiographic response assessed by MRI for t*p*CR in our study fell short of expectation. The t*p*CR rate among early responders in cohort A was only 30.6%, which was lower than the rates reported in the NOAH trial. Similarly, recently published findings from the TRAIN-3 study echoed this observation.^36^ In this single-arm phase 2 study, while breast MRI was effective in identifying early complete responders among patients with HR-negative tumours, it failed to do so for patients with HR-positive, HER2-positive breast cancer. These findings underscore the need for further research to develop simple, reliable, and clinically applicable tools for the assessment of early treatment response.

Interestingly, we found that mutations in *PI3KCA* and *TP53* were associated with poor response to neoadjuvant TCbH treatment, although they did not correlate with t*p*CR rates. This aligns with several previous studies that have shown an association of *PI3KCA* mutations with a lower *p*CR rate in patients with HER2-positive breast cancer who received neoadjuvant trastuzumab.^37–40^ Another noteworthy finding from our biomarker analysis is that the combination of TMB levels and mutational status in the RTK-RAS pathway may assist to identify patients who may benefit from the response-guided strategy. Patients with RTK-RAS mutations, who exhibited initial trastuzumab insensitivity, responded positively to the addition of pyrotinib, indicating that this group may potentially benefit from pyrotinib in combination with trastuzumab. Conversely, patients without RTK-RAS mutations and with low TMB, were expected to display resistance to both trastuzumab and pyrotinib. Although some studies have suggested that RAS mutations are associated with reduced efficacy of trastuzumab in HER2-positive colorectal or gastric cancer,^41^ there is currently limited research on the role of RAS pathway mutations in anti-HER2 response among breast cancer patients. In the future, validation studies are warranted.

One of the key strengths of our study is the unique response-adapted design. To the best of our knowledge, this is the first prospective, response-guided study to evaluate the efficacy of pyrotinib in patients who are insensitive to trastuzumab plus chemotherapy in the neoadjuvant setting. This may facilitate the selection of patients who are more suitable for neoadjuvant therapy with trastuzumab plus pyrotinib. However, we acknowledge that our study has limitations. Firstly, we did not employ a randomised design for cohorts B and C. Instead, patients were allocated based on their preferences following face-to-face discussions. Despite this, baseline characteristics were generally comparable across the cohorts, and we applied logistic regression analyses to adjust for potential confounding factors. These steps helped minimise potential bias associated with the non-randomised design. Although randomisation would have been the optimal approach to control for both known and unknown biases, the results remain reliable due to these careful adjustments. Secondly, we did not establish a distinct cohort of non-responders who would have received additional pertuzumab instead of pyrotinib. As a result, direct evidence comparing the efficacy of trastuzumab plus pyrotinib versus trastuzumab plus pertuzumab in non-responders within this prospective clinical trial is not available. Although our previous in vivo studies have demonstrated that HER2-positive breast cancer cells resistant to trastuzumab are also resistant to pertuzumab, additional clinical data are needed to confirm this observation. Thirdly, our study was designed in 2019, prior to the widespread adoption of neoadjuvant dual HER2 blockade with trastuzumab and pertuzumab in China. Consequently, pertuzumab was not employed as the initial treatment in our study cohort. Therefore, it is possible that our findings do not fully reflect the most current standard of care. However, given the similar mechanisms of action between trastuzumab and pertuzumab,^42^ it seems reasonable to propose that this response-guided strategy could also be applicable to patients receiving trastuzumab and pertuzumab as their initial treatment. Nevertheless, prospective studies are needed to validate this hypothesis.

In conclusion, our study proposed a potential response-guided strategy to identify patients with HER2-positive breast cancer who are insensitive to initial neoadjuvant therapy with trastuzumab plus chemotherapy. Our findings suggest that adding pyrotinib to trastuzumab as dual HER2 blockade may improve the t*p*CR rate in this patient population. This response-guided approach warrants further clinical validation. Additionally, identifying potential pre-treatment biomarkers will be crucial for optimising patient selection.

## Methods

### Study design

The NeoPaTHer trial was a prospective, multicentre, response-adapted study conducted at five hospitals in China, and registered with ClinicalTrials.gov (identifier: NCT03847818). The study protocol received approval from the Institutional Review Board of the Second Hospital of Shandong University (approval no.: KYLL-2020(KJ)P-0145). This research was performed in accordance with the Declaration of Helsinki and Good Clinical Practice guidelines. Written informed consent was obtained from all participating patients through face-to-face discussions prior to the initiation of neoadjuvant therapy. During these discussions, participants received detailed information about the study’s rationale, objectives, procedures, potential risks, and benefits. They were also informed of their right to withdraw consent at any point during the study, particularly following the early response evaluation.

### Participants

Female patients aged 18 to 75 years with histologically confirmed T2-3N0-3M0 primary invasive HER2-positive breast cancer were eligible for this study. TNM staging was evaluated according to the 8^th^ edition of the American Joint Committee on Cancer (AJCC) Cancer Staging Manual. HER2 status was assessed locally and considered positive if the IHC staining score was 3+, or if fluorescent in situ hybridization (FISH) or bright-field HER2 dual in situ hybridization (DISH) tests yielded positive results.^43^ Both HR-positive and HR-negative breast cancers were included in this study. HR status, including estrogen receptor (ER) and progesterone receptor (PR), was categorised as positive when at least 1% of tumour cells exhibited positive staining on IHC. Other key inclusion criteria included an Eastern Cooperative Oncology Group (ECOG) performance status of 0 or 1, and a baseline LVEF of ≥55%, as measured by echocardiography.

Key exclusion criteria included the presence of metastatic disease, a prior history of or concurrent malignant neoplasms, except for curatively treated basal and squamous cell carcinoma of the skin and carcinoma in situ of the cervix. Inflammatory breast cancer was also excluded due to the challenges in assessing therapeutic remission based on a single radial MRI measurement. Additionally, patients with clinically significant cardiovascular conditions, such as a known history of uncontrolled or symptomatic angina, significant arrhythmias, congestive heart failure, transmural myocardial infarction, or uncontrolled hypertension (≥180/110 mmHg), were excluded. Those who were unable or unwilling to swallow tablets were also excluded from this study.

### Procedures

All patients initially received 2 cycles of neoadjuvant TCbH treatment. Docetaxel was administered at a dose of 75 mg/m², while carboplatin was given at a dose of an area under the concentration-time curve (AUC) of 5 mg/mL per min. Trastuzumab was provided at an initial loading dose of 8 mg/kg, followed by maintenance doses of 6 mg/kg. All agents were administered intravenously on day 1 of each 21-day cycle.

Breast MRI was performed on all patients at screening (prior to enrolment), after every two cycles of neoadjuvant therapy, and before surgery. Early response was assessed by MRI after the first two cycles of TCbH treatment according to the Response Evaluation Criteria in Solid Tumours (RECIST) version 1.1.^44^ Patients were classified as MRI responders if all target lesions showed at least a 30% reduction from baseline in the sum of the longest diameters (i.e., complete or partial response); all others were deemed non-responders. Responders proceeded with an additional 4 cycles of neoadjuvant TCbH treatment (cohort A). Non-responders were further divided into cohorts B and C based on their preferences after a second round of face-to-face discussion. These discussions, conducted according to a standardised protocol, assessed early responses and outlined the potential benefits and risks of each treatment option. To minimise selection bias, all investigators underwent comprehensive training before the study’s initiation. Cohort B received another 4 cycles of neoadjuvant TCbH treatment. Cohort C received TCbH plus oral pyrotinib (400 mg once daily) for 4 cycles, with loperamide (two 2-mg tablets, taken three times a day) administered as a primary prophylactic measure against potential diarrhoea.

All patients continued the treatment as per protocol unless there was disease progression, unacceptable toxicity, withdrawal of consent, or a decision by the investigator to discontinue treatment. Surgery was performed 2 to 6 weeks after the last dose of study treatment, with adjuvant therapy administered according to local guidelines or institutional standards. Echocardiography was conducted prior to enrolment and every three months thereafter. AEs were graded using the Common Terminology Criteria for Adverse Events version 5.0 at each cycle.

### Outcomes

The primary endpoint was the proportion of patients achieving t*p*CR, defined as the absence of invasive cancer in both the breast and axilla (ypT0/isN0), as determined by a local pathologist after surgery. The Miller-Payne system was used to assess the pathological response to neoadjuvant therapy, with grade 5 indicating b*p*CR. The secondary endpoint was DFS, defined as the time from definitive surgery to recurrence, metastasis, or death from any cause, whichever occurred first. Patients without DFS events were censored at their last disease assessment.

### Biomarker analysis

Using genomic DNA extracted from tumour tissues and peripheral blood, we conducted DNA sequencing with a custom-designed gene panel encompassing 769 frequently mutated genes in solid tumours. We evaluated single gene mutations, pathway mutations, TMB, and MATH scores in relation to t*p*CR and early response following two cycles of neoadjuvant treatment. Details of the analyses are provided in the Supplementary Materials and Methods.

### Statistical analysis

This study was designed to investigate whether pyrotinib could enhance the t*p*CR rate in patients exhibiting primary resistance or insensitivity to trastuzumab. Given the lack of published data on t*p*CR rates in patients without an early response to trastuzumab, we estimated t*p*CR rates of 12% for cohort B and 35% for cohort C based on real-world observations. Using an estimation design with a 1:2 enrolment ratio, and a significance level of *α* = 0.05 (one-sided) and *β* = 0.20, we determined the required sample sizes for cohorts B and C to be 32 and 63 patients, respectively. Following the routine adoption of initial dual blockade with both trastuzumab and pertuzumab in China since late 2021, significant differences in t*p*CR rates between cohorts B and C led to early termination of enrolment before the targets were reached. No formal hypothesis testing or statistical comparisons of the t*p*CR rates between cohorts was conducted.

Efficacy was assessed in the intention-to-treatment population, which included all enroled patients. Safety was assessed in the safety analysis set, comprising all patients who received at least one dose of the study drug. Descriptive characteristics of the study groups were compared using the chi-square test for categorical variables and the analysis of variance (ANOVA) for continuous variables. The primary endpoint was evaluated for each cohort, with the 95% CI calculated using the Wilson procedure without correction for continuity.^45^ In order to minimise the potential bias arising from the non-randomised design, the t*p*CR rate was compared across cohorts using a multivariable logistic regression model with the Wald test. This model adjusted for several factors, including age, pretreatment T and N stages, HR status, menopausal status, HER2 protein expression (as determined by IHC, specifically IHC score 2+ and IHC score 3+), and Ki67 level (categorised with a 20% cutoff). Due to the relatively small sample sizes in cohorts B and C, a sensitivity analysis using matching methods was not performed. Subgroup analyses were further conducted by HER2 status (IHC score 2+ vs. IHC score 3+), baseline TNM stage (II vs. III), HR status (negative vs. positive), Ki67 level (≤20% vs. >20%), and menopausal status (premenopausal vs. postmenopausal). Multivariable logistic regression model was employed to determine potential clinicopathological variables predicting early response to neoadjuvant TCbH treatment.

All statistical tests were two-sided, with the significance level set at *P* < 0.05, and *P* values presented here were nominal. All statistical analyses were performed using R software (version 4.2.0, R Foundation, Vienna, Austria).

## Supplementary information

Supplementary Materials for Neopather trial

Protocol of the Neopather trial

## Data Availability

The raw sequencing data reported in this paper have been deposited in the Genome Sequence Archive (GSA) at the National Genomics Data Center, China National Center for Bioinformation/Beijing Institute of Genomics, Chinese Academy of Sciences (*GSA: HRA009982*) and are publicly accessible at https://ngdc.cncb.ac.cn/gsa. In accordance with the Regulation of the People’s Republic of China on the Administration of Human Genetic Resources, access to the Human Genetic Resource Data requires an application to the Data Access Committee for research purposes. Users must submit a data access request, which will be approved upon meeting the necessary criteria. The data will then be made available upon reasonable request, subject to the terms of consent and data use limitations for the subjects. For further inquiries, please contact the corresponding author.
