# Supplementary material for: Neoadjuvant pyrotinib and trastuzumab in HER2-positive breast cancer with no early response (NeoPaTHer): efficacy, safety and biomarker analysis of a prospective, multicentre, response-adapted study
Source: Signal Transduct Target Ther. 2025 Jan 29;10:45. doi: 10.1038/s41392-025-02138-6 (PMC11775149; doi:10.1038/s41392-025-02138-6)
Supplement: Supplementary file 1 — Supplementary Materials for Neopather trial [file 41392_2025_2138_MOESM1_ESM.docx]

Supplementary Materials for

Neoadjuvant pyrotinib and trastuzumab in HER2-positive breast cancer with no early response (NeoPaTHer): efficacy, safety and biomarker analysis of a prospective, multicentre, response-adapted study

Fei Wang, Yongjiu Wang, Bin Xiong, Zhenlin Yang, Jingfen Wang, Yumin Yao, Lixiang Yu, Qinye Fu, Liang Li, Qiang Zhang, Chao Zheng, Shuya Huang, Liyuan Liu, Chun Liu, Huaibo Sun, Beibei Mao, Dong-Xu Liu and Zhigang Yu

Correspondence to: yuzhigang@sdu.edu.cn

**This PDF file includes:**

Materials and Methods

Figs. 1 to 5

Tables 1 to 4

References

**Materials and Methods**

**DNA extraction**

Genomic DNA from tumour tissue was extracted from formalin-fixed, paraffin-embedded (FFPE) samples using a MagPure FFPE DNA Kit B (Magen). Genomic DNA from peripheral blood was extracted using a TGuide S32 Magnetic Blood Genomic DNA Kit (Tiangen) and served as a matched control. The concentration of extracted DNA was measured using an Equalbit 1X dsDNA HS Assay Kit (Vazyme), and the quality was assessed with an Agilent 2100 BioAnalyzer (Agilent).

**Library preparation**

Peripheral blood genomic DNA was fragmented into approximately 200 base pairs (bp) fragments using an enzymatic method (5×FEA Enzyme Mix; Qiagen). After end repair and A-tailing, T-adaptors were ligated on both ends using TIANSeq DNA Ligase (Qiagen), and final libraries were obtained via PCR amplification using the KAPA Library Amplification Primer Mix (KAPA Biosystems). FFPE genomic DNA was sheared into 150- to 200-bp fragments using the Covaris M220 Focused-ultrasonicator™ Instrument (Covaris) according to the recommended settings. FFPE library construction was performed using a KAPA Hyper Prep Kit (Roche) following the manufacturer's instructions. All libraries were quantified using an Equalbit 1X dsDNA HS Assay Kit (Vazyme), and library sizes were determined using an Agilent 2100 Bioanalyzer (Agilent).

**Targeted-region capture and DNA sequencing**

Targeted region capture was performed using an xGen Hybridization and Wash kit (IDT, USA, ID:1080584). The cancer gene panel, designed in collaboration with Genecast Biotechnology Co., Ltd., included 769 genes, covering frequently mutated genes in solid tumours. Hybridization and washing were performed according to the manufacturer’s protocol. Captured libraries were sequenced on an Illumina NovaSeq 6000 platform, producing paired-end reads of 150 bp.

Somatic single nucleotide variants (SNVs) and indel mutations were identified in FFPE tumour samples, with matched peripheral blood samples as controls. Paired-end sequencing was performed on an Illumina Novaseq 6000 platform. Sequencing reads containing adaptor sequences and low-quality data were removed using Trimmomatic (version 0.36). High-quality paired-end reads were aligned to the human reference genome (hg19) using the BWA-MEM (bwa-0.7.17) aligner in default mode. SNV calling was performed with VarDict (version 1.5.1), and variants were annotated using ANNOVAR. Variants were excluded based on the following criteria: (a) sequencing coverage < 120X; (b) silent mutations in non-reference alleles; (c) support reads <5; (d) support SNV forward reads <2 or reverse reads < 2; (e) allele frequency ≥0.02 in the Exome Aggregation Consortium (ExAC) or the Genome Aggregation Database (gnomAD); and (f) allele frequency <0.02 in tumour samples.

**Tumour mutational burden (TMB)**

TMB (mutations/Mb) was calculated using the algorithm previously described.^1^ Nonsynonymous somatic mutations at exonic and splicing regions were quantified to determine the TMB value, with variant frequencies ≥5%. The total number of mutations was divided by the size of the coding region in the targeted panel to calculate the TMB per Mb.

**Copy number instability (CNI)**

GC content and the length of the target region were corrected using proprietary algorithms for each region. The read counts were transformed into log_2_ ratios and converted into z-scores based on Gaussian transformations compared to a normal control group (n=30). Target regions with z-scores greater than the 95^th^ percentile and an absolute standard deviation twice that of the normal control group were retained. The remained z-scores were summed to obtain the CNI score.^2^

**Copy-number analysis**

Copy number variants (CNVs) were identified using 30 normal blood samples as control to calculate gene specificity scores (GCS). A joint statistical significance test was performed on the GCS and the absolute value of the copy number to determine CNV. A copy number value >4 was classified as a CNV gain, while a value <1 was classified as a CNV loss. Copy number alteration (CNA) burden was estimated based on the total number of genes with copy number gains and losses.

**Mutant allele tumour heterogeneity (MATH) score**

The variant allele frequency (VAF) was calculated as the ratio of alternate allele observations to the total read depth at each position. The mutant allele tumour heterogeneity (MATH) score^3^ was then applied to all somatic variants with a VAF ranging from 0.02 to 1, using the following formula:

$$MATH=100\times\frac{median absolute deviation (MAD)}{median of the VAF}$$

## Statistical analysis

Statistical analyses were performed using R software (version 4.0.4, R Foundation, Vienna, Austria). Fisher’s exact test was used to evaluate statistical heterogeneity. Continuous variables were compared using the Wilcoxon test. All reported *P*-values were two-tailed, and *P*-values < 0.05 were considered statistically significant.


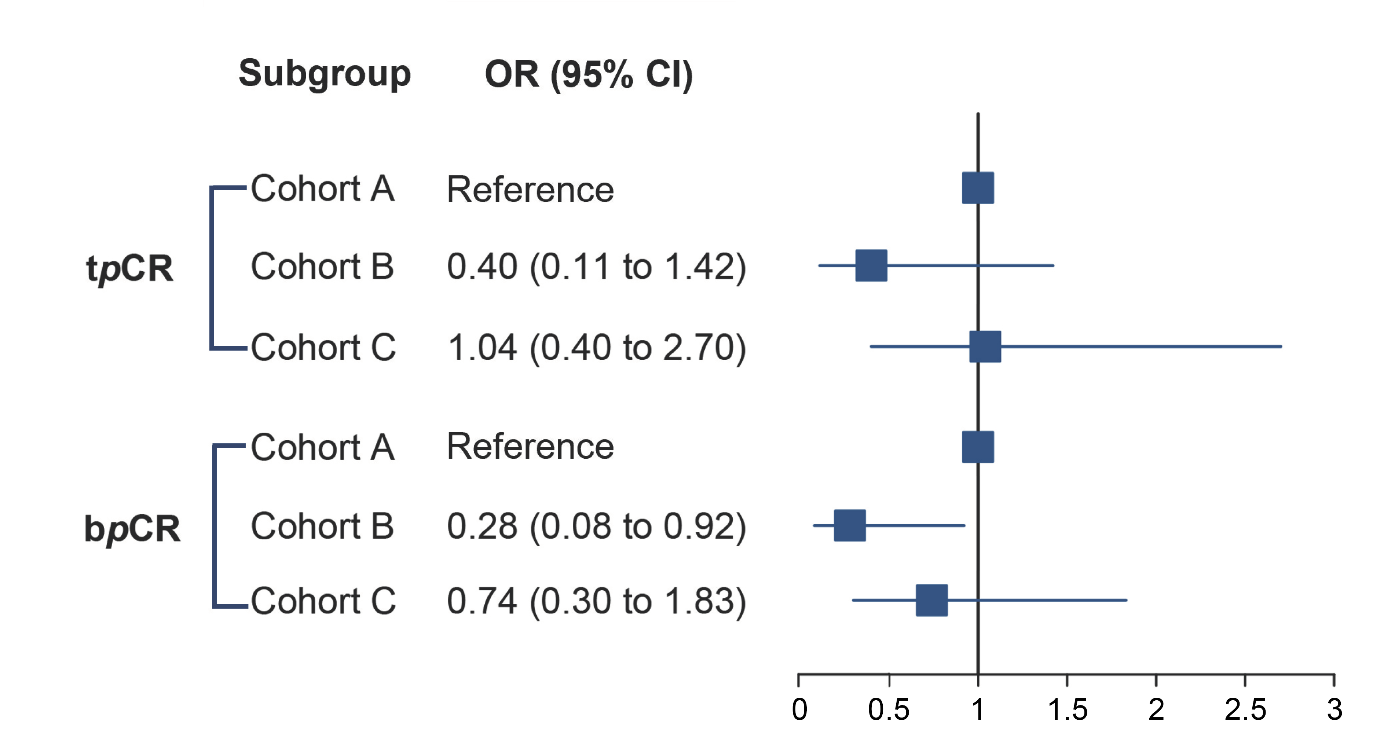


**Fig. 1.** Multivariable-adjusted odds ratios (ORs) of treatment assignments in association with pathological complete response (*p*CR) in the intention-to-treat population. A logistic regression model was used to calculate the ORs and 95% confidence intervals (CI) for treatment cohorts A-C, which were stratified based on MRI response and the use of pyrotinib, in relation to *p*CR, adjusting for age (continuous variable), HR status, menopausal status, HER2 protein expression (IHC score 2+ and IHC score 3+), and Ki67 (categorized with a cutoff value of 20%). Cohort A were MRI responders treated with docetaxel, carboplatin, and trastuzumab, Cohort B were MRI non-responders treated with docetaxel, carboplatin, and trastuzumab), and Cohort C were MRI non-responders treated with docetaxel, carboplatin, trastuzumab, and pyrotinib.

Abbreviations: *MRI* magnetic resonance imaging, *tpCR* total *p*CR, *bpCR* breast *p*CR, *HR* hormone receptor, *HER2* human epidermal growth factor receptor 2, *IHC* immunohistochemistry

**
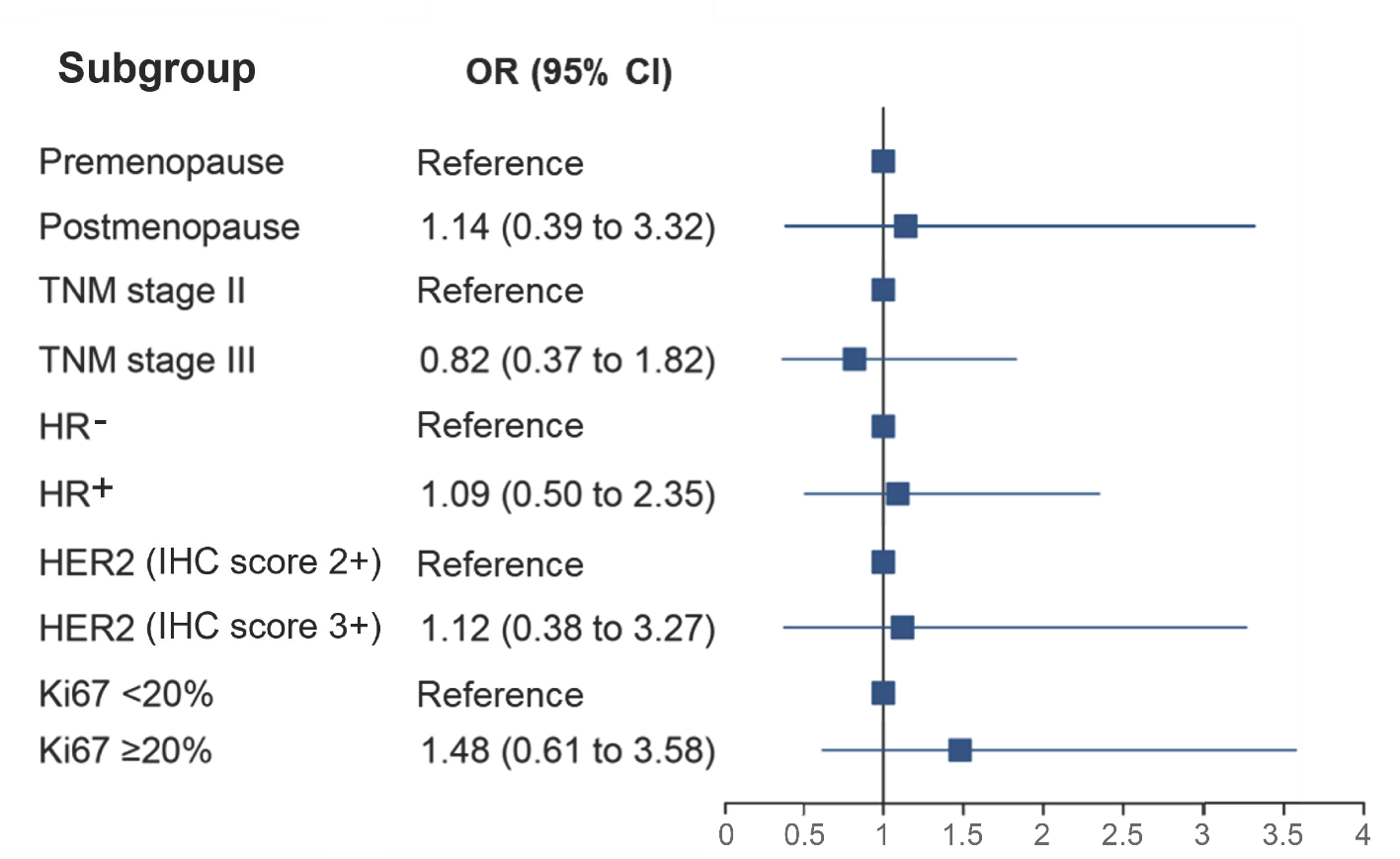
**

**Fig. 2.** Association of clinicopathological characteristics with early response to neoadjuvant TCbH Treatment. Multivariable logistic regression models were used to estimate odds ratios (OR) and 95% confidence intervals (CI) for early response (defined as partial response after two cycles of TCbH treatment) in relation to menopausal status, TNM stage, HR status, HER2 status, and Ki67 levels, with additional adjustment for age (continuous variable).

Abbreviations: *HR^+^* hormone receptor-positive, *HR^-^* hormone receptor-negative, *HER2* human epidermal growth factor receptor 2, *IHC* immunohistochemistry

**
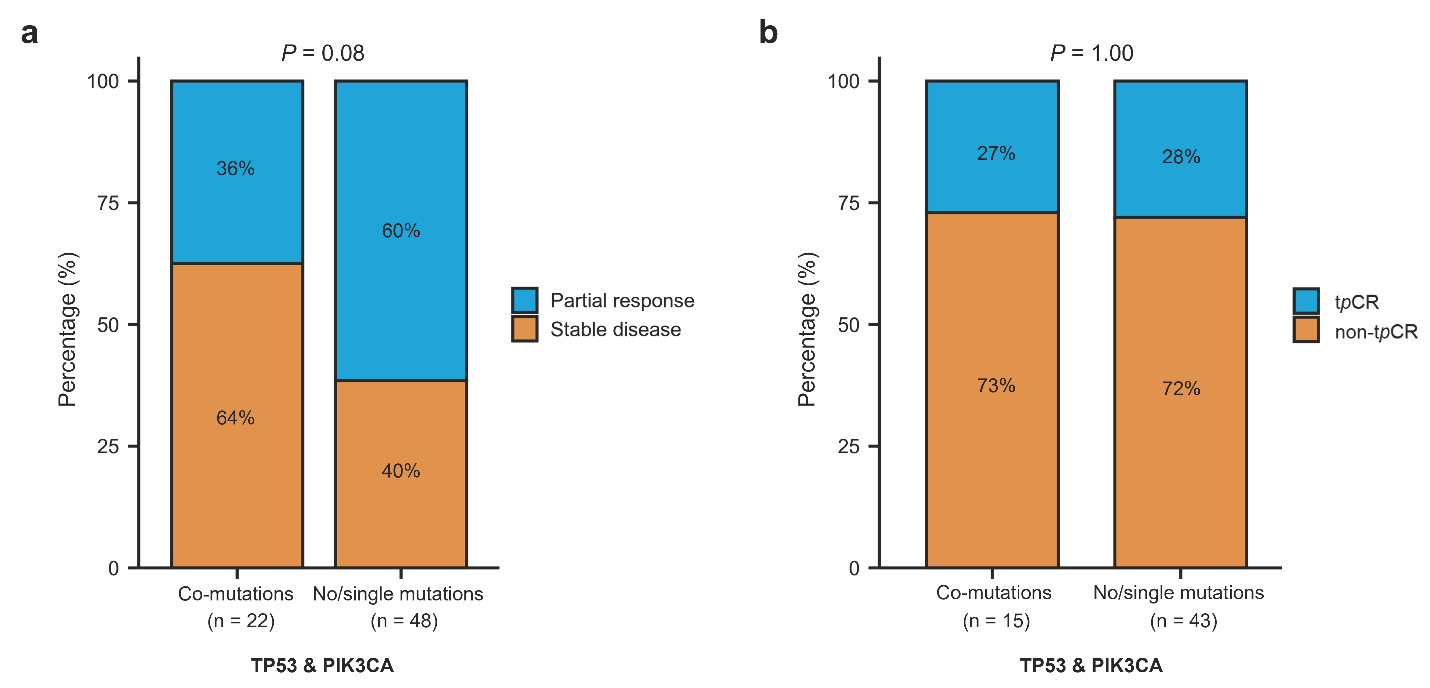
**

**Fig. 3**. Association of treatment responses and pathological complete response (*p*CR) with mutation status of TP53 and PIK3CA pathways in HER2-positive breast cancer patients. The rates of partial response and non-response (*stable disease*) after 2 cycles of treatment (**a**), and the rates of total pCR (t*p*CR) and non-t*p*CR after 6 cycles of treatment (**b**), were calculated for patients stratified by co-mutations or no/single mutations in the TP53 and PIK3CA pathways. *P*-values for each comparison are also shown.


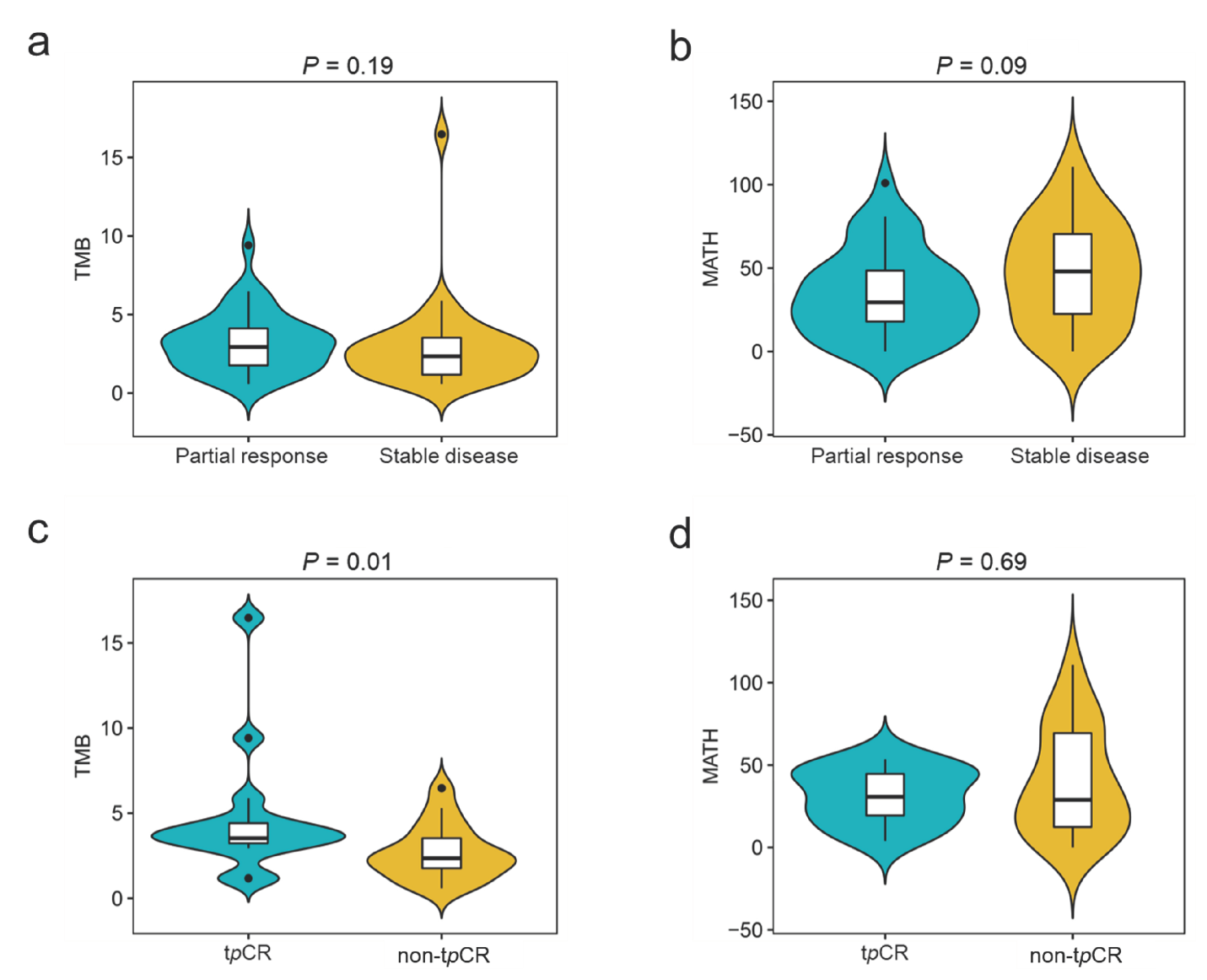


**Fig. 4.** Association of tumour mutation burden (TMB) and mutant allele tumour heterogeneity (MATH) with treatment outcomes in HER2-positive breast cancer patients. The scores of TMB (**a**) and MATH (**b**) of tumour samples from early responders (*partial response*) and non-responders (*stable disease*) after two cycles of neoadjuvant treatment with TCbH, and the scores of TMB (**c**) and MATH (**d**) of tumour samples from patients who achieved total pathological complete response (t*p*CR) and those who did not (non-t*p*CR) after 6 cycles of neoadjuvant treatment, were calculated. *P*-values for each comparison are also shown.

**
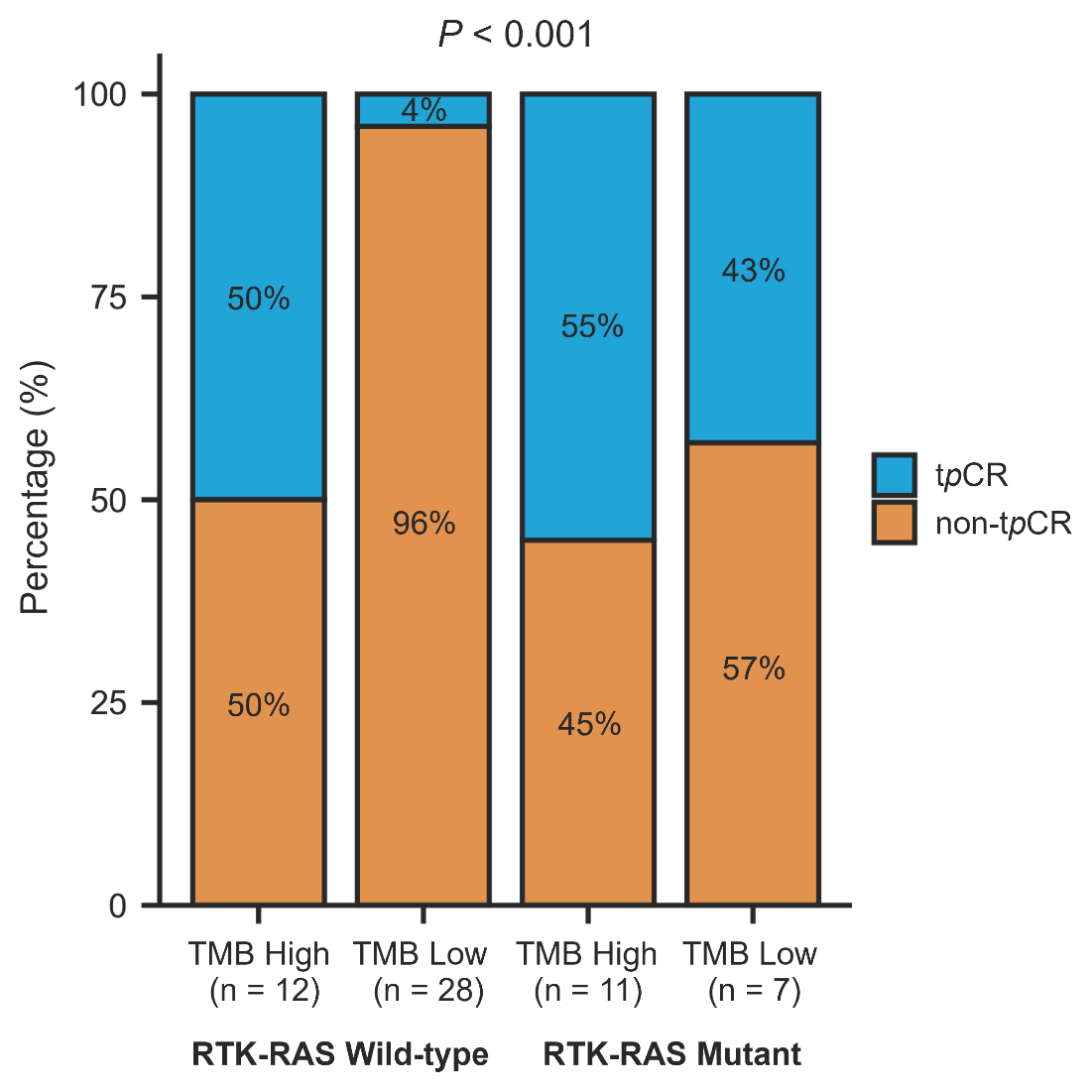
**

**Fig. 5.** Total pathological complete response (t*p*CR) and non-t*p*CR rates in HER2-positive breast cancer patients across all study cohorts who completed six cycles of neoadjuvant TCbH treatment, with or without pyrotinib. The rates are stratified by the combined status of tumour mutation burden (TMB; *high* or *low*) and RTK-RAS pathway mutations (*wild-type* or *mutant*). The number of patients in each subgroup is shown. The *P*-value reflects the comparison between groups with different TMB and RTK-RAS pathway mutation statuses.

**Table 1.** Pathological complete response (*p*CR) in breast cancer patients underwent neoadjuvant therapy stratified by MRI response and clinical characteristics*

| **Clinical Characteristics** | **t*p*CR (%)** | | |  | **b*p*CR (%)** | | |
| --- | --- | --- | --- | --- | --- | --- | --- |
|  | **Cohort A** | **Cohort B** | **Cohort C** |  | **Cohort A** | **Cohort B** | **Cohort C** |
| HR^+^ | 26.2 | 10.0 | 20.0 |  | 38.1 | 15.0 | 20.0 |
| HR^-^ | 40.0 | 33.3 | 43.8 |  | 40.0 | 33.3 | 50.0 |
| HER2 (IHC score 3+) | 30.9 | 16.0 | 36.4 |  | 40.0 | 16.0 | 36.4 |
| HER2 (IHC score 2+) | 28.6 | 0.0 | 0.0 |  | 28.6 | 100.0 | 12.5 |
| Ki67 ≥20% | 37.3 | 10.5 | 34.4 |  | 41.2 | 10.5 | 34.4 |
| Ki67 <20% | 0.0 | 28.6 | 11.1 |  | 27.3 | 42.9 | 22.2 |
| TNM II | 35.6 | 12.5 | 26.7 |  | 37.8 | 12.5 | 26.7 |
| TNM III | 15.6 | 0.0 | 26.1 |  | 18.8 | 0.0 | 30.4 |
| Premenopausal | 15.6 | 0.0 | 26.1 |  | 18.8 | 0.0 | 30.4 |
| Postmenopausal | 46.7 | 23.5 | 33.3 |  | 60.0 | 29.4 | 33.3 |

*Patients in cohort A were MRI responders who received docetaxel, carboplatin and trastuzumab treatment, those in cohort B were MRI non-responders who received docetaxel, carboplatin and trastuzumab treatment, and those in cohort C were MRI non-responders who received docetaxel, carboplatin and trastuzumab in combination with pytotinib treatment.

Abbreviations: *MRI*, magnetic resonance imaging, *tpCR* total *p*CR, *bpCR* breast *p*CR, *HR^+^* hormone receptor-positive, *HR^-^* hormone receptor-negative, *HER2* human epidermal growth factor receptor 2, *IHC* immunohistochemistry

**Table 2.** Baseline characteristics of 70 patients with pre-treatment samples included in the biomarker analysis for early response assessment

| **Characteristics** | **All Patients (n=70)** | **Partial Response**  **(n=37)** | **Stable Disease**  **(n=33)** | ***P*** |
| --- | --- | --- | --- | --- |
| **Age of years (median)** | 53.5 | 54 | 53 | 0.47 |
| **Menopausal status** |  |  |  | 0.47 |
| Yes | 34 | 16 | 18 |  |
| No | 36 | 21 | 15 |  |
| **ER status** |  |  |  | 0.32 |
| Positive | 45 | 26 | 19 |  |
| Negative | 25 | 11 | 14 |  |
| **PR status** |  |  |  | 0.12 |
| Positive | 49 | 29 | 20 |  |
| Negative | 21 | 8 | 13 |  |
| **HER2 (IHC score 3+)** |  |  |  | 1.00 |
| Yes | 61 | 32 | 29 |  |
| No | 9 | 5 | 4 |  |
| **Additional pyrotinib** |  |  |  | < 0.0001 |
| Yes | 22 | 0 | 22 |  |
| No | 41 | 36 | 5 |  |
| Unknown | 7 | 1 | 6 |  |
| **T stage** |  |  |  | 0.82 |
| T0 | 1 | 1 | 0 |  |
| T1 | 7 | 3 | 4 |  |
| T2 | 55 | 30 | 25 |  |
| T3 | 6 | 2 | 4 |  |
| T4 | 1 | 1 | 0 |  |
| **N stage** |  |  |  | 0.18 |
| N0 | 36 | 15 | 21 |  |
| N1 | 23 | 17 | 6 |  |
| N2 | 11 | 5 | 6 |  |
| **TNM stage** |  |  |  | 0.36 |
| II | 54 | 27 | 27 |  |
| III | 15 | 9 | 6 |  |
| IV | 1 | 1 | 0 |  |

Abbreviations: *ER* estrogen receptor, *PR* progesterone receptor, *HER2* human epidermal growth factor receptor 2, *IHC* immunohistochemistry

**Table 3.** Baseline characteristics of 58 patients with pre-treatment samples included in the final analysis of pathological complete response (*p*CR)

| Characteristics | **All patients (n=58)** | **Non-*p*CR (n=42)** | ***p*CR (n=16)** | ***P*** |
| --- | --- | --- | --- | --- |
| Age of years (median) | 53.5 | 53 | 54 | 0.19 |
| Menopausal status |  |  |  | 0.04 |
| Yes | 27 | 16 | 11 |  |
| No | 31 | 26 | 5 |  |
| ER status |  |  |  | 0.20 |
| Positive | 41 | 32 | 9 |  |
| Negative | 17 | 10 | 7 |  |
| PR status |  |  |  | 0.05 |
| Positive | 41 | 33 | 8 |  |
| Negative | 17 | 9 | 8 |  |
| HER2 (IHC score 3+) |  |  |  | 0.42 |
| Yes | 50 | 35 | 15 |  |
| No | 8 | 7 | 1 |  |
| Additional pyrotinib |  |  |  | 0.50 |
| Yes | 22 | 17 | 5 |  |
| No | 34 | 23 | 11 |  |
| Unknown | 2 | 2 | 0 |  |
| T stage |  |  |  | 0.73 |
| T0 | 1 | 0 | 1 |  |
| T1 | 7 | 5 | 2 |  |
| T2 | 44 | 33 | 11 |  |
| T3 | 5 | 3 | 2 |  |
| T4 | 1 | 1 | 0 |  |
| N stage |  |  |  | 0.02 |
| N0 | 31 | 19 | 12 |  |
| N1 | 17 | 13 | 4 |  |
| N2 | 10 | 10 | 0 |  |
| TNM stage |  |  |  | 0.07 |
| II | 45 | 30 | 15 |  |
| III | 13 | 12 | 1 |  |

Abbreviations: *ER* estrogen receptor, *PR* progesterone receptor, *HER2* human epidermal growth factor receptor 2, *IHC* immunohistochemistry

**Table 4.** Multivariable-adjusted odds ratios of pathway mutations associated with pathological complete response (*p*CR) in patients who received 6 cycles of neoadjuvant treatment (n = 58)

| **Characteristics** | ***No. of patients achieved p*CR**^*^ **(n/N)** | **Odds Ratio** | ***P*** |
| --- | --- | --- | --- |
| **Response after 2 cycles of treatment** |  |  | 0.44 |
| Partial Response | 11/31 | 1.00 |  |
| Stable Disease | 5/27 | 0.57 (0.14 – 2.35) |  |
| **Tumour mutation burden** |  |  | 0.03 |
| Low | 4/35 | 1.00 |  |
| High | 12/23 | 5.38 (1.24 – 23.36) |  |
| **DNA damage response pathway** |  |  | 0.60 |
| Wild Type | 8/40 | 1.00 |  |
| Mutated | 8/18 | 1.48 (0.34 – 6.40) |  |
| **RTK-RAS pathway** |  |  | 0.09 |
| Wild Type | 7/40 | 1.00 |  |
| Mutated | 9/18 | - 1. (0.82 – 12.77) |  |

^*^A total of 16 patients achieved *p*CR after 6 cycles of neoadjuvant chemotherapy.

**REFERENCES**

1. Chalmers, Z. R. et al. Analysis of 100,000 human cancer genomes reveals the landscape of tumor mutational burden. *Genome Med.* **9**, 34 (2017).

2. Weiss, G. J. et al. Tumor cell-free dna copy number instability predicts therapeutic response to immunotherapy. *Clin. Cancer Res.* **23**, 5074-5081 (2017).

3. Mroz, E. A., Tward A. D., Hammon R. J., Ren Y. & Rocco J. W. Intra-tumor genetic heterogeneity and mortality in head and neck cancer: analysis of data from the Cancer Genome Atlas. *PLoS Med.* **12**, e1001786 (2015).
