# Supplementary material for: Neoadjuvant pyrotinib and trastuzumab in HER2-positive breast cancer with no early response (NeoPaTHer): efficacy, safety and biomarker analysis of a prospective, multicentre, response-adapted study
Source: Signal Transduct Target Ther. 2025 Jan 29;10:45. doi: 10.1038/s41392-025-02138-6 (PMC11775149; doi:10.1038/s41392-025-02138-6)

**Neoadjuvant Pyrotinib and Trastuzumab in HER2-positive Breast Cancer with No Early Response (NeoPaTHer): Primary Analysis of a Prospective, Multicenter, Response-adapted Study**

**Study Protocol**

**Version 1.2.1**

**March 30^th^，2020**

**Sponsor：The Second Hospital of Shandong University**

**Primary investigator：Pro. Zhigang Yu**

Catalogue

[Abstract 1](#_TOC_250007)

[List of Abbreviations 1](#_TOC_250006)6

1 Study Background 19

1.1 Overview of Breast Cancer and HER2 Targeted Therapy 19

1.2 Drug name and physical and chemical properties 22

1.3 Pharmacological types and mechanisms of action of pyrotinib 22

1.4 Preclinical pharmacodynamic study of pyrotinib 22

1.5 Preclinical Toxicological Study of Pyrotinib 23

1.6 Preclinical Pharmacokinetic Study of Pyrotinib 23

1.7 Progress of Clinical Research on Pyrotinib 23

1.8 Research basis 24

2 Study Objectives and Result Indicators 30

2.1 Study Objectives 30

2.2 Research Result Indicators 30

3 Research Design 31

4 Participant Selection and Withdrawal Criteria 33

4.1 Eligibility Criteria 33

4.2 Exclusion Criteria 34

4.3 Withdrawal of Participants 35

4.4 Termination Criteria 36

5 Study Drugs 37

5.1 Overview of Study Drugs 37

5.2 Methods of Use and Precautions for Study Drugs 38

5.3 Management, Distribution, and Recovery of Trial Drugs 46

5.4 Concomitant Treatment 46

6 Content of Research Evaluation 48

6.1 Description of Evaluation Content 49

6.2 Research Evaluation Timeline 53

6.3 Subject Withdrawal, Treatment Discontinuation, and Termination of the Study and Research Center 55

7 Safety Evaluation 57

7.1 Physical Examination and Vital Signs 57

7.2 Laboratory Tests 57

7.3 Electrocardiogram 58

7.4 Echocardiography 59

7.5 Adverse Events (AEs) 59

7.6 Serious Adverse Events (SAE) 62

7.7 Pregnancy 66

8 Study Management 66

8.1 Ethical Norms and Informed Consent 66

8.2 Trial Drug Management 67

8.3 Protocol Revision 67

8.4 Quality Control and Assurance 67

9 Data Management 68

9.1 Data Recording 68

9.2 Data Management 69

9.3 Protocol Deviations 69

9.4 Data Preservation 70

10 Data Analysis and Statistical Methods 70

10.1 Sample Size Estimation 70

10.2 Statistical Analysis 70

11 Research Schedule 70

12 References 70

[Attachment 1: Clinical Staging Criteria for Breast Cancer（AJCC Breast Cancer TNM Staging）](#_TOC_250005)  75

Attachment 2: ECOG Performance Status Scale 76

[Attachment 3: Criteria for Evaluation of Solid Tumor Response](#_TOC_250004) 77

[Attachment IV: Nomogram for Measuring Body Surface Area](#_TOC_250003) 93

[Attachment V: Standard Guidelines for Surgical Sampling after Neoadjuvant Therapy](#_TOC_250002) 94

[Attachment 6: Flowchart for Breast Tissue Sampling](#_TOC_250001) 98

**Protocol Signature Page**

As a participating doctor/statistical analyst of the research, I have read the protocol of this research and fully discussed the purpose of the research and the content of this scheme with the research leader.

I agree to conduct the research according to this protocol, abide by its requirements, comply with ethical norms, and carry out this clinical research under the guidance of Good Clinical Practice (GCP).

I agree to keep the content of this protocol confidential and will not disclose it to any third party. The content of the protocol will only be used for conducting this research.

I understand that if the research is terminated or suspended in advance at any time for any reason, I will be notified in writing. Similarly, if I decide to withdraw from conducting this research, I will immediately notify the head unit of the research group and the main researcher in writing.

- Data Privacy Policy: I am aware that the leading unit collects and uses my personal and professional information that I have provided and discloses it to internal trial managers. This information will be recorded in the database and only used for the sole purpose of initiating the research center and conducting the research.

Signature:

Date:

**Abstract**

| **Title** | Neoadjuvant Pyrotinib and Trastuzumab in HER2-positive Breast Cancer with No Early Response (NeoPaTHer): Primary Analysis of a Prospective, Multicenter, Response-adapted Study |
| --- | --- |
| **Version** | 1.2.1 |
| **Sponsor** | The Second Hospital of Shandong University |
| **Primary Investigator** | Pro. Zhigang Yu |
| **Study Drug** | Pyrotinib Maleate Tablets (hereinafter referred to as Pyrotinib)  Trastuzumab Injection (Herceptin®) (hereinafter referred to as Trastuzumab)  Docetaxel Injection (Aisu®) (hereinafter referred to as Docetaxel)  Carboplatin Injection (hereinafter referred to as Carboplatin) |
| **Study Categories** | A prospective, open and exploratory study |
| **Study Object** | Patients with HER2-positive early or locally advanced primary breast cancer who have not achieved partial response (PR) after receiving two cycles of neoadjuvant treatment with trastuzumab combined with docetaxel and carboplatin based on imaging assessment. |
| **Study Background** | Approximately 20% to 30% of breast cancer patients have the HER2 gene amplification/overexpression. Trastuzumab combined with chemotherapy can improve the pCR rate and prognosis of patients, establishing the therapeutic status of neoadjuvant therapy for HER2-positive breast cancer. Dual-target blockade with trastuzumab combined with pertuzumab or trastuzumab combined with lapatinib significantly increased the PCR rate. Meanwhile, in the NeoALTTO subgroup study, the early metabolic effect of anti-HER2 therapy on primary tumors and its predictive value for pCR were evaluated. The study screened neoadjuvant treatment options and efficacy through early efficacy evaluation by MRI (based on the RECIST 1.1 efficacy evaluation criteria for solid tumors). |
| **Study Purpose** | Screening neoadjuvant treatment options based on early response to treatment via MRI for the analysis of neoadjuvant efficacy and safety. |
| **Primary Outcome Measures** | **curative effect index**  **Main curative effect index:**  **pCR rate:** Assess the pCR rate of patients with SD evaluated by imaging after two cycles of neoadjuvant TCbH treatment, who then completed six cycles of neoadjuvant therapy combined with pyrotinib, and had no invasive disease in the breast and axillary lymph nodes at surgery (ypT0/isN0).  Secondary endpoint indicators:  PCR rate (ypT0/isN0) in different treatment groups (Cohort A: MRI-assessed PR; Cohort B/C MRI-assessed SD)  bPCR rate (ypT0/is) in different treatment groups (Cohort A: MRI-assessed PR; Cohort B/C MRI-assessed SD)  Objective response rate (ORR): Defined as the proportion of subjects who achieve CR or PR as the best tumor response during neoadjuvant therapy. The investigator will evaluate the ORR based on the Response Evaluation Criteria in Solid Tumors (RECIST 1.1).  Event-free survival (EFS): Defined as the time interval from randomization to the first recorded related event, which includes preoperative disease progression, postoperative disease recurrence, and death from any cause.  Disease-free survival (DFS): The time interval from the first day of disease-free (i.e., surgery date) to the first recorded related event, which includes postoperative disease recurrence and death from any cause.  **Safety indicators:**  ECOG score, vital signs, physical examination, laboratory test indicators (routine blood, urine, and stool tests, blood biochemistry, pregnancy test, and virological screening), ECG, echocardiography, adverse events (AE), with AE graded according to the NCI-CTC AE 5.0 standard.  **Exploratory indicators:**  Utilize tumor tissue and peripheral blood for DNA sequencing to explore the ability of biologically relevant genes and genetic signatures (GSs) to predict the efficacy of anti-HER2 drugs. |
| **Overall Study Design** | This study is an open, multicenter, non-controlled, exploratory clinical study.  HER2-positive (centrally confirmed) operable early or locally advanced breast cancer patients initially received two cycles of neoadjuvant trastuzumab combined with docetaxel and carboplatin. Patients who achieved PR (based on RECIST 1.1 criteria) on imaging (MRI) evaluation continued the original regimen (Cohort A) for four additional cycles until surgery. Patients who did not achieve PR were divided into two groups based on patient preference: continuing the original regimen (Cohort B) and pyrotinib combined with the original regimen (Cohort C). All treatment groups received four additional cycles of treatment until surgery, with efficacy assessments performed every two cycles.  Trastuzumab, docetaxel, and carboplatin will be administered intravenously on the first day of each neoadjuvant cycle, with a cycle length of 21 days for a total of six cycles. The treatment duration is calculated based on the start and end dates of trastuzumab, docetaxel, and carboplatin administration. Pyrotinib in the experimental group will be administered orally at a dose of 400 mg daily from the first day of the third cycle to the 21st day of the sixth cycle.  **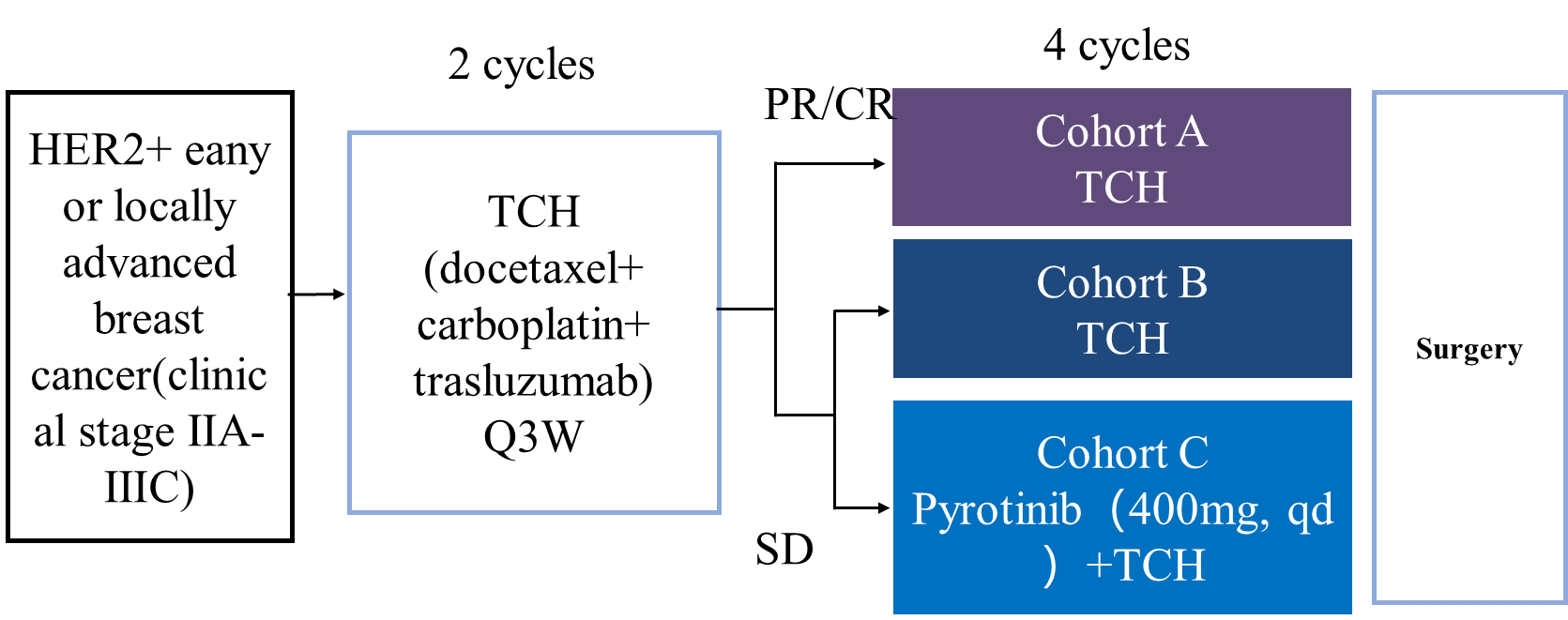**  **Research overall design diagram** |
| **Dosage Regimen** | Pyrotinib: In the experimental group, it will be administered continuously from the first day of the third neoadjuvant cycle to the 21st day of the sixth cycle, once daily at a dose of 400mg, taken orally within 30 minutes after breakfast.  All subjects will receive trastuzumab, docetaxel, and carboplatin intravenously (IV) on the first day of each neoadjuvant cycle, with a cycle length of 21 days for a total of six cycles (at least four cycles for the control group):  Trastuzumab: 8mg/kg loading dose in the first cycle, 6mg/kg in cycles 2-6, administered intravenously on day 1, with a 21-day cycle length, for a total of six cycles.  Docetaxel: 75mg/m^2^, administered intravenously on day 1, with a 21-day cycle length, for a total of six cycles. Preventive use of granulocyte colony-stimulating factor (G-CSF) before administration is required to reduce the risk of hematological toxicity.  Carboplatin: AUC=5, administered intravenously on day 1, with a 21-day cycle length, for a total of six cycles. Preventive use of granulocyte colony-stimulating factor (G-CSF) before administration is required to reduce the risk of hematological toxicity.  Dosage adjustments may be made according to the subject's adverse reactions as specified in the protocol.  During the study, the continuous pause time for pyrotinib and the cumulative pause time for each cycle should not exceed 14 days. Multiple pauses due to adverse events are allowed throughout the entire treatment period. |
| **Eligibility Criteria** | To be enrolled in this trial, subjects must meet all the following inclusion criteria:   1. Female patients with newly diagnosed breast cancer, aged ≥18 years and ≤75 years. 2. ECOG score of 0 to 1. 3. Breast cancer that meets the following criteria:：   --Histologically confirmed invasive breast cancer with a primary tumor diameter > 2cm measured by standard assessment methods at the research center.  --Tumor stage: Early (T2-3, N0-1, M0) or locally advanced (T2-3, N2 or N3, M0).  4. Pathologically confirmed HER2-positive breast cancer, defined as an immunohistochemistry (IHC) score of 3+ in >10% of immunoreactive cells or in situ hybridization (ISH) results showing HER2 gene amplification (HER2 gene signal-to-centromere 17 signal ratio ≥ 2.0 or HER2 gene copy number ≥ 6).  5. Breast MRI examination is required at baseline, after two cycles of neoadjuvant therapy, and before surgery.  6. Known hormone receptor status (ER and PgR).  7. Functional levels of major organs must meet the following requirements (no blood transfusion or use of leukocyte or platelet-elevating drugs within 2 weeks before screening):  1) Blood routine:  Absolute neutrophil count (ANC) ≥ 1.5×10^9^/L;  Platelet count (PLT) ≥ 90×109/L;  Hemoglobin (Hb) ≥ 90g/L.  2) Blood biochemistry:  Total bilirubin (TBIL) ≤ upper limit of normal (ULN);  Alanine aminotransferase (ALT) and aspartate aminotransferase (AST) ≤ 1.5×ULN;  Alkaline phosphatase ≤ 2.5×ULN;  Blood urea nitrogen (BUN) and creatinine (Cr) ≤ 1.5×ULN.  3) Echocardiography:  Left ventricular ejection fraction (LVEF) ≥ 55%.  4) 12-lead electrocardiogram:  Fridericia-corrected QT interval (QTcF) < 470msec.  8. For premenopausal or non-surgically sterilized female patients: Agree to abstain from sexual activity or use effective contraception during the treatment period and for at least 7 months after the last dose of treatment.  9. Voluntarily participate in this study, sign the informed consent form, have good compliance, and are willing to cooperate with follow-up visits. |
| **Exclusion Criteria** | Any individual with any of the following conditions will not be eligible as a subject:   1. Stage IV(metastatic) breast cancer. 2. Inflammatory breast cancer. 3. Prior anti-cancer treatment or radiation therapy for any malignancy, excluding malignancies such as cured cervical carcinoma in situ, basal cell carcinoma, or squamous cell carcinoma. 4. Concurrent receipt of anti-cancer therapy in other clinical trials, including endocrine therapy, bisphosphonate therapy, or immunotherapy. 5. Undergone major surgeries unrelated to breast cancer within 4 weeks before enrollment, or the patient has not fully recovered from such surgeries. 6. Severe heart disease or discomfort, including but not limited to:   -A confirmed history of heart failure or systolic dysfunction (LVEF < 50%);  -High-risk uncontrolled arrhythmias, such as atrial tachycardia, resting heart rate > 100bpm, significant ventricular arrhythmias (e.g., ventricular tachycardia), or higher-grade atrioventricular block (i.e., Mobitz II second-degree AV block or third-degree AV block);  -Angina pectoris requiring anti-angina medication;  -Clinically significant heart valve disease;  -ECG showing transmural myocardial infarction;  -Poorly controlled hypertension (systolic blood pressure > 180mmHg and/or diastolic blood pressure > 100mmHg).   1. Inability to swallow, intestinal obstruction, or other factors that affect drug administration and absorption. 2. A known history of allergy to any component of the drugs in this protocol: a history of immune deficiency, including HIV-positive status, or suffering from other acquired or congenital immune deficiency diseases, or a history of organ transplantation. 3. Pregnant or lactating female patients, fertile female patients with a positive baseline pregnancy test, or fertile female patients who are unwilling to take effective contraceptive measures during the entire trial period and within 7 months after the last dose of study medication. 4. Having severe comorbid conditions or other comorbidities that may interfere with the planned treatment, or any other situation deemed unsuitable for participation in this study by the investigator. |
| **Sample Size and Statistical Methods** | Assuming the pCR rate for group B in this study is 12%, and the pCR rate for group C is 35%, with a 1:2 random enrollment ratio, using PASS 15 software and the Z-test, setting α=0.05 (one-sided) and β=0.20, the experimental group and the control group would need to enroll (32 and 63) subjects respectively. Considering a 10% dropout rate, the final determined sample sizes for the experimental group and the control group are (35 and 70) subjects respectively.  The efficacy data will be evaluated in the Per-Protocol Set (PPS). All enrolled subjects with at least one medication record will be included in the Safety Set. The 95% confidence interval for the pCR rate will be calculated using the Wilson method. Different statistical analysis methods will be adopted based on the data type. Chi-square test will be used to analyze categorical variables, while the distribution of continuous variables will first be assessed through the Shapiro-Wilk test, followed by the Mann-Whitney U test or independent sample t-test for evaluation. Multivariate logistic regression will be employed to identify independent factors associated with pCR. All statistical analyses will be conducted using SPSS (version 22.0). P < 0.05 will be considered statistically significant. |
| **Study Progress** | Estimated first participant enrollment time: Q3 2020  Estimated last participant enrollment time: Q3 2022 |

####
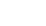
 Visit Schedule

|  |  | Neoadjuvant Therapy | | | | | | Surgical Operation ^a^ | Follow-up OF Survival^b^ |
| --- | --- | --- | --- | --- | --- | --- | --- | --- | --- |
| Visit Time | 28-day Screening Period | 1^st^ day of cycle 1 | 1st day of cycle 2 | 1st day of cycle 3 | 1^st^ day of cycle 4 | 1^st^ day of cycle 5 | 1^st^ day of cycle 6 | Within 14 days after the end of cycle 6 | Every 3 months (±28 days) for the first year, and then every 6 months (±28 days) afterwards. |
| Informed Consent Form | **×** |  |  |  |  |  |  |  |  |
| Demographic Data | **×** |  |  |  |  |  |  |  |  |
| General Medical History | **×** |  |  |  |  |  |  |  |  |
| Complete Physical Examination^c^ | **×** |  |  |  |  |  |  | **×**（Before Surgery） |  |
| Vital Signs^d^ | **×** | **×** | **×** | **×** | **×** | **×** | **×** | **×**（Before Surgery） |  |
| Weight^e^ | **×** | **×** | **×** | **×** | **×** | **×** | **×** | **×**（Before Surgery） |  |
| Height^e^ | **×** |  |  |  |  |  |  |  |  |
| ECOG Score | **×** |  |  |  |  |  |  |  |  |
| Hematology Examination^f^ | **×** | **×** | **×** | **×** | **×** | **×** | **×** | **×**（Before Surgery） |  |
| Blood Biochemical Tests^g^ | **×** | **×** | **×** | **×** | **×** | **×** | **×** | **×**（Before Surgery） |  |
| Cell-free DNA (cf-DNA) | **×** |  |  | **×** |  | **×** |  | **×**（手术前） | **×** |
| Coagulation Function Test^h^ | **×** |  |  | **×** |  | **×** |  |  |  |
| Urine Routine Test^i^ | **×** |  |  |  |  |  |  | **×**（Before Surgery） |  |
| Infectious Disease Screening^j^ | **×** |  |  |  |  |  |  |  |  |
| Pregnancy Test^k^ | **×** |  |  |  |  |  |  | **×**（Before Surgery） |  |
| Bone Scan^l^ | **×**^m^ |  |  |  |  |  |  |  |  |
| Mammography | **×** |  |  |  |  |  |  | **×**（Before Surgery） |  |
| Breast Examination & Ultrasound | **×** |  | **×** | **×** | **×** | **×** | **×** | **×**（Before Surgery） |  |
| Breast MRI | **×**（before biopsy） |  |  | **×** |  | **×** |  | **×**（Before Surgery） |  |
| Imaging Evaluation | **×**^n^ |  |  | **×**^n^ |  |  |  | **×**（Before Surgery）^n^ | **×**^n,o^ |

|  |  | Neoadjuvant Therapy | | | | | | Surgical Operation ^a^ | Follow-up OF Survival^b^ |
| --- | --- | --- | --- | --- | --- | --- | --- | --- | --- |
| Visit Time | 28-day Screening Period | 1st day of cycle 1 | 1st day of cycle 2 | 1st day of cycle 3 | 1st day of cycle 4 | 1st day of cycle 5 | 1st day of cycle 6 | Within 14 days after the end of cycle 6 | Every 3 months (±28 days) for the first year, and then every 6 months (±28 days) afterwards. |
| Skin tattoo and/or surgical metal clips for marking the location of the primary tumor lesion^p^ | **×** |  |  |  |  |  |  |  |  |
| 12-lead ECG ^q^ | **×**^m^ |  | **×** |  | **×** |  | **×** | **×**（Before Surgery） |  |
| ECHO^m^ | **×**^r^ |  | **×**^r^ |  | **×**^r^ |  | **×**^r^ |  |  |
| Tumor hormone receptor status | **×** |  |  |  |  |  |  |  |  |
| Pathological examination of the primary tumor | **×** |  |  |  |  |  |  |  |  |
|  |  |  |  |  |  |  |  |  |  |
| Pathological response rate assessment |  |  |  |  |  |  |  | **×**（After Surgery） |  |
| Pyrotinib^s^ |  |  |  | **×** | | | |  |  |
| Trastuzumab^t^ |  | **×** | | | | | |  |  |
| Docetaxel^u^ |  | **×** | | | | | |  |  |
| Carboplatin^v^ |  | **×** | | | | | |  |  |
| Adverse events^w^ | **×** | | | | | | |  |  |
| Concomitant medications/treatments^x^ | **×** | | | | | | |  |  |
| Progression/death events | **×** | | | | | | | | |
| Subsequent anti-tumor therapy^y^ | **×** |  |  |  |  |  |  |  | **×** |
| Brain MRI^z^ |  |  |  |  |  |  |  |  | **×** |

CT = Computed Tomography; ECD = Extracellular Domain; ECHO = Echocardiography; ECOG = Eastern Cooperative Oncology Group; eCRF = Electronic Case Report Form; MRI = Magnetic Resonance Imaging; ULN = Upper Limit of Normal.

Note: Except for the first cycle, all visits and evaluations should be conducted within 3 days of the scheduled visit time. On the treatment day, all evaluations should be conducted before administration. If the implementation time required by the trial protocol encounters holidays or weekends, it will be postponed to the nearest date for completion.

Clinical examination results obtained before obtaining the informed consent form and within 28 days before enrollment (laboratory tests only accept results within 7 days) can be used in the study, and such tests do not need to be repeated during screening.

a. A follow-up visit for research treatment completion will be conducted after the completion of neoadjuvant therapy and before surgery. If the research treatment is discontinued due to any reason during neoadjuvant therapy, an exit visit inspection must be conducted according to the visit items at this time node.

b. Starting from the day of surgery, survival follow-up will be conducted every 3 months (±28 days) in the first year and every 6 months (±28 days) afterwards until disease progression or recurrence occurs, or until 3 years after the last subject is randomized (whichever occurs first).

c. The comprehensive physical examination includes the evaluation of the head, eyes, ears, nose, throat, skin, skeletal muscles, respiratory organs, cardiovascular system, gastrointestinal tract, urinary and reproductive system, and nervous system. Abnormalities at baseline will be recorded in the comprehensive medical history and baseline CRF. Clinically significant abnormalities will be recorded in the adverse event CRF. The examination will be performed once during the screening period and before surgery (if not performed within the previous 7 days).

d. Vital signs include respiratory rate, pulse, systolic and diastolic blood pressure (the subject is seated), and body temperature. Vital signs should be obtained and evaluated before each administration of research treatment. The examination will be performed once during the screening period, on the first day of each neoadjuvant treatment cycle, and before surgery.

e. Weight will be measured on the first day of each treatment cycle and compared with baseline weight. If the weight change exceeds ±10%, the trastuzumab and chemotherapy drug doses will be recalculated. Baseline height will be measured, and if the investigator believes that the subject's height may have changed, it should be remeasured.

f. Routine blood tests include hemoglobin, red blood cell count, platelet count, white blood cell count, and neutrophil count. Hematological evaluations should be performed during the screening period, before administration on the first day of each neoadjuvant treatment cycle (or within 3 days before), and before surgery.

g. Blood biochemistry tests include sodium, potassium, chloride, calcium, magnesium, cholesterol, triglycerides, glucose, BUN or urea, creatinine, total protein, albumin, alkaline phosphatase, ALT, AST, GGT, total bilirubin, and direct bilirubin. Hematological evaluations should be performed during the screening period, before administration on the first day of each neoadjuvant treatment cycle (or within 3 days before), and before surgery.

h. Coagulation function tests will be completed during the screening period.

i. Urine routine tests include urine protein, urine glucose, and urine occult blood. The tests will be performed once during the screening period and before surgery. If urine routine tests show urine protein ++ or above, please add a 24-hour urine protein quantitative test.

j. Infectious disease screening includes hepatitis B five-item test, HIV antibody, and HCV antibody detection. The test will be performed once during the screening period.

k. Pregnancy test: Female subjects of childbearing age need to undergo blood HCG testing during the screening period to exclude pregnancy. The test will be performed once during the screening period and before surgery.

l. Without the use of radioactive isotopes, MRI scanning (with gadolinium imaging if needed) is a suitable method to evaluate bone metastasis.

m. Clinical examination results obtained before obtaining the informed consent form and within 28 days before enrollment do not need to be repeated during screening.

n. According to the principles of diagnosis and treatment of each research center, imaging detection methods (CT or MRI scanning) targeting the target organ will be used to evaluate tumor status during the study. Imaging assessments will be evaluated according to the principles of RECIST 1.1. Tumor imaging assessments will be performed during the screening period, before grouping, before surgery, and during treatment follow-up. The investigator may add necessary imaging examinations based on the subject's actual condition.

o. Starting from the day of surgery, assessments will be conducted every 3 months (±28 days) in the first year and every 6 months (±28 days) afterwards, as well as at the end of the study or during early termination visits.

p. Before neoadjuvant therapy, tumors will be marked using standard methods (such as skin tattoos or surgical metal clips) of each research center.

q: For a 12-lead ECG, observe heart rate, QT, QTc, and P-R intervals. Perform 3 tests during the screening period (with a minimum interval of 10 minutes), and the average of the 3 QTcF values will be used as the baseline QTcF. Perform 1 test each during the screening period, before the start of the 2nd and 4th neoadjuvant treatment cycles, and before surgery. If QTcF increases by >30msec compared to baseline, or if the absolute QTcF value is ≥470 msec in any specified ECG measurement, 2 additional ECG tests need to be performed (with a minimum interval of 10 minutes).

r: Perform ECHO once during the screening period, before the start of the 2nd, 4th, and 6th neoadjuvant treatment cycles.

s: Pyrotinib: Participants will receive continuous pyrotinib treatment by oral administration from day 1 of the 3rd neoadjuvant treatment cycle to day 21 of the 6th treatment cycle, once daily at 400mg, within 30 minutes after breakfast.

t: Trastuzumab: Participants will receive trastuzumab as neoadjuvant therapy through IV infusion every 3 weeks for 6 cycles (cycle 1 loading dose of 8mg/kg, cycles 2-6 at 6mg/kg).

u: Docetaxel: Participants will receive docetaxel (initial dose of 75 mg/m2) through IV infusion in cycles 1-6 (every 3 weeks). Prophylactic use of granulocyte colony-stimulating factor (G-CSF) is required before administration to reduce the risk of hematological toxicity.

v: Carboplatin: Participants will receive carboplatin (AUC=5) through IV infusion in cycles 1-6 (every 3 weeks). Prophylactic use of granulocyte colony-stimulating factor (G-CSF) is required before administration to reduce the risk of hematological toxicity.

w: Report all adverse events from the time the patient signs the consent form until 28 days (+7 days) after the last neoadjuvant treatment dose. Additionally, researchers should report all serious adverse events. Researchers should follow up on each adverse event until it resolves to baseline or better, is deemed stable by the researcher, the participant is lost to follow-up, or the participant withdraws consent. Serious adverse events related to the study drug or caused by the trial procedures should be followed up as much as possible until final results can be reported.

x: Concomitant medications/treatments: Record concomitant medications from 28 days before randomization until the end of the 6th treatment cycle. Once a participant discontinues trial treatment, only record concomitant medications and treatments used for new or unresolved adverse events related to the trial treatment.

y: Subsequent antitumor therapy: Record other antitumor therapies after surgery until disease recurrence, metastasis, or study termination.

z: During the follow-up phase, assess the incidence of brain metastases.

# List of Abbreviations

| Abbreviation Index |
| --- |
| ADL (Activities of Daily Living) |
| AE (adverse event) |
| AKP/ALP (Alkaline Phosphatase) |
| ALB (albumin) |
| ALT (alanine aminotransferase) |
| ANC (absolute neutrophil count) |
| AST (aspartate aminotransferase) |
| AUC (area under concentration time curve) |
| BMI (Body Mass Index) |
| BUN (blood urea nitrogen) |
| CBR（clinical benefit response） |
| CFDA (Chinese Food and Drug Administration) |
| CHOL (cholestenone) |
| CI（Confidence Interval） |
| Cmax (peak concentration) |
| Cr (creatinine) |
| CR (Complete Response) |
| CRF (case report form) |
| DBIL (direct bilirubin) |
| DDFS(Distance Disease Free Survival) |
| DFS(Disease Free Survival) |
| dL (deciliter) |
| DLT (dose limited toxicity) |
| DoR (Duration of Response) |
| DPD (Dihydropyrimidine Dehydrogenase) |
| EC (ethics committee) |
| ECOG (Eastern Cooperative Oncology Group) |
| EDC (electronic data collection) |
| EFS(Events-Free Survival) |
| EGF（Epidermal Growth Factor） |
| EGFR (epidermal growth factor receptor) |

| Abbreviation Index |
| --- |
| EOT(end of treatment) |
| FAS (Full analysis set) |
| FISH (fluorescence in situ hybridization) |
| g (gram) |
| GCP (good clinical practice) |
| h (hour) |
| Hb (hemoglobin) |
| HCV (Hepatitis C Virus) |
| HER2 (human epidermal growth factor receptor 2) |
| HIV (Human Immunodeficiency Virus) |
| HR (heart rate) |
| IBIL (indirect bilirubin) |
| IC50 (50% Inhibition Concentration) |
| ITT (intend to treat) |
| IU (international unit) |
| IV (intravenous) |
| IWRS（Interactive Web Response System） |
| kg (kilogram) |
| L（Liter） |
| LC (lymphocyte cont) |
| LDH (lactate dehydrogenase) |
| LVEF（Left Ventricular Ejection Fraction） |
| m (meter) |
| min (minute) |
| mg (milligram) |
| ml (milliliter) |
| mm (millimeter) |
| ms (Millisecond) |
| MTD (maximum torlerate dose) |

| Abbreviation Index |
| --- |
| NCCN (National Comprehensive Cancer Network) |
| NCI-CTC (national cancer institute common terminology criteria) |
| ORR (Objective Response Rate) |
| OS(Overall Survival) |
| pCR(Pathological Complete Response) PD (Pharmacodynamics) |
| PK (pharmacokinetics) |
| PLT (platelet) |
| PPS (Per Protocol Set) |
| PR（Partial Response） |
| PR（Progesterone receptor） |
| RBC (red blood cell) |
| RTK (Receptor Tyrosine Kinase) |
| SAE (serious adverse event) |
| SAP (statistical analysis plan) |
| SBP (systolic blood pressure) |
| SD (Stable Disease) |
| SS (Safety Set) |
| TBIL (total bilirubin) |
| TG (triglyceride) |
| Tmax (peak time) |
| TP (plasma total protein)  tpCR(totally pathological Complete Response) |
| μmol（micromole） |
| ULN (upper normal limit) |
| VEGF（Vascular Endothelial Growth Factor） |
| WBC (white blood cell) |

**1 Study Background**

**1.1** **Overview of Breast Cancer and HER2 Targeted Therapy** Breast cancer is one of the most common malignant tumors among women, and its incidence rate has been increasing year by year. It is estimated that there were approximately 1.67 million newly diagnosed cases of breast cancer worldwide in 2012. The incidence and mortality rates of breast cancer worldwide vary by geographical distribution, with developed regions having a higher incidence rate (73.4/100,000) than less developed regions (31.3/100,000). However, the mortality rate in developed regions (14.9/100,000) is close to that in less developed regions (11.5/100,000) ^[1]^. Possible reasons include more comprehensive early screening and diagnostic systems in developed regions, as well as more newly approved drugs. In China, breast cancer ranks as the first in the incidence rate of female cancers, with an incidence rate of approximately 37.86/100,000 ^[2]^, and the onset age is younger than in Western countries. Additionally, China has identified breast cancer as a priority for prevention by establishing a healthcare system that focuses on early detection and diagnosis to enable patients to receive earlier treatment.

In the 1980s, scientists discovered that HER2 overexpression is directly related to tumor invasive growth and poor prognosis ^[3]^. Approximately 10% to 34% of patients with invasive breast cancer exhibit overexpression or gene amplification of human epidermal growth factor receptor 2 (HER2, also known as erbB2, neu, and p185HER2) ^[4]^. To date, no ligand has been found to bind to the HER2 receptor. The HER2 molecule primarily forms heterodimers with other receptors in the epidermal growth factor receptor (EGFR) family, further activating pathways such as MAPK, JAK, PI3K, and STAT3, promoting the occurrence and progression of tumor diseases. The HER2 gene is expressed at low levels in normal epithelial cells but is amplified/overexpressed in over 30% of human tumors, including breast cancer, gastric cancer, lung cancer, and others. The HER2 molecule is an independent factor for poor prognosis in breast cancer, and HER2 has been widely considered as a target for drug therapy. With the standardization of HER2 amplification detection, targeted therapy for HER2-positive patients has become a hotspot in basic and clinical research ^[5]^. Currently, several drugs targeting HER2 have been approved and marketed worldwide (see Table 1 for details).。

**Table 1 Currently available HER2-targeted molecular drugs**

|  | Drug Name | Developer | US approval Year | Indications | PRC Approval Year |
| --- | --- | --- | --- | --- | --- |
| Macromolecular Drug | Trastuzumab | Roche | 1998  Herceptin | HER2+ breast cancer；HER2+ Metastatic gastric cancer and gastroesophageal junction adenocarcinoma | 2002 Herceptin |
|  | Pertuzumab | Roche | 2012  Perjeta | Combination therapy with trastuzumab and docetaxel for HER2+ advanced (metastatic) breast cancer; Used for neoadjuvant therapy in early stages | 2018 Perjeta |
| Antibody-Drug Conjugate (ADC) | T-DM1 | Roche | 2013  Kadcyla | Monotherapy for HER2+ metastatic breast cancer after treatment with trastuzumab, taxanes, or a combination of both | —— |
| First-generation Small Molecule Inhibitor | Lapatinib | GlaxoSmithKline (GSK) | 2007  Tykerb | Used in combination with capecitabine for HER2+ metastatic breast cancer after treatment with trastuzumab, taxanes, or a combination of both; Used in combination with letrozole for postmenopausal ER+, HER2+ metastatic breast cancer | 2013 Tykerb |
| Second-generation Small Molecule Inhibitor | Neratinib | Puma | 2017  Nerlynx | Adjuvant therapy and advanced systemic therapy for breast cancer, Phase III clinical study |  |

Among Chinese breast cancer patients, about 20% to 30% have amplification/overexpression of the HER2 gene. The fluorescence in situ hybridization (FISH) results showed that 42.6% (1342 out of 3149 cases) of patients were HER2 positive (2+/3+), while the positive results of immunohistochemistry (IHC) were 46.9% (1477 out of 3149 cases) ^[6]^. Currently, all HER2 targeted drugs available in China are imported drugs, including trastuzumab and lapatinib. According to the "Expert Consensus on Clinical Diagnosis and Treatment of Human Epidermal Growth Factor Receptor 2 (HER2)-Positive Breast Cancer 2016" ^[7]^, for the anti-HER2 treatment of HER2-positive early or locally advanced breast cancer in China, whether it is preoperative neoadjuvant therapy or postoperative adjuvant therapy, trastuzumab-containing treatment is the preferred choice. Even if patients experience recurrence or metastasis, trastuzumab-containing treatment is still the primary treatment option for first-line anti-HER2 therapy. For second-line treatment, in addition to choosing lapatinib as an optional targeted therapeutic drug, trastuzumab combined with chemotherapy is still used as the main treatment method or only the chemotherapy regimen can be adjusted. Due to the high price of imported drugs, the use of HER2 targeted drugs is greatly limited. The "Survey on the Current Status of Diagnosis and Treatment of HER2-positive Breast Cancer in China" released at the 2015 China Society of Clinical Oncology (CSCO) Annual Meeting showed that the targeted drug treatment rate in cities with medical insurance coverage was 57%, while the targeted drug treatment rate in non-medical insurance cities was only 28%. For patients who can obtain the above-mentioned standard treatment containing targeted drugs, even if recent disease control and long-term survival benefits are guaranteed, when the disease progresses, intolerable adverse reactions occur, or for other reasons, it is still inevitable to stop and change the medication. Therefore, the listing and application of domestic HER2 targeted drugs has become an urgent problem to be solved at present.

**1.2 Drug name and physical and chemical properties**

【English Name】Pyrotinib


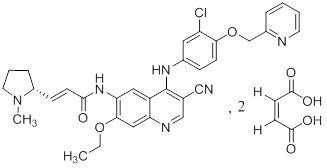


Figure 1 Chemical structure of pyrotinib

【Molecular Formula】C32H31ClN6O3·2C4H4O4

【Molecular weight】815.22

**1.3** **Pharmacological types and mechanisms of action of pyrotinib**

Pyrotinib is a small-molecule, irreversible tyrosine kinase inhibitor that inhibits epidermal growth factor receptor (EGFR) and human epidermal growth factor receptor 2 (HER2). Pyrotinib covalently binds to the ATP binding site of the kinase domain of EGFR and HER2 within the cell, preventing the formation of homodimer and heterodimer of EGFR and HER2 in tumor cells, inhibiting their autophosphorylation, blocking the activation of downstream signaling pathways, and thus inhibiting tumor cell growth.

**1.4 Preclinical pharmacodynamic study of pyrotinib**

Pyrotinib has significant inhibitory effects on EGFR and HER2 at the molecular level, with half maximal inhibitory concentrations (IC50) of 5.6 nM and 8.1 nM respectively. It has a strong inhibitory effect on the proliferation of tumor cells with high expression of HER2, with IC50 ranging from 1 to 43 nM. It can also significantly inhibit the phosphorylation of EGFR and HER2, and has an inhibitory effect on the activation of downstream signals ERK1/2 and Akt, with irreversible inhibitory activity. Pyrotinib can significantly induce BT-474 cells to arrest in the G1 phase of the cell cycle.

Pyrotinib significantly inhibits the growth of tumor models with high HER2 expression such as SK-OV-3, Calu-3, and BT-474, with a significant dose-dependent inhibitory effect and leading to partial tumor shrinkage. Its in vivo and in vitro antitumor effects are superior to or equivalent to the positive control drug HKI-272, as detailed in Table 2; therefore, preclinical data supports the further clinical study of pyrotinib.

Table 2 Pyrotinib's inhibitory effect on tyrosine kinase

| Kinase name |  | IC50(nM,mean±SD) |
| --- | --- | --- |
|  | Pyrotinib | Neratinib（HKI-272） |
| HER2 | 8.1 ± 2.3 | 6.8 ± 0.9 |
| EGFR1 | 5.6 ± 3.9 | 4.2± 0.9 |
| c-Src | 790.3±190.7 | 1159.1±1036.5 |
| KDR | >3,000 | >3,000 |
| c-Kit | >3,000 | >3,000 |
| PDGFRβ | >3,000 | >3,000 |
| C-Met | >3,000 | >3,000 |

**1.5** **Preclinical Toxicological Study of Pyrotinib** Including safety pharmacology, acute toxicity, long-term toxicity, and genetic toxicology studies, the results are detailed in the "Pyrotinib Maleate Investigator's Brochure".

**1.6** **Preclinical Pharmacokinetic Study of Pyrotinib** Multiple studies have been completed, including absorption tests in rats, pharmacokinetic studies in beagle dogs, plasma protein binding rate tests, tissue distribution tests in rats, and in vitro metabolic tests. Details are provided in the "Pyrotinib Maleate Investigator's Brochure".

**1.7 Progress of Clinical Research on Pyrotinib** Jiangsu Hengrui Medicine Co., Ltd. obtained approval from the National Medical Products Administration (NMPA, formerly CFDA) in May 2012 to start Phase I clinical development of pyrotinib. In November 2015, CFDA approved the clinical approval for conducting Phase II/III clinical studies. On June 30, 2015, the Phase I clinical development of pyrotinib was initiated in the United States (U.S. Investigational New Drug [IND] Application Number: 126107). Currently, Jiangsu Hengrui Medicine Co., Ltd. has conducted 14 clinical studies on pyrotinib in China: 7 Phase I clinical studies, including 4 studies in healthy subjects, 2 studies in HER2-positive metastatic breast cancer (mBC) subjects (Study BLTN-Ib, Study BLTN-Ic), and 1 study in advanced gastric cancer subjects (Study BLTN-Id); 1 Phase I/II clinical study in mBC subjects (HR-BLTN-I/II-mBC); 1 ongoing Phase II clinical study in HER2-mutant non-small cell lung cancer (NSCLC) (HR-BLTN-II-NSCLC Study); 3 ongoing Phase III clinical studies in mBC subjects (HR-BLTN-III-MBC-A Study, HR-BLTN-III-MBC-B Study, and HR-BLTN-III-MBC-C Study) and 2 ongoing Phase III studies in eBC subjects, including 1 adjuvant study (HR-BLTN-III-EBC) and 1 neoadjuvant study (HR-BLTN-III-NeoBC). Currently, there is 1 ongoing Phase I clinical study in the U.S., targeting HER2-positive solid tumors and HER2-mutant NSCLC subjects who have progressed after previous HER2-targeted therapy.

According to the published results of current studies, 38 cases were exposed to pyrotinib in Phase I studies, 65 cases in Phase II studies, 186 cases in the Phase III PHENIX study, and 134 cases in the Phase III PHOEBE study. Additionally, among foreign subjects receiving pyrotinib, 31 were HER2-mutant NSCLC subjects, and 31 were subjects with other solid tumors.

The above content provides an overview of the current clinical research results of pyrotinib in China.

**1.8** **Research basis**

**1.8.1 Neoadjuvant therapy research basis** Neoadjuvant therapy for breast cancer has been widely used in clinical practice. Before the emergence of neoadjuvant targeted therapy and neoadjuvant endocrine therapy, neoadjuvant therapy mainly referred to neoadjuvant chemotherapy. Neoadjuvant chemotherapy has indeed benefited some patients, enabling some patients who cannot undergo breast-preserving surgery to have the opportunity for breast-preserving surgery, and enabling some inoperable patients to obtain surgical opportunities.

Now, the scope of neoadjuvant therapy has expanded to include neoadjuvant targeted therapy and neoadjuvant endocrine therapy. A large number of clinical trials have confirmed that targeted therapy has significant efficacy in the adjuvant and advanced treatment of HER2-positive breast cancer, which has also promoted its development in neoadjuvant therapy. Neoadjuvant therapy is the standard treatment for locally advanced breast cancer, which has long been used to shrink tumors to make inoperable tumors operable, increase the rate of breast-preserving surgery, and reduce axillary staging, thereby avoiding axillary dissection and only requiring sentinel lymph node biopsy ^[8][9],[10]^. Pathological complete response (pCR) in neoadjuvant therapy is associated with disease-free survival (DFS) and overall survival (OS) in early breast cancer. The correlation between pathological response and long-term survival in patients with early breast cancer is strongest in patients with triple-negative breast cancer, followed by HER2-positive patients, and least in hormone receptor-positive patients ^[8],[11]^. By providing tumor specimens and blood samples before and during systemic treatment, neoadjuvant therapy allows testing of novel treatments and predictive biomarkers. Neoadjuvant studies allow rapid evaluation of drug efficacy, accelerating the development and approval of treatment strategies for early breast cancer ^[12],[13][14]^. Patients with HER2 overexpression have poor responses to endocrine therapy and standard chemotherapy. The application of anti-HER2 monoclonal antibody trastuzumab in postoperative adjuvant and advanced breast cancer has improved the outcomes of these patients ^[15],[16],[17]^. Currently, in clinical practice of neoadjuvant therapy, trastuzumab combined with chemotherapy can significantly improve the pCR rate and improve patient outcomes, establishing the current standard mode of neoadjuvant therapy for HER2-positive breast cancer. At the same time, a large number of ongoing or completed clinical trials are also striving to explore the efficacy potential of other molecular targeted drugs combined with chemotherapy in neoadjuvant therapy, including lapatinib, neratinib, and a series of small-molecule TKI targeted drugs. In recent years, the dual blockade anti-HER2 treatment model using dual targeted drugs has become particularly popular in adjuvant therapy and neoadjuvant therapy. The efficacy and safety data are constantly being verified in clinical trials, and some treatment modes have obtained clinical approval. At the same time, clinical experts are also making new attempts and explorations. Postoperative adjuvant systemic therapy for breast cancer is currently the standard treatment mode for early and locally advanced breast cancer, aiming to reduce the risk of recurrence after breast cancer surgery. For patients with early or locally advanced breast cancer who hope to surgically remove the lesion as much as possible, preoperative neoadjuvant systemic therapy can shrink the tumor, increasing the possibility of complete removal of the lesion. On the other hand, clinical research evidence has shown that preoperative chemotherapy can increase the rate of breast-preserving surgery and improve the local aesthetics of patients ^[22],[23]^. Currently, this treatment mode is becoming increasingly common in clinical practice. In clinical practice, preoperative treatment can help oncologists assess the impact of treatment regimens on tumor remission, discontinue ineffective treatments, or replace another systemic treatment. At the same time, the remission of tumors after neoadjuvant therapy can provide prognostic information, complementing traditional treatment prognostic data such as initial staging, tumor grade, and hormone receptor status. Finally, neoadjuvant therapy provides researchers with a unique biomarker clinical detection opportunity, examining changes in tumor tissue markers from biopsy to breast cancer surgery after preoperative systemic therapy. A meta-analysis of approximately 4,000 patients comparing neoadjuvant therapy to adjuvant chemotherapy or endocrine therapy showed that the order of systemic therapy and surgery did not alter distant recurrence or overall survival ^[24]^. It is worth noting that patients receiving neoadjuvant therapy have an increased risk of local recurrence compared to those receiving postoperative adjuvant therapy, which is attributed to the omission of final local therapy in some neoadjuvant trials ^[24]^. Assuming that final local therapy is provided, preoperative systemic therapy can be used as an alternative to postoperative systemic therapy for patients with early breast cancer, and neoadjuvant therapy is also an important goal for accelerating new drug development. After neoadjuvant therapy is completed, surgery is performed on the patient, the surgical specimens are reviewed, and the pathological remission of the treatment is assessed. The desired outcome of neoadjuvant therapy is pCR. Evidence suggests that patients who achieve pCR have a survival advantage ^[25], [26], [27], [28]^, therefore, it is believed that the pCR rate is suitable as the primary endpoint for neoadjuvant therapy studies. Currently, there are two definitions of pCR: 1) generally refers to the absence of histological evidence of malignant tumors in the primary breast lesion, or only the presence of carcinoma in situ (ypT0/is), known as bpCR (breast pathological complete response); 2) strictly speaking, it refers to the complete pathological response (pCR) achieved in both the primary breast lesion and the metastatic regional lymph nodes (ypT0ypN0, ypT0/isypN0), known as tpCR (totally pathological complete response). In recent years, many large clinical trials in neoadjuvant therapy have adopted the above definitions of pCR. The Neosphere ^[29]^ and NeoALTTO ^[30]^ trials defined pCR as ypT0/is, while the GeparQuinto ^[31]^ and NOAH ^[32]^ trials defined pCR as ypT0ypN0, and the BIG01-06/EGF106903 ^[33]^ trial defined pCR as ypT0/isypN0. In the comprehensive clinical trial analysis established by the US FDA, CTNeoBC ^[34]^, a meta-analysis of clinical trials on neoadjuvant chemotherapy before 2015 was conducted, comparing the correlation between different definitions of pCR and long-term survival benefits. The analysis results showed that the PFS (progression-free survival) and OS (overall survival) of ypT0ypN0 and ypT0/isypN0 were significantly improved compared to ypT0/is, while the PFS and OS of ypT0ypN0 and ypT0/isypN0 were similar. However, other studies have shown different results. A comprehensive analysis by the German GBG and AGO-B research institutions of 7 prospective clinical trials on neoadjuvant chemotherapy involving 6,377 cases, using the neoadjuvant chemotherapy regimen of 3CEF+3T, found that patients with ypT0ypN0 achieved longer survival benefits compared to those with ypT0/isypN0, with significant improvements in DFS (disease-free survival) and OS ^[35]^. This indicates that pCR should be strictly defined as ypT0ypN0, but such a definition is difficult to achieve clinically. Therefore, in clinical practice, the definition of pCR often adopts ypT0ypN0 or ypT0/isypN0.

In this study, the pCR refers to the complete pathological response achieved in both the primary breast lesion and the metastatic regional lymph nodes.

**1.8.2 Trastuzumab-based Therapy** Various studies have evaluated the role of trastuzumab in neoadjuvant therapy for HER2-positive breast cancer, as follows:

| **Study** | **Preoperative Treatment** | **Postoperative Treatment** |
| --- | --- | --- |
| NeoSphere[**[29]**](#_bookmark30) | （Docetaxel + Trastuzumab  ±pertuzumab）×4 cycles | 5-FU+ Epirubicin + Cyclophosphamide×3 cycles Trastuzumab ×1 year |
| CHER- LOB[**[36]**](#_bookmark37) | （Taxol+ Trastuzumab± Lapatinib）×3 cycles→（5- FU+ Epirubicin + Cyclophosphamide + Trastuzumab±Lapatinib）×4 cycles | Treatment as recommended by the clinician |
| NASBP B-41  [**[37]**](#_bookmark38) | （Doxorubicin + Cyclophosphamide）×4 cycles→（Taxol + Trastuzumab±Lapatinib）×4 cycles | Trastuzumab 52 weeks |
| CALGB 40601[**[38]**](#_bookmark39) | （Taxol + Trastuzumab±Lapatinib）×16 weeks | （Doxorubicin + Cyclophosphamide）×4 cycles Trastuzumab×36 weeks |
| NeoALTTO[**[30]**](#_bookmark31) | （Trastuzumab±Lapatinib）×6 cycles  →（Taxol+ Trastuzumab±Lapatinib）×12 weeks | （5-FU+ Epirubicin + Cyclophosphamide）×3cycles, Trastuzumab×34weeks |

**1.8.3 Rationale for Docetaxel Dosage** The benefit-risk ratio of docetaxel (100mg/m^2^ every 3 weeks) combined with trastuzumab is positive compared to docetaxel alone in the treatment of HER2-overexpressing metastatic breast cancer patients ^[19]^. That is, adding anti-HER2 targeted therapy to chemotherapy alone brings clinical benefits to patients. In clinical practice, based on the treatment purpose and patient characteristics, there are three recommended doses of docetaxel from monotherapy to combination therapy, including 60, 75, and 100mg/m^2^, each with its suitable population. Low doses can be used to treat patients who are frail or have specific tolerability issues ^[39]^. There is literature evidence showing that when the dosage of docetaxel is 75mg/m^2^, the incidence of febrile neutropenia in Asian patients is significantly higher than in other populations. If tolerability is good, it can be increased to 100mg/m^2^. This finding is consistent with the general observations on paclitaxel tolerability in such populations ^[40], [41]^. In fact, the registered dosage of docetaxel in Asian countries is lower than that in Western countries. To ensure the efficacy and safety of subjects during the study, the recommended starting dosage of docetaxel combined application is 75mg/m^2^. Meanwhile, preventive use of granulocyte colony-stimulating factor (G-CSF) is recommended at the initial administration to reduce the risk of drug-induced hematological toxicity.

**1.8.4 Rationale for Carboplatin Dosage**  Carboplatin is a second-generation platinum complex that is widely used due to its strong antitumor activity, low gastrointestinal reactions, and low renal toxicity. It can bind to DNA, form cross-links, disrupt DNA function, and prevent it from replicating and synthesizing, exerting a good killing effect on tumor cells in the growth phase. It is a non-specific drug for the cell cycle. Relevant studies on the treatment of breast cancer with docetaxel combined with carboplatin have shown that carboplatin dosage AUC 5 or 6 achieves good therapeutic effects with fewer adverse reactions, thus a carboplatin dosage of AUC 5/6 is recommended. Meanwhile, preventive use of granulocyte colony-stimulating factor (G-CSF) is also recommended at the initial administration to reduce the risk of drug-induced hematological toxicity.

**1.8.5 Rationale for Trastuzumab Dosage (Data from Herceptin® Instructions)** Studies on trastuzumab in the treatment of metastatic breast cancer have shown that short-term intravenous administration of trastuzumab at 10, 50, 100, 250, and 500 mg once a week exhibits dose-dependent pharmacokinetics. As the dose level increases, the average half-life extends, and the clearance rate decreases. The average half-lives at the 10 and 500 mg dose levels are 1.7 and 12 days, respectively. The distribution volume of trastuzumab is approximately equal to the serum volume (44 mL/kg). In the study, the average peak serum concentration of trastuzumab at the highest weekly dose (500 mg) level was 377 µg/ml. In clinical trials, trastuzumab was administered with an initial loading dose of 4 mg/kg and a weekly maintenance dose of 2 mg/kg. The observed average half-life was 5.8 days (ranging from 1 to 32 days). Between 16 and 32 weeks, the serum concentration of trastuzumab reached a steady state, with an average trough concentration and peak concentration of approximately 79 µg/mL and 123 µg/mL, respectively. In a study on trastuzumab used as adjuvant therapy for breast cancer women, after an initial loading dose of 8 mg/kg, a 6 mg/kg treatment every 3 weeks was administered. It was observed that the average half-life of trastuzumab was 16 days (range: 11-23 days). From the 6th to the 37th week of the study, the serum concentration of trastuzumab reached a steady state, with an average trough concentration and peak concentration of 63 µg/mL and 216 µg/mL, respectively. In this study, trastuzumab will be administered using a three-week treatment regimen, with an initial loading dose of 8 mg/kg followed by 6 mg/kg every 3 weeks.

## **1.8.6 Basis for MRI Evaluation of Neoadjuvant Early Therapeutic Effect** Currently, breast MRI is the imaging examination method with the highest sensitivity and accuracy for evaluating the therapeutic effect of neoadjuvant therapy. It is significantly superior to clinical palpation, X-ray, and ultrasound in distinguishing residual lesions from fibrosis and detecting small residual lesions in breast cancer after neoadjuvant therapy. In the NCCN guidelines, MRI is used as an evaluation method for breast cancer before and after preoperative systemic therapy to determine the extent of the tumor, response to treatment, and the feasibility of breast-saving therapy. Previous studies have shown that MRI is the most sensitive method for evaluating and predicting the therapeutic response to neoadjuvant chemotherapy (NAC). The clinical response after the initial cycle of neoadjuvant chemotherapy is associated with pathological complete response (pCR) ^[42]^. In the PHERGain trial evaluating after one NAT cycle, the pCR rate was higher in responders than in non-responders (Group A, 65.6% vs. 10.0%, p=0.013) ^[43]^.

**2** **Study Objectives and Result Indicators**

**2.1** **Study Objectives**

**2.1.1** **Primary study objective**

To increase the pathological complete response (pCR) rate of achieving no invasive disease in the breast and axillary lymph nodes (ypT0/isN0) after four cycles of neoadjuvant therapy with pyrotinib combined with trastuzumab emtansine (TCbH) in patients with HER2-positive breast cancer who have achieved stable disease (SD) after two cycles of TCbH neoadjuvant imaging assessment.

**2.1.2 Secondary study objective**

To evaluate the efficacy of dual-target therapy with pyrotinib plus trastuzumab combined with docetaxel and carboplatin compared to trastuzumab combined with docetaxel and carboplatin in the treatment of early-stage or locally advanced HER2-positive breast cancer in newly diagnosed patients, based on the following endpoints:

-Event-free survival (EFS)

-Disease-free survival (DFS)

-Distant disease-free survival (DDFS)

-Objective response rate (ORR) assessed according to the Response Evaluation Criteria in Solid Tumors (RECIST 1.1)

**2.1.3 Safety Objective**

To evaluate the safety and tolerability of each treatment regimen.

**2.2** **Research Result Indicators**

**2.2.1 Primary Efficacy Indicators**

pCR (ypT0/is, ypN0) assessed pathologically, defined as the absence of any residual invasive cancer in the pathological evaluation of excised breast cancer samples and all ipsilateral lymph node samples stained with hematoxylin and eosin after completion of neoadjuvant therapy and surgery.

**2.2.2 Secondary Efficacy Indicators:**

-pCR rate (ypT0/isN0) in different treatment groups (Cohort A: MRI-assessed PR; Cohort B/C: MRI-assessed SD)

-bPCR rate (ypT0/is) in different treatment groups (Cohort A: MRI-assessed PR; Cohort B/C: MRI-assessed SD)

-Objective Response Rate (ORR): Defined as the proportion of subjects achieving CR or PR as the best tumor response during neoadjuvant therapy. The ORR will be evaluated by investigators according to the Response Evaluation Criteria in Solid Tumors (RECIST 1.1).

-Event-free Survival (EFS): Defined as the time interval from randomization to the first recorded related event, including preoperative disease progression, postoperative disease recurrence, and death from any cause.

-Disease-free Survival (DFS): The time interval from the first day of disease-free status (i.e., the date of surgery) to the first recorded related event, including postoperative disease recurrence and death from any cause.

**2.2.3 Safety Indicators**

-Exposure to trial drugs and the incidence, nature, and severity of adverse events (including serious adverse events).

-ECOG score, vital signs, physical examination, laboratory test indicators (routine blood, urine, and stool tests, blood biochemistry, pregnancy test, and virological screening), ECG, echocardiogram, adverse events (AE), with AE graded according to the NCI-CTC AE 5.0 criteria.

**Exploratory Indicators**

DNA sequencing of tumor tissue and peripheral blood to explore the ability of biologically relevant genes and genetic signatures (GSs) to predict the efficacy of anti-HER2 drugs.

1. **Research Design**

This is a multicenter, exploratory clinical study that plans to enroll patients with HER2-positive early-stage or locally advanced breast cancer who have not achieved partial response (PR) after initial two cycles of neoadjuvant therapy with trastuzumab combined with docetaxel and carboplatin (based on RECIST 1.1 criteria). Patients who achieve PR will continue the original treatment plan for 4 additional cycles until surgery. Patients will be divided into the pyrotinib combined with the original treatment group (experimental group) and the continuation of the original treatment group (control group) based on their preferences. The experimental group will undergo 4 cycles of treatment until surgery, with efficacy evaluations every two cycles. The control group will undergo at least 2 cycles of treatment until surgery. Subjects will receive the investigational drug (pyrotinib) combined with trastuzumab, docetaxel, and carboplatin until completion of post-surgical adjuvant therapy, intolerable toxicity, withdrawal of informed consent, or the investigator's judgment that drug administration must be discontinued. Trastuzumab, docetaxel, and carboplatin will be administered intravenously on the first day of each neoadjuvant cycle, with 21 days as one cycle for a total of 6 cycles (at least 4 cycles for the control group). The duration of the treatment period will be calculated based on the start and end times of trastuzumab, docetaxel, and carboplatin administration. Pyrotinib in the experimental group will be administered continuously at a daily dose of 400mg from the first day of the third neoadjuvant cycle to the 21st day of the sixth cycle.

Subjects' clinical response and disease progression will be evaluated according to RECIST v1.1. Postoperative pathological response will be evaluated according to pathological assessment principles. The efficacy purpose will be assessed after all subjects complete treatment/termination visits. Subjects' safety throughout the study period will be assessed through laboratory tests and adverse event reports.

The overall design diagram of this study is as follows:

**4** **Participant Selection and Withdrawal Criteria**

**4.1** **Eligibility Criteria** To be enrolled in this trial, subjects must meet all the following inclusion criteria:

1) Female patients with newly diagnosed breast cancer, aged ≥18 years and ≤75 years.

2) ECOG score of 0 to 1.

3)Breast cancer that meets the following criteria:

--Histologically confirmed invasive breast cancer with a primary tumor diameter > 2cm measured by standard assessment methods at the research center.

--Tumor stage: Early (T2-3, N0-1, M0) or locally advanced (T2-3, N2 or N3, M0).

4) Pathologically confirmed HER2-positive breast cancer, defined as an immunohistochemistry (IHC) score of 3+ in >10% of immunoreactive cells or in situ hybridization (ISH) results showing HER2 gene amplification (HER2 gene signal-to-centromere 17 signal ratio ≥ 2.0 or HER2 gene copy number ≥ 6).

5) Breast MRI examination is required at baseline, after two cycles of neoadjuvant therapy, and before surgery.

6) Known hormone receptor status (ER and PgR).

7) Functional levels of major organs must meet the following requirements (no blood transfusion or use of leukocyte or platelet-elevating drugs within 2 weeks before screening):

-Blood routine:

Absolute neutrophil count (ANC) ≥ 1.5×10^9^/L;

Platelet count (PLT) ≥ 90×109/L;

Hemoglobin (Hb) ≥ 90g/L.

-Blood biochemistry:

Total bilirubin (TBIL) ≤ upper limit of normal (ULN);

Alanine aminotransferase (ALT) and aspartate aminotransferase (AST) ≤ 1.5×ULN;

Alkaline phosphatase ≤ 2.5×ULN;

Blood urea nitrogen (BUN) and creatinine (Cr) ≤ 1.5×ULN.

-Echocardiography:

Left ventricular ejection fraction (LVEF) ≥ 55%.

-12-lead electrocardiogram:

Fridericia-corrected QT interval (QTcF) < 470msec.

8) For premenopausal or non-surgically sterilized female patients: Agree to abstain from sexual activity or use effective contraception during the treatment period and for at least 7 months after the last dose of treatment.

9) Voluntarily participate in this study, sign the informed consent form, have good compliance, and are willing to cooperate with follow-up visits.

**4.2** **Exclusion Criteria**

Any individual with any of the following conditions will not be eligible as a subject:

1) Stage IV (metastatic) breast cancer.

2) Inflammatory breast cancer.

3) Prior anti-cancer treatment or radiation therapy for any malignancy, excluding malignancies such as cured cervical carcinoma in situ, basal cell carcinoma, or squamous cell carcinoma.

4) Concurrent receipt of anti-cancer therapy in other clinical trials, including endocrine therapy, bisphosphonate therapy, or immunotherapy.

5) Undergone major surgeries unrelated to breast cancer within 4 weeks before enrollment, or the patient has not fully recovered from such surgeries.

6) Severe heart disease or discomfort, including but not limited to:

-A confirmed history of heart failure or systolic dysfunction (LVEF < 50%);

-High-risk uncontrolled arrhythmias, such as atrial tachycardia, resting heart rate > 100bpm, significant ventricular arrhythmias (e.g., ventricular tachycardia), or higher-grade atrioventricular block (i.e., Mobitz II second-degree AV block or third-degree AV block);

-Angina pectoris requiring anti-angina medication;

-Clinically significant heart valve disease;

-ECG showing transmural myocardial infarction;

-Poorly controlled hypertension (systolic blood pressure > 180mmHg and/or diastolic blood pressure > 100mmHg).

7) Inability to swallow, intestinal obstruction, or other factors that affect drug administration and absorption.

8) A known history of allergy to any component of the drugs in this protocol: a history of immune deficiency, including HIV-positive status, or suffering from other acquired or congenital immune deficiency diseases, or a history of organ transplantation.

9) Pregnant or lactating female patients, fertile female patients with a positive baseline pregnancy test, or fertile female patients who are unwilling to take effective contraceptive measures during the entire trial period and within 7 months after the last dose of study medication.

10) Having severe comorbid conditions or other comorbidities that may interfere with the planned treatment, or any other situation deemed unsuitable for participation in this study by the investigator.

**4.3** **Withdrawal of Participants**

**4.3.1 Criteria for Participant Withdrawal from the Clinical Study:**

1) Participants withdraw their informed consent voluntarily at any time;

2) After randomization, it is discovered that the participant has seriously violated the inclusion and exclusion criteria;

Termination of study treatment, but continuation of follow-up as required by the study:

1. Medical imaging indicates disease progression;
2. Participants who are unable to tolerate toxicity even after reducing the dose of pyrotinib or placebo to 240mg/d;
3. Participants who are unable to tolerate toxicity even after reducing docetaxel and carboplatin to the lowest dose judged by the investigator;
4. Pyrotinib suspension for more than 14 days at a time or consecutively within one cycle; docetaxel, carboplatin, and trastuzumab suspension for more than two consecutive cycles;
5. Any clinical adverse event, abnormal laboratory test results, or other medical conditions occur, resulting in the participant no longer benefiting from continued medication;
6. The participant becomes pregnant during the study;
7. Use of prohibited drugs specified in the protocol;

8) Other reasons deemed by the investigator that make continuation of the study medication impossible.

**4.3.2 Handling of Withdrawn Participants**

It is necessary to make every effort to complete the study treatment end/withdrawal visit according to the protocol and conduct safety follow-up and survival follow-up for the participants as stipulated during the follow-up period. For participants who discontinue medication due to non-disease progression and non-fatal reasons, they need to continue to receive efficacy follow-up according to the protocol until disease progression, death, or the start of other anti-tumor treatment. The investigator may recommend or provide new or alternative treatment methods based on the participant's actual situation**.**

**4.4 Termination Criteria**

**4.4.1 Termination Criteria for Each Research Center**

If the sponsor discovers that the investigators at a research center have severely or continuously failed to comply with the protocol and other trial procedures, and this may interfere with the correct implementation of the trial, the sponsor has the right to terminate the trial at that research center. In such a case, the sponsor will immediately notify the investigators and the regulatory authorities of the termination information. If the investigators terminate or suspend the trial at the research center, they must immediately inform the participants and report the reasons in writing to the clinical trial institution/ethics committee of the research center, the sponsor, and the regulatory authorities as required by regulations.

**4.4.2 Termination Criteria for the Entire Trial**

This study may be terminated or suspended prematurely if sufficient reasons arise. If the study is terminated or suspended prematurely, the sponsor must submit a written notification explaining the reasons for the termination or suspension to the investigators, the China Food and Drug Administration (CFDA), and relevant departments. The principal investigator must immediately report to the ethics committee and provide the corresponding reasons.

The termination criteria for this study include but are not limited to the following:

1. Unexpected, significant, or unacceptable risks to participants are discovered.
2. Significant errors in the protocol are found during the trial execution.
3. The study drug/experimental treatment is ineffective, or continuing the trial is meaningless.
4. It is extremely difficult to complete the trial due to reasons such as severe lag in participant enrollment or frequent protocol deviations.

**5. Study Drugs**

**5.1** **Overview of Study Drugs**

**5.1.1 Names and Sources**

Pyrotinib maleate tablets (pyrotinib), an innovative drug marketed by Jiangsu Hengrui Medicine Co., Ltd., under the brand name Aireini®.

Trastuzumab for injection, known as Herceptin®, is produced by Roche Pharmaceuticals.

Docetaxel injection, a traditional anti-tumor drug marketed by Jiangsu Hengrui Medicine Co., Ltd., under the brand name Aisu®, or other commercially available products approved for marketing in China selected by clinicians with GCP qualifications from participating clinical research units based on drug availability and therapeutic consistency. The specific names and sources should be referenced in the instructions and recorded in the participant's diary and original medical records.

Carboplatin injection, specifically selected by clinicians with GCP qualifications from participating clinical research units based on drug availability and therapeutic consistency. The specific names and sources should be referenced in the instructions and recorded in the participant's diary and original medical records.

**5.1.2 Drug Forms and Specifications**

1. Form: Pyrotinib tablets

Specifications: 160mg, 80mg

Packaging: 160mg × 28 tablets, 80mg × 14 tablets

Storage conditions: Sealed, stored in a dry place below 25°C

Expiration date: 24 months

1. Form: Trastuzumab for injection (Herceptin®)

Specifications: 440mg

Packaging: Contains 1 vial of trastuzumab powder 440mg, 1 vial of 20ml sterile water for injection containing 1.1% benzyl alcohol.

Storage conditions: Packaged and transported at 2°C-8°C (36°F-46°F). Immediately place in a refrigerator (same temperature range) upon receipt to ensure optimal physical and biological integrity. The refrigerator temperature must be recorded (as required by local pharmacy regulations) to ensure appropriate storage conditions. Do not freeze.

Expiration date: 48 months

Usage precautions: Trastuzumab is sensitive to shear stress (e.g., stirring or rapid injection from a syringe). Do not shake. Vigorous agitation of trastuzumab solution can cause protein aggregation and form a cloudy solution. Handle trastuzumab with caution during reconstitution. Excessive foaming during reconstitution or shaking the reconstituted trastuzumab solution can cause problems with the dose withdrawn from the vial.

1. Form: Docetaxel injection (Aisu®)

Specifications: 0.5ml: 20mg; 1.5ml: 60mg.

Packaging: 1.5ml: 60mg: Each box contains one vial of docetaxel injection 1.5ml: 60mg and one vial of solvent 4.5ml (containing 13% ethanol); 0.5ml: 20mg: Each box contains one vial of docetaxel injection 0.5ml: 20mg and one vial of solvent 1.5ml (containing 13% ethanol).

Storage conditions: 2-8°C, stored in a sealed and light-resistant container

Expiration date: 24 months (0.5ml: 20mg); 18 months (1.5ml: 60mg)

1. Form: Carboplatin injection

Specifications: 10ml: 50mg

Packaging: 10ml per box

Storage conditions: Stored in a cool place protected from light (not exceeding 20°C)

Expiration date: 24 months

**5.2** **Methods of Use and Precautions for Study Drugs**

**5.2.1 Pyrotinib:**

Subjects will receive continuous oral administration of pyrotinib from Day 1 of the 3rd neoadjuvant treatment cycle to Day 21 of the 6th cycle, taking 400mg once daily within 30 minutes after breakfast. Dosage adjustments may be made based on the subject's adverse reactions according to the protocol. The following situations should be recorded in detail in the original data: If there is a missed dose, record the time when the drug should have been taken and the reason for missing it; if there is vomiting leading to reduced intake, record it in the subject's diary, original medical records, and CRF. In the event of adverse events during the study, investigators should provide active symptomatic treatment and record the combined treatment and medication in detail in the medical records. According to previous clinical trial data of pyrotinib, the tolerability of pyrotinib monotherapy is good. The incidence of grade III/IV adverse events in the 400mg dose group was 25% (2/8), and 11.1% (1/9) in the 320mg dose group, both being diarrhea. Other common adverse reactions include hand-foot syndrome, liver function abnormalities, and vomiting. Investigators should provide medical treatment based on clinical conditions. The following treatment methods are for reference:

Diarrhea: Before the subject starts taking the test drug, the investigator should inform the subject in detail about the possibility of diarrhea and measures for managing it. Provide symptomatic treatment and close follow-up or observation (≤14 days). It is clinically recommended to start oral administration of Montmorillonite Powder on the day of diarrhea, 3g/bag, 3 times daily; for severe diarrhea, oral or intravenous electrolyte infusion may be given. Resume drug administration according to the relevant provisions of Table 3 after the adverse event recovers to grade I or normal.

Hand-foot syndrome: Provide symptomatic treatment and close follow-up. Recommended symptomatic support treatment includes: strengthening skin care, keeping the skin clean to avoid secondary infection; avoiding pressure or friction; using moisturizers or lubricants.

**Table 3 Dose adjustment regulations of pyrotinib**

| **NCI-CTC AE 5.0** | Treatment Management During Therapy（After Active Clinical Treatment or Observation） | Dose Adjustment After Recovery |
| --- | --- | --- |
| Cardiac Toxicity |  |  |
| ≥ Grade II clinically significant LVEF decline/LVEF below lower limit of normal (including asymptomatic LVEF decline ≥ 10% with LVEF < 50%, or heart failure) | Permanent discontinuation | - |
| Diarrhea |  |  |
| Grade IV | Permanent discontinuation | - |
| Grade III | Suspend dosing until recovery to Grade 0-I | First: 400 mg  Second: 320 mg  Third: 240 mg |
| Diarrhea (Grade I-II with complications)（Including but not limited to mild to severe abdominal cramps, ≥ Grade II nausea or vomiting, ECOG score decline, fever, sepsis, neutropenia, bleeding, or dehydration） |
| Other Adverse Events |  |  |
| ≥ Grade II non-hematological adverse events (excluding alopecia, fatigue, asthenia, etc.) | Suspend dosing until recovery to Grade 0-I | First: 400 mg  Second: 320 mg  Third: 240 mg |
| ≥ Grade III | Suspend dosing until recovery to Grade 0-I | First: 400 mg  Second: 320 mg  Third: 240 mg |

LVEF，Left Ventricular Ejection Fraction.

* The grading of hand-foot syndrome and other adverse events not included are specified in Section 7.5.2. Investigators shall provide clinical active treatment or observation (≤14 days) based on the condition of the subjects and the adverse events. If the adverse events persist, it is recommended to adjust the medication according to this table.

After dose adjustment, if the subject still has clinically uncontrollable adverse events (i.e., the adverse events persist after clinical treatment or observation ≤ 14 days, and occur ≥ 2 times), the investigator may judge to further reduce the dose by one gradient when resuming medication after suspension, with the lowest dose being 240 mg of pyrotinib.

Multiple drug suspensions are allowed during the treatment process, and each suspension should be followed by resumption of medication only after the adverse event recovers to Grade 0-I and complications disappear. The continuous suspension time and the cumulative suspension time per cycle of pyrotinib should not exceed 14 days to ensure the drug intensity received by the subject. If the suspension time of pyrotinib due to adverse events exceeds the above standard, the subject will be withdrawn from the study.

**5.2.2 Trastuzumab:**

Trastuzumab is administered intravenously on the first day of every 3-week cycle for a total of 6 cycles (cycles 1-6). Subjects will receive a loading dose of 8 mg/kg trastuzumab in cycle 1, and 6 mg/kg in cycles 2-6. As trastuzumab may cause infusion reaction symptoms such as nausea, fever, diarrhea, chills, fatigue, and headache, such reactions generally occur during or shortly after infusion. Therefore, trastuzumab administration should be performed in an environment with emergency equipment, and medical staff should be trained in monitoring and responding to medical emergencies. Monitor subjects for any adverse reactions during each infusion, and monitor for at least 90 minutes after the first trastuzumab infusion.

If subjects experience infusion-related symptoms (such as fever, chills, headache, fatigue, itching, nausea, vomiting, and diarrhea), the infusion rate of trastuzumab should be slowed or discontinued. Oxygen, β-agonists, antihistamines, antipyretics, and corticosteroid supportive therapy can help alleviate symptoms. Subjects who experience infusion-related symptoms during or after infusion may receive premedication with analgesics and antihistamines for subsequent infusions. Subjects with grade ≥4 allergic reactions or infusion-related acute respiratory distress syndrome should discontinue treatment. As infusion reactions may occur with a delayed onset, subjects should contact their attending physician if any problems arise.

The trastuzumab dose is calculated based on the subject's actual body weight, and the subject's weight should be recorded at baseline and during each visit. If the subject's weight increases or decreases by more than 10% relative to baseline, the dose should be recalculated. A nomogram for calculating body surface area is provided in the appendix (see Appendix IV).

Dose adjustments are not allowed except for those required due to changes in body weight. Treatment may be interrupted or discontinued if toxicity (including cardiac toxicity) occurs. If trastuzumab administration must be delayed for one day or more, all treatments should be postponed by the same amount of time.

If trastuzumab treatment is suspended for more than two cycles or requires complete discontinuation, the subject will be withdrawn from the study.

**5.2.3 Docetaxel**:

Subjects will receive docetaxel at a dose of 75 mg/m2 every 3 weeks for a total of 6 cycles (cycles 1-6). Docetaxel is administered intravenously on the first day of each cycle, after the observation period for trastuzumab infusion. Granulocyte colony-stimulating factor should be administered prophylactically before docetaxel administration to reduce the risk of hematologic toxicity. Subjects will be closely monitored for hypersensitivity reactions starting from the infusion of docetaxel, which may occur within a few minutes. Severe hypotension, bronchospasm, or generalized rash/erythema require immediate discontinuation of docetaxel and appropriate treatment. If milder symptoms such as flushing or localized skin reactions occur, the infusion rate may be slowed. Subjects experiencing severe hypersensitivity reactions should be withdrawn from the study. Pre-treatment medication (consisting of oral corticosteroids) can be given on day -1, 1, and 2 if there are no contraindications, such as oral dexamethasone at a dose of 16 mg/day (divided into two doses of 8 mg each day).

The adjustment of docetaxel dosage will be made in accordance with the treatment principles of each research center (the minimum dosage is 60mg/m2), and the decision to continue the medication or withdraw from all research treatments will be jointly discussed by the researchers and the sponsor. After the dosage reduction, the dosage of the subjects shall not be increased to a higher level. If there is myelosuppression, liver dysfunction, or other dose-limiting toxicities, the administration of docetaxel can be delayed. If the administration of docetaxel must be delayed for one day or more, all treatments will be postponed for the same period of time.

The dosage of docetaxel should be calculated based on the body surface area of the subjects, and the baseline weight and height of the subjects should be recorded. The weight should also be recorded during each scheduled visit. If the researcher believes that the height of the subject may have changed, the height should be re-measured. If the increase or decrease in the subject's weight relative to the baseline is greater than 10%, the dosage should be recalculated. A nomogram for measuring body surface area is provided in the appendix (see Appendix IV). There are guidelines for adjusting the dosage of docetaxel and interrupting or terminating treatment due to toxicity. If the administration of docetaxel must be delayed for one day or more, all treatments will be postponed for the same period of time.

If the suspension time of docetaxel treatment exceeds two courses or if the treatment needs to be completely terminated, the subject will be withdrawn from the study.

**5.2.4 Carboplatin:**

Blood tests and liver/kidney function should be checked before and after using this product. During the treatment period, white blood cells and platelets should be checked at least once to twice per week. Due to its significant suppressive effect on the bone marrow, the drug should not be repeated within 3 weeks after administration. In cases of severe myelosuppression, blood transfusion may be necessary. Blood cells should be checked at the start of treatment and weekly thereafter as a basis for subsequent dosage adjustment. Once an allergic reaction to this product occurs, appropriate treatment measures should be taken immediately. For patients with a creatinine clearance rate of less than 60ml/min, the renal clearance of carboplatin decreases, and the dosage of carboplatin should be appropriately reduced. When used in combination with other anticancer treatments, attention should be paid to making appropriate dosage adjustments. This product is for intravenous injection only, and should be avoided from leaking outside the blood vessel. It should be used within 8 hours, and direct sunlight should be avoided during infusion and storage. Carboplatin may cause a decrease in plasma electrolytes (such as magnesium, potassium, sodium, calcium, etc.), so monitoring should be paid attention to during use. It should be used with caution in patients with chickenpox, herpes zoster, infection, and renal function impairment.

**5.2.5 Response Measures for Adverse Events during Treatment with Trastuzumab, Docetaxel, and Carboplatin**

| **Adverse Events** | **Response Measures** |
| --- | --- |
| Neutropenia:  ANC count < 1,500 cells/μL | Suspend study treatment^*^  If ANC count recovers to ≥1,500 cells/μL within 6 weeks, resume  study treatment^*^  若6周内ANC计数未恢复至≥1500个细胞/μL，中 止研究治疗^*^。 |
| Symptomatic LVSD (heart failure) | Discontinue study treatment^*^ |
| Asymptomatic LVEF decrease | Suspend, continue, or discontinue study treatment according to the instructions in Figure 3 below. |
| Allergic reaction or acute  respiratory distress syndrome:  Grade IV | Discontinue study treatment^*^ |
| Other non-hematological  toxicities: Grade I or II | Grade I: Continue study treatment^*^  Grade II: The investigator may continue study treatment based  on the subject's tolerance, or resume study treatment after the  severity of the event is reduced to ≤ Grade I* |
| Other non-hematological  toxicities: Grade III or IV | Suspend study treatment*。  If the severity of the event is reduced to ≤ Grade I within 6  weeks, resume study treatment*  If the severity of the event is not reduced to ≤ Grade I within 6  weeks, discontinue study treatment* |

ANC = Absolute Neutrophil Count; LVEF = Left Ventricular Ejection Fraction; LVSD = Left Ventricular Systolic Dysfunction; Note: The severity of all events is determined according to NCI CTCAE (v5.0).

* The study treatment refers to trastuzumab combined with docetaxel and carboplatin.

**Figure 3 Illustrated Diagram for the Management of Asymptomatic LVEF Decline**

**
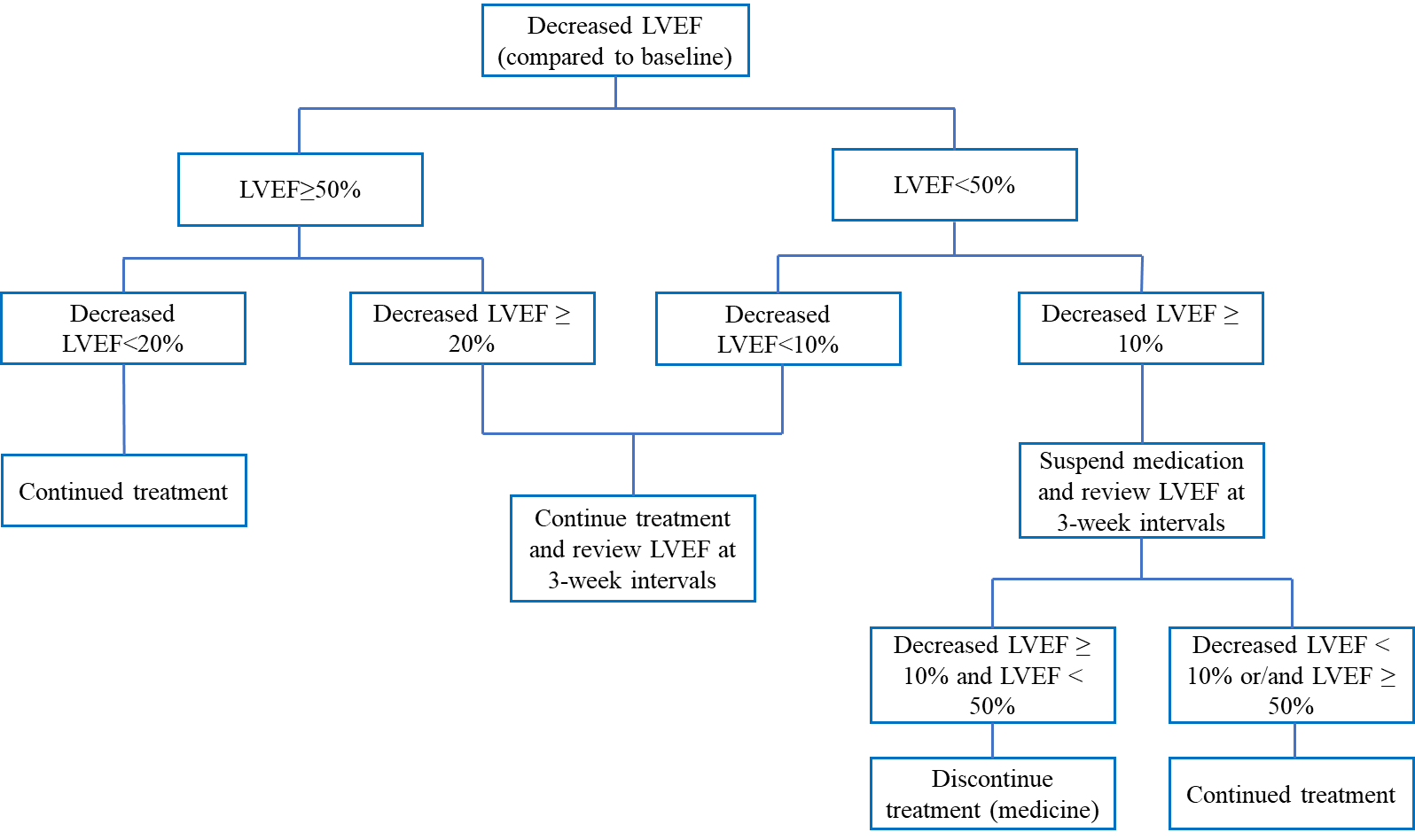
**

**5.3 Management, Distribution, and Recovery of Trial Drugs**

The management, distribution, and recovery of clinical trial drugs in this study are handled by designated personnel. Researchers must ensure that all trial drugs are only used for subjects participating in this clinical trial, and their dosage and usage should comply with the trial protocol. Unused drugs should be returned to the applicant, and clinical drugs must not be transferred to any non-clinical trial participants. Trial drugs should be stored according to label requirements. When distributing drugs, a drug receipt must be signed by two people in duplicate, with one copy retained by the clinical research unit and the other by the applicant. After the study ends, the remaining drugs and empty boxes should be recovered, and both parties should sign a drug recovery form. The distribution and recovery of each drug should be promptly recorded on a special record sheet. The monitor is responsible for monitoring the supply, use, storage, and disposal of clinical trial drugs.

**5.4** **Concomitant Treatment**

Concomitant treatment includes all medications taken by subjects from 28 days before randomization to before surgery, including prescription drugs, non-prescription drugs, natural medicines or homeopathic medications, and nutritional supplements. All concomitant medications should be reported to the researcher and recorded in the Concomitant Medication Case Report Form (CRF).

Any non-protocol treatments or surgical methods during the study period are also considered concomitant treatments and must be recorded. Concomitant medication and treatment information should include date, reason, drug prescription, treatment or method, and any relevant clinical outcomes, with special attention paid to drugs related to adverse events caused by trial drugs.

**5.4.1 Surgery**

After six cycles of neoadjuvant therapy, subjects with surgical indications may receive surgical treatment. Subjects can receive breast-preserving surgery or mastectomy according to routine clinical practice. Before starting neoadjuvant therapy, the primary tumor site should be physically marked using standard clinical practice at each research center (such as skin tattoos or metal clips) to facilitate appropriate surgical resection if the tumor regresses during neoadjuvant therapy.

**5.4.2 Prohibited Treatments During the Study**

Other anticancer treatments besides the anticancer treatment provided in this study, including cytotoxic chemotherapy, radiation therapy (except for adjuvant radiation therapy for breast cancer after chemotherapy), immunotherapy, and biological anticancer treatment;

Other anticancer drugs, including any targeted anticancer therapy (such as lapatinib, neratinib) and traditional Chinese medicine with clear anticancer indications;

Systemic high-dose corticosteroids, referring to dexamethasone with a daily dose of >20 mg (or equivalent doses of other corticosteroids) for continuous treatment of >7 days;

Any other experimental drugs other than the trial drugs used in this study;

Hormonal contraceptives administered orally, by injection, or implantation. If subjects experience adverse reactions, they should be closely observed, given active symptomatic treatment if necessary, and the drugs used should be recorded and described on the CRF form. The following drugs should be used with caution during the study:

Drugs that interfere with liver P450 enzymes or P-gp:

1. Strong inducers of CYP3A4 (such as dexamethasone, phenytoin sodium, carbamazepine, rifampicin, rifabutin, rifapentine, etc.);
2. Strong inhibitors of CYP3A4 (such as ketoconazole, itraconazole, erythromycin, clarithromycin, atazanavir, indinavir, nefazodone, nelfinavir, ritonavir, saquinavir, telithromycin, voriconazole, grapefruit, etc.);
3. Substrates of CYP2C19 (diazepam, imipramine, lansoprazole, and S-mephenytoin);
4. P-gp inhibitors (ritonavir, cyclosporin, verapamil) and inducers (rifampicin)

Drugs that prolong QT interval:

1. Antibiotics (such as clarithromycin, azithromycin, erythromycin, roxithromycin, metronidazole, moxifloxacin, etc.)
2. Antiarrhythmic drugs (quinidine, sotalol, amiodarone, disopyramide, and procainamide, etc.)
3. Antipsychotic drugs (risperidone, fluphenazine, haloperidol, trifluoperazine, thioridazine, pimozide, olanzapine, and clozapine, etc.)
4. Antifungal drugs (fluconazole and ketoconazole, etc.)
5. Antimalarial drugs (mefloquine and chloroquine, etc.)
6. Antidepressant drugs (amitriptyline, imipramine, clomipramine, dothiepin, and doxepin, etc.).

**5.4.3**  **Drugs and treatments that can be combined during the study**

Clinical comorbidities and various AEs should be actively treated and managed. Natural drugs that have been used before entering the study can continue to be used during the study and recorded in the appropriate CRF. All drugs used in combination should be strictly recorded in the CRF in accordance with the provisions of GCP.

**6 Content of Research Evaluation**

Before the study begins, subjects must read and sign the informed consent form (ICF) approved by the current ethics committee (EC). All research evaluations must be conducted within the time window specified in the research schedule. The safety and tolerability of all subjects should be closely monitored during all treatment courses. Before each drug administration, subjects should undergo toxicity evaluation; treatment with the study drug can only be started after clinical evaluation and research center examination results are deemed acceptable.

The following provides a detailed description of the evaluation content.

**6.1** **Description of Evaluation Content**

**6.1.1 Demographic Information and Medical History**

Demographic information collection: initials, gender, ethnicity, marital status, date of birth, height, weight, and calculation of body surface area and body mass index based on these data; medical history: past medical history and disease treatment history, including tumor history (including all previous tumor treatment methods and procedures) and all drugs taken by the subject during the screening period (such as prescription drugs, non-prescription drugs, natural drugs or homeopathic treatments, nutritional supplements), smoking and drinking history, drug allergy history (drug name, allergic symptoms), and other comorbidities and treatment history (disease name, name, dosage, and usage of combined medications).

**6.1.2 Physical Examination**

Physical examination items include evaluation of the head, eyes, ears, nose, throat, skin, skeletal muscles, respiratory organs, cardiovascular system, gastrointestinal tract, urogenital system, and nervous system. Abnormalities at baseline should be recorded in the comprehensive medical history and baseline CRF. Clinically significant abnormalities should be recorded in the adverse event CRF. One examination each during the screening period and before surgery (if not performed within the previous 7 days).

**6.1.3 Vital Signs and Physical Activity Score**

Vital signs include respiratory rate, pulse, systolic and diastolic blood pressure (subject in a seated position), and body temperature. Vital sign results should be obtained and evaluated before each research treatment administration. One examination each during the screening period, the first day of each neoadjuvant treatment course, and before surgery (if not performed within the previous 7 days). Please refer to Attachment II for ECOG physical activity scoring according to the research flowchart.

**6.1.4 Clinical Evaluation of Tumors and Evaluation of Tumor Pathological Response**

The baseline breast tumor of subjects must have a diameter of ≥2cm. Baseline tumor evaluation will include clinical breast examination (CBE) (including breast/axilla/supraclavicular fossa), mammography, and ultrasound, as well as breast magnetic resonance imaging (MRI) scans. According to the recommendations of the Chinese Society of Clinical Oncology (CSCO) Breast Cancer Guidelines, CBE and ultrasound evaluations should be performed before each cycle of neoadjuvant therapy, and MRI evaluations should be performed every 2 cycles.

Before neoadjuvant therapy, tumor lesions must be physically marked using the research center's standard clinical operating procedures (such as skin tattoos or surgical metal clips) to allow for proper resection in case of complete tumor regression before surgery (see Attachments V and VI for more details).

Baseline axillary lymph node status should be clinically evaluated and detailed findings recorded based on the clinical practice of each research center. For subjects with clinically and/or ultrasonographically suspected axillary lymph node metastasis at baseline, axillary staging should include fine-needle aspiration (FNA) or core needle biopsy before neoadjuvant therapy.

Surgical management of axillary lymph nodes includes sentinel lymph node biopsy (SLNB) before and after neoadjuvant therapy and level I and II axillary lymph node dissection (ALND) during breast surgery. The choice of axillary surgery depends on the clinical status of the axilla, T stage, and the clinical practice treatment principles of the research center.

According to the treatment principles of each research center, tumor imaging evaluations such as MRI, CT scans, and ultrasound should be performed during the screening period and before surgery. Imaging evaluations will be assessed according to the principles of the RECIST 1.1 version. A bone scan must be performed at baseline. In the absence of radioactive isotopes, MRI scans (with gadolinium imaging if needed) are a suitable method to evaluate bone metastases.

After neoadjuvant therapy (before surgery), subjects must undergo CBE, mammography, ultrasound, and breast MRI scans.

During neoadjuvant therapy, when suspicion of other lesions arises in addition to the primary site, additional necessary imaging examinations may be performed and evaluated according to the RECIST 1.1 criteria (see Attachment III). If the lesion progresses significantly, the subject should discontinue study treatment and receive standard therapy at the research center. If contralateral ductal carcinoma in situ is found during cycles 1-6, it is not considered progressive disease (PD). However, contralateral invasive breast cancer is considered PD.

**6.1.5 Laboratory Tests**

The research samples of subjects are sent to the laboratories of various research centers for testing:

Blood routine test: including hemoglobin, red blood cell count, platelet count, white blood cell count, and neutrophil count. Hematological evaluations should be conducted during the screening period, before (or within 3 days before) the first day of each new adjuvant treatment course, and before surgery.

Blood biochemistry test: including sodium, potassium, chloride, calcium, magnesium, cholesterol, triglycerides, glucose, BUN or urea, creatinine, total protein, albumin, alkaline phosphatase, ALT, AST, GGT, total bilirubin, and direct bilirubin, etc. Blood biochemistry evaluations should be conducted during the screening period, before (or within 3 days before) the first day of each new adjuvant treatment course, and before surgery.

Coagulation function test should be completed during the screening period.

Pregnancy Test: Within 7 days before randomization, women of childbearing age in the screening period need to undergo blood HCG testing to exclude pregnancy; screening period and before surgery, each should be checked once (see the research flow chart).

Urine routine test: including urine protein, urine sugar, and urine occult blood. It should be checked once during the screening period and before surgery; if the urine routine test shows urine protein ++ or above, please conduct a 24-hour urine protein quantitative test.

Cell-free DNA (cf-DNA): Collect the plasma cell-free DNA test results of the above-mentioned case samples (collection time: screening period, after the 2nd, 4th, and 6th cycles of neoadjuvant chemotherapy, after surgery, and after recurrence and metastasis).

The following laboratory test samples will be analyzed and tested by the pathological laboratories of each research center:

During the screening of subjects, IHC and ISH will be used to determine the HER2 status of the collected tumor tissue samples. If the IHC score of >10% immune response cells is 3+ or the ISH result before randomization shows HER2 gene amplification (the ratio of HER2 gene signal to centromere 17 signal is ≥2.0 or the copy number of HER2 gene is ≥6), the HER2 status is considered positive. Tumor tissue samples obtained before surgery and resected during surgery are used to evaluate pCR.

**6.1.6 Tumor Pathological Remission Evaluation**

Pathological remission (pCR) will be evaluated through tumor tissue biopsy specimens obtained during surgery. All remission evaluations will be assessed by each research center according to the key points in Attachments 5 and 6.

**6.1.7 Diagnosis of Recurrence or Relapse**

Recurrent diseases include local, regional, and distant recurrences as well as contralateral breast cancer. Subjects diagnosed with breast in situ disease or second (non-breast) malignant tumors should be maintained through regular follow-up to fully grasp possible subsequent recurrent disease events. If there is diagnostic uncertainty (for example, there is an unclear palpable mass within the area of breast radiotherapy), histological or cytological confirmation of recurrence should be obtained as much as possible. Some subjects may suspect recurrence but die rapidly before confirmation. Autopsy reports should be obtained for such subjects as much as possible. When collecting data, the earliest diagnostic date of recurrent disease should be adopted and recorded. The diagnostic date should be based on clinical, imaging, histological, or cytological evidence. The date of disease recurrence should be based on the first diagnostic date of the lesion (i.e., objective findings) rather than the date of initial symptoms. Report all second primary malignancies that occur during the study: Subjects diagnosed with second primary malignancies but requiring no systemic treatment (i.e., chemotherapy, hormone therapy, targeted therapy, etc.) and without signs of breast cancer recurrence may continue to participate in the study and receive study drug treatment according to the study protocol and assessment schedule, as long as the investigator believes that continuing treatment is in the best interests of the subject.

The following events are not considered recurrent diseases but must be recorded truthfully:

• Lobular carcinoma in situ (LCIS) on the same side and contralateral side

• Ductal carcinoma in situ (DCIS) on the same side and contralateral side

• Cervical carcinoma in situ

• Serious adverse events related to skin basal or squamous cell carcinoma and non-breast second primary malignancies (may be reported as serious adverse events)

Should also be reported until the end of the study.

**6.1.8 Unscheduled Visit Assessment**

The investigator should conduct unscheduled visit assessments based on clinical indications.

**6.2** **Research Evaluation Timeline**

**6.2.1 Screening and Evaluation Timeline**

Before conducting any specific screening or evaluation procedures for the study, informed consent from the patient must be obtained for participation in the research. The informed consent forms of both selected and non-selected subjects will be kept at the research center. Screening examinations and evaluations will be conducted within 28 days before the first cycle of neoadjuvant therapy and within 7 days before randomization. Blood routine, blood biochemistry, cf-DNA, and other evaluations conducted at the research center before the start of the first cycle of neoadjuvant therapy must be completed within 7 days before randomization. The following examinations performed within 28 days before enrollment but before obtaining informed consent do not need to be repeated: bone scan (MRI scan), mammography and ultrasound, breast MRI, chest X-ray, ECG, ECHO, etc. All screening evaluations must be completed and reviewed to ensure that subjects meet all inclusion criteria before randomization. The researcher will keep a screening record sheet to document the detailed process of all screened subjects, including confirmation of eligibility or reasons for ineligibility, if appropriate.

For the screening evaluation items and timeline, please refer to the study flowchart.

**6.2.2 Evaluation During Treatment**

During the study visits, a 3-week (21-day) cycle will be used as the unit of treatment, with the first cycle starting on Day 1. Except for the first cycle, all evaluations should be conducted within 3 days of the scheduled visit time. Evaluations scheduled on the day of treatment should be completed before the start of treatment. If the implementation time required by the study protocol falls on a holiday or weekend, it will be postponed to the nearest available date. Except for the first cycle, blood routine and blood biochemistry evaluations must be conducted at the research center on the first day of all cycles within 3 days before treatment administration. cf-DNA evaluations will be performed after the second, fourth, and sixth cycles of neoadjuvant chemotherapy, after surgery, and after recurrence or metastasis. These laboratory evaluation results must be reviewed and recorded before treatment administration.

For the evaluation items and timeline during the treatment period, please refer to the study flowchart.

**6.2.3 Evaluation at Completion/Termination of Treatment**

Subjects will receive study drug treatment until disease progression (during neoadjuvant therapy), completion of surgical treatment, or intolerable toxicity is assessed by the researcher. Subjects required to discontinue study treatment must return to the research center for a termination visit within 28 (+7) days after the last administration of the study drug. The evaluation items and timeline for the completion/termination visit are specified in the study flowchart and accompanying notes.

**6.2.4 Follow-up Evaluation**

After completion of surgical treatment, subjects will be followed up for survival indicators and adverse events. Subjects will be followed up every 3 months (±28 days) in the first year and every 6 months thereafter until disease progression or recurrence or until 3 years after the last subject is enrolled (whichever occurs first). The follow-up evaluation items and timeline are specified in the study flowchart.

**6.3** **Subject Withdrawal, Treatment Discontinuation, and Termination of the Study and Research Center**

**6.3.1 Subject Withdrawal**

The researcher has the right to discontinue a subject's participation in the study treatment or withdraw the subject from the study at any time. Additionally, the subject also has the right to voluntarily discontinue the study treatment or withdraw from the study at any time for any reason. Reasons for discontinuing study treatment or withdrawing from the study include but are not limited to the following:

-The subject may withdraw their informed consent at any time.

-The researcher or sponsor believes that continuing the study may pose a risk to the subject's safety due to any disease.

-The researcher or sponsor believes that this decision will allow the subject to obtain the best possible benefit.

**6.3.2 Treatment Discontinuation**

Treatment must be discontinued if the subject withdraws their informed consent or in the following situations:

-Pregnancy

-Disease progression during neoadjuvant therapy

-Symptomatic LVSD (heart failure) at any point during the study

-Three intermittent delays in drug administration due to asymptomatic LVEF reduction

-Discontinuation of study treatment for more than two cycles

-Intermittent, non-tumor-related illnesses that interfere with the continuation of protocol treatment or follow-up

-Major protocol violations determined by the sponsor that may pose a risk to the subject's safety

-Multiple instances of non-compliance with protocol requirements

-Subjects who discontinue treatment prematurely should be required to return to the research center for a completion/termination visit and undergo follow-up evaluations. The primary reasons for premature discontinuation of treatment should be recorded in the corresponding CRF.

**6.3.3 Withdrawal from the Study**

For subjects who withdraw from the study, efforts should be made to obtain more detailed information about them. The main reasons for withdrawal should be recorded in the corresponding CRF. After the subject withdraws the informed consent, no follow-up for any reason is required.

**6.3.4 Study and Research Center Termination**

The sponsor has the right to terminate the study at any time. Reasons for terminating the study include but are not limited to the following:

-The incidence or severity of adverse events in this study indicates possible harm to the subjects' health.

-Subjects do not meet the inclusion criteria. If the study is suspended or the sponsor decides to terminate the study or development project, the investigator will be notified. The sponsor has the right to replace the research center at any time. Reasons for replacing the research center include, but are not limited to:

-Extremely slow recruitment progress

-Poor compliance with the trial protocol

-Inaccurate or incomplete data recording

-Failure to comply with the ICH Guidelines for Good Clinical Practice in Drug Clinical Trials

**7** **Safety Evaluation**

During the trial, the safety of the compound is evaluated through adverse event records (including serious adverse events), laboratory tests, vital signs, physical examinations, ECOG scores, echocardiography, and electrocardiogram recordings.

Subjects' symptoms and signs after medication should be closely observed during the trial. Adverse events/reactions that occur should be promptly and effectively managed to ensure the safety and interests of the subjects. After timely and effective treatment of drug-related adverse events/reactions, their types, symptoms, occurrence time, severity (or grade), symptomatic treatment methods, and outcomes should be recorded. Then, the adverse events should be analyzed, evaluated, and statistically processed to serve as a basis for the continuation of the trial.

**7.1 Physical Examination and Vital Signs**

The physical examination is conducted by the research physician and includes an assessment of the general condition, skin and mucosa, lymph nodes, head and neck, chest, abdomen, musculoskeletal system, neurological reflexes, respiratory system, cardiovascular system, urogenital system, and mental status. Body weight should be measured during each physical examination, but height only needs to be measured once during the screening period.

Vital signs include the following: body temperature, blood pressure, respiratory rate, and pulse.

The ECOG score is assessed by the research physician according to the criteria outlined in Appendix II, "Performance Status Scale (ECOG)."

**7.2** **Laboratory Tests**

Laboratory test samples will be collected at the time points specified in the "Study Flowchart." The following laboratory parameters will be sampled and tested by the respective research centers. For the safety of subjects, unscheduled clinical laboratory tests may be performed at any time.

**Table 4 - Requirements for the Content of Laboratory Tests**

| **Hematology** | **Blood Chemistry** | **Urinalysisa** |
| --- | --- | --- |
| Hemoglobin  Red Blood Cells  White Blood Cells  Neutrophil Count  Platelet Count | Total Bilirubin  Direct Bilirubin  ALT (Alanine Aminotransferase)  AST (Aspartate Aminotransferase)  Alkaline Phosphatase  GGT (Gamma-Glutamyl Transferase)  Total Protein  Albumin  Urea Nitrogen  Creatinine  Blood Glucose  Triglycerides  Cholesterol  Potassium  Sodium  Chloride  Calcium  Magnesium | Protein in Urine  Glucose in Urine  Occult Blood in Urine |
| **Infectious Disease Screening** | **Others** |  |
| Hepatitis B Five-Item Test  HIV Antibody  HCV Antibody | Pregnancy Test b |  |

Note: a. If the semi-quantitative method indicates protein ≥ 2+ (e.g., urine dipstick), a 24-hour urine protein quantitation test will be performed.

b. Female subjects of childbearing age need to undergo a serum HCG test during the screening period to exclude pregnancy, while urine HCG tests can be performed at other time points.

**7.3** **Electrocardiogram (ECG)**

A 12-lead ECG will be conducted by a qualified physician according to the time points specified in the "Study Flowchart." All ECGs are required to be performed after the subject has rested in a quiet supine position for at least 10 minutes. The ECG should include at least the following parameters: heart rate, QT, QTc, and P-R interval. Three ECGs will be performed during the screening period, and the average of the three QTcF values will be used as the baseline QTcF. For safety assessment, the research physician will compare the ECG results with the baseline values. If QTcF increases by >30msec compared to baseline or if QTcF has an absolute value of ≥470msec in any of the specified ECG measurements, two additional ECGs will be performed, with a minimum interval of 10 minutes, to confirm the accuracy of the original measurement and exclude abnormal ECGs due to incorrect lead placement. If the machine-read QTc value is prolonged, but the qualified physician determines that the QTc value is within an acceptable range, repeated measurements may be waived.

**7.4** **Echocardiography**

Echocardiography will be performed by a qualified physician according to the time points specified in the "Study Flowchart." During the administration of the trial drug, the research physician will assess and monitor LVEF at the time points specified in the protocol. If the subject develops symptoms of heart failure or a clinically significant decrease in LVEF, as judged by the investigator, they should be treated and monitored according to standard medical guidelines (see Figure 3 for details), and if necessary, a cardiologist should be consulted. If there are clinically uncontrollable severe symptoms of heart failure (NYHA Class III or IV) or a significant decrease in LVEF (below the lower limit of normal or below 50%), trial drug treatment should be discontinued according to the principles for handling adverse reactions, and the subject should continue to be treated and monitored according to standard medical guidelines.

**7.5** **Adverse Events (AEs)**

**7.5.1 Definition of Adverse Events**

An adverse event (AE) is any untoward medical occurrence in a subject administered a pharmaceutical product and which does not necessarily have a causal relationship with this treatment. For this trial, AEs will be collected from the time of signing the informed consent form until 28 days after the last administration of the study drug. Adverse events can be any unexpected, undesirable signs, symptoms, illnesses, or abnormal test results, regardless of whether they are related to the study drug. Adverse events include the following situations: 1) medical conditions/diseases that exist before the start of study treatment and are only considered adverse events if they worsen after starting the study drug; 2) any new adverse events; 3) abnormal laboratory test values or results that are considered clinically significant to constitute an adverse event.

**7.5.2 Criteria for Severity of Adverse Events**

Severity of adverse events will be assessed using the NCI-CTC AE 5.0 grading scale.

For adverse events not listed in the NCI-CTC AE 5.0 scale, the following criteria can be referenced:

Grade I: Mild; asymptomatic or mild symptoms only; detected clinically or diagnostically; no treatment required.

Grade II: Moderate; requiring minimal, local, or noninvasive treatment; limitation of instrumental activities of daily living (ADLs) that are appropriate for the subject's age. Instrumental ADLs refer to activities such as cooking, shopping, making phone calls, managing finances, etc.

Grade III: Severe or medically significant but not immediately life-threatening; resulting in hospitalization or prolongation of hospital stay; leading to disability; limitation of self-care ADLs. Self-care ADLs refer to activities such as bathing, dressing, undressing, eating, toileting, taking medication, etc., without being bedridden.

Grade IV: Life-threatening; requiring urgent intervention.

Grade V: Death related to the adverse event.

**7.5.3 Criteria for Determining the Relationship between Adverse Events and Trial Drugs**

Adverse events include all unexpected clinical manifestations that occur after the signing of the informed consent form, regardless of whether they are related to the trial drugs, whether the subjects are assigned to the trial drug group, or even whether the drugs are administered. All such events should be reported as adverse events. Any subjective discomfort or objective abnormal changes in laboratory test indicators reported by the subjects during treatment should be recorded truthfully, with the severity, duration, treatment measures, and outcome of the adverse event indicated. Clinical physicians should also comprehensively determine the relationship between adverse events and trial drugs and assess the possible association between adverse events and trial drugs according to a five-level classification method: "definitely related," "possibly related," "possibly not related," "definitely not related," and "unable to determine." "Definitely related," "possibly related," and "unable to determine" are all classified as drug adverse reactions. When calculating the incidence of adverse events, the sum of these three categories is used as the numerator, and the total number of subjects evaluated for safety is used as the denominator. The determination criteria are as follows in Table 5:

**Table 5: Criteria for Determining the Relationship between Adverse Events and Trial Drugs**

| Grade | Criteria for Determination |
| --- | --- |
| Definitely  Related | The occurrence of the event follows a reasonable time sequence after drug administration,  and the event corresponds to the known reaction types of the suspected drug. The event  improves after drug discontinuation, and the event recurs when the drug is re-administered. |
| Possibly  Related | The occurrence of the event follows a reasonable time sequence after drug administration,  but the event does not correspond to the known reaction types of the suspected drug. The  clinical status of the patient or other treatment modalities may also produce the event. |
| Possibly  Not Related | The occurrence of the event does not follow a reasonable time sequence after drug  administration, and the event does not correspond to the known reaction types of the  suspected drug. The clinical status of the patient or other treatment modalities may  produce the event. |
| Definitely  Not Related | The occurrence of the event does not follow a reasonable time sequence after drug  administration, and the event does not correspond to the known reaction types of the  suspected drug. The event resolves after the disease improves or other treatment  modalities are stopped, and the event recurs when other treatment modalities are re-used. |
| Unable to  Evaluate | The occurrence of the event has no clear relationship with the time sequence after  drug administration, but it is similar to the known reaction types of the drug. Other drugs  used concurrently may also cause corresponding events. |

**7.5.4 Recording and Reporting of Adverse Events**

Researchers shall record in detail any adverse events that occur in subjects, including descriptions of the adverse event and all related symptoms, occurrence time, severity, duration, measures taken, and final outcome (disappearance, alleviation, persistence, etc.). The assessment of drug safety in this trial begins on the day the subject signs the informed consent form and continues until 28 days after the last administration of the study treatment drug. All adverse events, whether serious or non-serious, shall be recorded in the adverse event reporting section of the case report form, and the adverse events shall be reported using accurate medical terminology. Follow-up of AEs shall be conducted according to the following requirements:

Related AEs: Follow-up shall continue until any of the following occurs:

1. Disappearance or improvement to baseline level;
2. Re-assessment determines that the event is unrelated to the trial drug;
3. Death;
4. Initiation of a new anti-tumor treatment regimen;
5. Confirmation by the investigator that no further improvement is expected;
6. Discontinuation of clinical data collection or finalization of the database.

Unrelated AEs: Follow-up shall continue until any of the following occurs:

1. Disappearance or improvement to baseline level;
2. Severity improves to within Grade 1;
3. Death;
4. Initiation of a new anti-tumor treatment regimen;
5. Confirmation by the investigator that no further improvement is expected;

6) Discontinuation of clinical data collection or finalization of the database.

**7.6 Serious Adverse Events (SAE)**

**7.6.1 Definition of Serious Adverse Events**

Serious adverse events (SAE) refer to medical events that occur during clinical trials and require hospitalization or extension of hospital stay, disability, impairment of work ability, life-threatening conditions, or death, leading to congenital malformations, etc. An adverse event that meets one or more of the following criteria is considered an SAE:

-Leads to death;

-Life-threatening (defined as the subject being in imminent danger of death at the time of the event);

-Requires hospitalization or extension of hospital stay;

-Leads to permanent or severe disability/loss of function;

-Leads to congenital abnormalities or birth defects;

-A significant medical event: these adverse events may not lead to death, be life-threatening, or require hospitalization, but based on medical judgment, the event may harm the subject and require medical or surgical intervention to prevent any of the above outcomes.

Researchers shall promptly report all SAEs, including clinical diagnosis, treatment, and outcome, and follow up until the condition returns to normal, alleviates to Grade 1 or baseline level, or stabilizes. Detailed records shall be made in the original medical records and CRF tables (including adverse event reporting forms, and in the case of death, a death record form shall be completed), and an SAE report form shall be submitted.

**7.6.2 Disease Progression**

Disease progression is defined as the deterioration of a subject's condition due to the indication under study. It includes both radiological progression and progression in clinical symptoms and signs. The appearance of new metastases relative to the primary tumor or the progression of existing metastases is considered as disease progression. Events that are life-threatening, require hospitalization or extension of hospital stay, or lead to permanent or severe disability/impairment/impairment of work ability, congenital abnormalities, or birth defects due to symptoms and signs of disease progression are not reported as SAEs. However, death due to symptoms and signs of disease progression is reported as an SAE.

**7.6.3 Hospitalization**

Adverse events in clinical studies that lead to hospitalization or prolongation of hospital stay should be considered SAEs. Any initial admission to a medical facility, even if it is less than 24 hours, meets this criterion.

The following hospitalizations do not constitute SAEs:

-Rehabilitation facilities

-Nursing homes

-Routine emergency room admissions

-Day surgeries (e.g., outpatient/day/ambulatory surgeries)

-Hospitalization or prolongation of hospital stay unrelated to adverse events is not an SAE in itself. For example:

--Admission due to pre-existing diseases without the occurrence of new adverse events or worsening of pre-existing diseases (e.g., for checking persistent laboratory abnormalities that have been present since before the trial);

--Hospitalization for administrative reasons (e.g., annual routine physical examination);

Hospitalization specified in the trial protocol during the clinical trial (e.g., procedures required by the trial protocol);

--Elective hospitalization unrelated to adverse events (e.g., elective plastic surgery);

Scheduled treatment or surgical procedures should be documented in the entire trial protocol and/or the subject's individual baseline data;

--Admission solely for blood product use. Diagnostic or therapeutic invasive (e.g., surgical) and non-invasive procedures should not be reported as adverse events.

However, the disease condition leading to such procedures should be reported if it meets the definition of an adverse event, such as acute appendicitis that occurs during the adverse event reporting period should be reported as an adverse event, and the appendectomy performed due to it should be recorded as the treatment method for that adverse event.

**7.6.4 Potential Drug-induced Liver Injury**

If abnormal AST and/or ALT levels are accompanied by abnormally elevated total bilirubin levels, and there are no other causes of liver injury, it will be considered as potential drug-induced liver injury. Such cases should always be considered as significant medical events and reported as SAEs.

The definition of potential drug-induced liver injury is as follows:

| Baseline | Normal (AST/ALT and Total Bilirubin) | Abnormal (AST/ALT and Total Bilirubin) |
| --- | --- | --- |
| Treatment Period | ALT or AST ≥ 3×ULN  With total bilirubin ≥ 2×ULN  Alkaline phosphatase ≤ 2×ULN  No hemolysis | AST or ALT ≥ 2×baseline level, and  value ≥ 3×ULN; or AST or ALT ≥ 8×ULN  With total bilirubin increase ≥ 1×ULN or  value ≥ 3×ULN |

Subjects should return to the research center for evaluation as soon as possible (preferably within 48 hours) after being informed of abnormal results. The evaluation should include laboratory tests, detailed medical history inquiry, and physical examination, and should consider the possibility of liver tumors (primary or secondary).

In addition to repeat testing of AST and ALT, the laboratory tests to be performed should also include albumin, creatine kinase, total bilirubin, direct and indirect bilirubin, gamma-glutamyl transferase, prothrombin time (PT)/international normalized ratio (INR), and alkaline phosphatase. Detailed medical history collection should include: alcohol consumption history, acetaminophen use, soft drugs, various supplements, family history, occupational exposure, sexual history, travel history, contact history with jaundice patients, surgery, blood transfusion, history of liver disease, or allergic diseases. Further testing may also include testing for acute hepatitis A, B, C, and E, and liver imaging studies (e.g., biliary tract). If repeated testing still confirms compliance with the above-mentioned laboratory criteria, and there are no other causes for abnormal liver function tests, the possibility of potential drug-induced liver injury should be considered without waiting for all liver function etiology test results. Such cases of potential drug-induced liver injury should be reported as SAEs.

**7.6.5 Reporting System for Serious Adverse Events (SAEs)**

The reporting of SAEs should start from the time the subject signs the informed consent form until 28 calendar days (inclusive) after the last use of the study drug. During the trial, if an SAE occurs, whether it is an initial report or a follow-up report, the investigator must immediately fill out the "Serious Adverse Event (SAE) Report Form," sign and date it, and report it to the sponsor within 24 hours of the investigator's knowledge of the SAE, and report to relevant units according to regulatory requirements. SAEs should be recorded in detail with symptoms, severity, occurrence time, treatment time, measures taken, concomitant medications, follow-up time and method, and outcome. If the investigator believes that the SAE is unrelated to the trial drug but potentially related to the study conditions (e.g., discontinuation of the original treatment or complications during the trial), this relationship should be described in detail in the narrative section of the SAE page of the case report form. If the intensity of the SAE or its relationship with the test drug changes, an SAE follow-up report should be immediately sent to the sponsor.

**7.6.6 Follow-up of AE/SAE**

All AE/SAEs should be followed up until they disappear, return to baseline levels or ≤ Grade 1, reach a stable state, or receive a reasonable explanation (e.g., loss of follow-up, death).

The investigator should inquire about AE/SAEs that occurred after the previous visit during each visit and provide follow-up information promptly upon request from the sponsor.

**7.7 Pregnancy**

If a female subject becomes pregnant during the clinical trial, the subject should immediately discontinue the study drug treatment, and the investigator should report it to the sponsor within 24 hours of learning about the pregnancy.

The investigator should follow up on the pregnancy outcome until one month after the mother's delivery and report the results to the sponsor. If the pregnancy outcome is stillbirth, spontaneous abortion, or fetal malformation, it will be considered an SAE and needs to be reported according to the SAE timeline and requirements. If the subject experiences an SAE concurrently during pregnancy, the "CFDA Serious Adverse Event Report Form" must also be filled out, and the SAE reporting procedure must be followed for reporting.

**8** **Study Management**

**8.1 Ethical Norms and Informed Consent**

**8.1.1 Ethical Norms**

This clinical trial must adhere to the Declaration of Helsinki (2008 edition), the Good Clinical Practice (GCP) regulations issued by the CFDA, and relevant laws and regulations. Before the commencement of the trial, approval from the ethics committee of the responsible institution must be obtained. During the clinical research period, any modifications made to the trial protocol must be reported to and filed with the ethics committee. Researchers are responsible for submitting interim reports to the ethics committee periodically based on their requirements, and notifying the ethics committee of the completion of the trial upon its conclusion.

**8.1.2 Informed Consent**

Before receiving the trial medication, subjects must provide informed consent to participate in this trial to safeguard their legitimate rights and interests. Researchers are obligated to provide subjects or their designated representatives with a complete and comprehensive introduction to the purpose of the study, the role of the medication, possible adverse reactions and risks, and to inform them of their rights, risks, and benefits. The conversation is a crucial part of the informed consent process. If the subject and their legal representative are illiterate, the informed consent process should be witnessed, with the subject or their legal representative giving verbal consent and signing the informed consent form, with the witness's signature on the same day as the subject's signature. The informed consent form should indicate the version number and version date.

**8.2** **Trial Drug Management**

The management, dispensing, and recall of clinical drugs in this trial shall be handled by dedicated personnel. Researchers must ensure that all trial drugs are only used for subjects participating in this clinical trial, and their dosage and usage should follow the trial protocol. Monitors are responsible for overseeing the supply, use, storage, and disposal of remaining drugs in clinical trials.

**8.3 Protocol Revision**

Any necessary changes to the protocol must be made in the form of protocol revisions, which require the signature and approval of the sponsor and the principal investigator before being submitted to the ethics committee for approval or filing. Detailed information on all previous modifications must be included in the protocol.

**8.4** **Quality Control and Assurance**

-The clinical research unit must be a drug clinical research base with clinical research conditions determined by the CFDA.

-Researchers must be medically trained physicians with clinical trial training and work under the guidance of senior professionals.

-Clinical wards must be inspected before the trial to ensure they meet standardized requirements and have complete rescue equipment.

-Professional nursing staff should administer medication to subjects, thoroughly understand the medication intake, and ensure subjects' compliance.

-All research centers must strictly follow the research protocol and truthfully fill out the Case Report Form (CRF).

--Monitors should follow standard operating procedures to oversee the conduct of clinical trials, confirm that all data records and reports are accurate and complete, all CRFs are filled out correctly, and are consistent with the original data, ensuring that the trial is conducted in accordance with the clinical research protocol.

--Research centers participating in the trial should accept inspections by the sponsor and drug regulatory authorities, and it is particularly important that researchers and their relevant personnel provide convenience and time for inspections and audits.

**9** **Data Management**

**9.1 Data Recording**

**9.1.1 Original Medical Records:** The original medical records, as the original documents of clinical trials, should be kept intact. They are filled in and kept by researchers. The original medical records can be outpatient or inpatient medical records of the research center, and the writing should be neat and easy to read, facilitating data verification between the monitors and the CRF during each inspection. The medical records should include detailed records of subjects' demographic information, medical history and medication history, vital signs, physical examination, clinical indicators, adverse reactions and their management, and concomitant medication.

**9.1.2 CRF Completion:** The data in the CRF should be derived from original documents such as original medical records and laboratory reports, and should be consistent with the original documents. Any observations and examination results in the trial should be recorded in the CRF promptly, accurately, completely, clearly, and truthfully. No changes should be made arbitrarily. All items in the CRF must be filled in, and no items should be left blank or omitted. If necessary, when making corrections to the CRF data, the reason for the change must be recorded according to requirements.

**9.2** **Data Management**

**9.2.1 Database Establishment:** The data administrator establishes the research data collection system and database according to the research protocol and provides it for online use before the subjects' enrollment.

**9.2.2 Data Entry and Verification:** After the researchers fill in and submit the CRF, monitors, data administrators, and medical personnel should review the relevant data one by one. Any issues found during the review should be questioned, requiring the researchers to respond. After the cleanup is completed, the CRF needs to be signed and confirmed by the researchers.

**9.2.3 Data Archiving:** After the completion of the study, the system will generate electronic data and archive them, which will be submitted to the sponsor and various institutions for retention, ready for inspection.

The preservation and management of trial data must be conducted in accordance with regulations. Researchers should notify the sponsor before destroying any documents or materials related to the trial.

**9.3 Protocol Deviations**

All requirements stipulated in the research protocol must be strictly enforced. Any intentional or unintentional deviation or violation of the trial protocol and GCP principles can be classified as a deviation or violation of the protocol. If the monitors find deviations in the monitoring process, the researchers or monitors should fill out a protocol violation record, detailing the time of discovery, the time and process of the event, the cause, and corresponding handling measures. The researchers should sign the record and notify the ethics committee and the sponsor.

**9.4 Data Preservation**

To ensure the evaluation and supervision of clinical research by the National Medical Products Administration and the sponsor, researchers should agree to preserve all research materials, including the original records of subjects' hospitalization, informed consent forms, case report forms, detailed records of drug distribution, etc. Researchers should notify the sponsor before destroying any documents or materials related to the trial.

**10 Data Analysis and Statistical Methods**

**10.1 Sample Size Estimation**

This study is designed with a particular focus on whether pyrotinib could improve the pCR rate in patients with primary resistance to trastuzumab. As there were no specific published data on the pCR rate in patients without an early response to trastuzumab, the pCR rates were expected to be 12% for cohort B) and 35% for cohort C based on real-world observations. Using an estimation design with 1:2 enrollment ratio, α=0.05 (one-sided and β=0.20, the sample sizes for cohorts B and C were then determined to be 32 and 63 patients, respectively.

**10.2** **Statistical Analysis**

Efficacy was assessed in the intention-to-treat population, which included all patients enrolled in this study. Safety was assessed in the safety analysis set, which included all patients who received at least one dose of study drug. The primary endpoint was evaluated for each cohort, and the 95% confidence interval (CI) was calculated using the Wilson procedure without correction for continuity. Multivariate logistic regression analysis will be employed to determine independent factors related to pCR. All statistical analyses will be performed using SPSS (version 22.0). A P value of less than 0.05 will be considered statistically significant.

**11** **Research Schedule**

Estimated first subject enrollment time: Q3 2020

Estimated last subject enrollment time: Q3 2022

**12. References**

[1]. Torra LA, et al. GLOBOCAN 2012. Global cancer statistics. CA Cancer J Clin. 2015

[2]. Wanqing CHEN ,Rongshou ZHENG.Incidence,mortality and survival analysis of breast cancer in China.Chinese Journal of Clinical Oncology, 2015,42(13):668-674

[3]. Slamon DJ, et al. Human breast cancer：correlation of relapse and survival with amplification of the HER-2/neu oncogene. Science.1987, 235:177-82

[4]. Ross JS, et al. Breast cancer biomarker and HER2 testing after 10 years of anti-HER2 therapy. Drug News Perspect. 2009,22:93-106

[5]. Freudenberg JA, Wang Q, Katsumata M, Drebin J, Nagatomo I, Greene MI. [The role](https://www.ncbi.nlm.nih.gov/pubmed/19450579) [of HER2 in early breast cancer metastasis and the origins of resistance to HER2-targeted](https://www.ncbi.nlm.nih.gov/pubmed/19450579) [therapies.](https://www.ncbi.nlm.nih.gov/pubmed/19450579) Exp Mol Pathol. 2009 Aug;87(1):1-11

[6]. Xiao Hong, et al. A prospective multicenter study of HER2/neu status in human breast cancer patients of mainland China:comparison of fluorescence in situ hybridization and immunohistochemistry. China J Lab Med 2010,33:655

[7].《Expert Consensus on Clinical Diagnosis and Treatment of Human Epidermal Growth Factor Receptor 2 (HER2)-Positive Breast Cancer (2016 Edition)》

[8]. Cortazar P, Zhang L, Untch M, et al. Pathological complete response and long-term clinical benefit in breast cancer: the CTNeoBC pooled analysis. Lancet. 2014;384(9938):164-172

[9]. Killelea BK, Yang VQ, Mougalian S, et al. Neoadjuvant chemotherapy for breast cancer increases the rate of breast conservation: results from the National Cancer Database. J Am Coll Surg. 2015;220(6):1063-1069

[10]. King TA and, Morrow M. Surgical issues in patients with breast cancer receiving neoadjuvant chemotherapy. Nat Rev Clin Oncol. 2015;12(6):335-343

[11]. Kim MM, Allen P, Gonzalez-Angulo AM, et al. Pathologic complete response to neoadjuvant chemotherapy with trastuzumab predicts for improved survival in women with HER2-overexpressing breast cancer. Ann Oncol. 2013;24(8):1999-2004

[12]. Untch M, Konecny GE, Paepke S, et al. Current and future role of neoadjuvant therapy for breast cancer. Breast. 2014;23(5):526-537

[13]. Amiri-Kordestani L, Beaver JA, Cortazar P. Neoadjuvant therapy as a platform for drug development: Current controversies and regulatory perspectives. Oncology (Williston Park). 2015;29(11):843-844,846

[14]. DeMichele A, Yee D, Berry DA, et al. The neoadjuvant model is still the future for drug development in breast cancer. Clin Cancer Res. 2015;21(13):2911-2915

[15]. Dawood S, Broglio K, Buzdar AU, et al. Prognosis of women with metastatic breast cancer by HER2 status and trastuzumab treatment: an institutional-based review. J Clin Oncol. 2010;28(1):92-98

[16]. Lambertini M, Ponde NF, Solinas C, et al. Adjuvant trastuzumab: a 10-year overview of its benefit. Expert Rev Anticancer Ther. 2017;17(1):61-74

[17]. Slamon D, Eiermann W, Robert N, et al. Adjuvant trastuzumab in HER2-positive breast cancer. N Engl J Med. 2011;365(14):1273-1283

[18]. Slamon DJ, et al. Use of chemotherapy plus monoclonal antibody against HER2 for metastatic breast cancer that overexpresses HER2. NEJM, 2001

[19]. Marty M, et al. Randomized Phase II trial of the efficacy and safety of trastuzumab combined with docetaxel in patients with human epidermal growth factor receptor 2-positive metastatic breast cancer administered as first-line treatment: th M77001 Study Group. JCO, 2005

[20]. Piccart-Gebhart MJ, et al. Trastuzumab after adjuvant chemotherapy in HER2- positive breast cancer. NEJM, 2005

[21]. Romond EH,et al. Trastuzumab plus adjuvant chemotherapy for operable HER2- positive breast cancer. NEJM, 2005

[22]. Fisher ER, et al. Pathobiology of preoperative chemotherapy findings from the National Surgical Adjuvant Breast and Bowel (NSABP) protocol B-18. Cancer 2002:95(4):681-95

[23]. Alm EI-Din MA, Taghian AG. Breast conservation therapy for patients with locally advanced breast cancer. Semin Radiat Oncol, 2009.19:229-35

[24]. Mauri D, et al. Neoadjuvant versus adjuvant systemic treatment in breast cancer:

A meta-analysis. J Natl Cancer Inst 2005

[25]. Fisher B, et al. Effect of preoperative chemotherapy on the outcome of women with operable breats cancer. JCO. 1998

[26]. Kurosumi M, et al. Significance of histological evaluation in primary therapy for breast cancer recent trends in primary modality with pathological complete response (pCR) as endpoint. Breast cancer, 2004

[27]. Bear HD, et al.Sequential preoperative or postoperative docetaxel added to preoperative doxorubincin plus cyclophosphamide for operable breast cancer: National

Surgical Adjuvant Breast and Bowel Project Protocol B-27. JCO, 2006

[28]. Kaufmann M, et al. Recommendations from an international expert panel on the use of neoadjuvant (primary) systemic treatment of operable breast cancer: an update.

JCO, 2006

[29]. Gianni L, Pienkowski T, Im YH, et al. 5-year analysis of neoadjuvant pertuzumab and trastuzumab in patients with locally advanced, inflammatory, or early-stage HER2- positive breast cancer (NeoSphere): a multicentre, open-label, phase 2 randomised trial. Lancet Oncol. 2016;2045(16):163-167

[30]. Baselga J, Bradbury I, Eidtmann H, et al. Lapatinib with trastuzumab for HER2- positive early breast cancer (NeoALTTO): a randomised, open-label, multicentre, phase 3 trial. Lancet. 2012;379(9816):633-640

[31]. Untch M, Loibl S, Bischoff J, et al. Lapatinib versus trastuzumab in combination with neoadjuvant anthracycline-taxane-based chemotherapy (GeparQuinto, GBG 44): a randomised phase 3 trial. Lancet Oncol. 2012;13(2):135-144

[32]. Gianni L, Eiermann W, Semiglazov V, et al. Neoadjuvant chemotherapy with trastuzumab followed by adjuvant trastuzumab versus neoadjuvant chemotherapy alone, in patients with HER2-positive locally advanced breast cancer (the NOAH trial): a randomised controlled superiority trial with a parallel HER2-negative cohort. Lancet. 2010;375(9712):377-384

[33]. Parra-Palau JL, Pedersen K, Peg V, et al. A major role of p95/611-CTF, a carboxy- terminal fragment of HER2, in the down-modulation of the estrogen receptor in HER2- positive breast cancers. Cancer Res. 2010;70(21):8537-8546

[34]. Patricia Cortazar, Lijun Zhang, Michael Untch, et al. Pathological complete response and long term clinical benefit in breast cancer: the CTNeoBC pooled analysis. Lancet. 2014;384(9938):164-172

[35]. Von Minckwitz G, Untch M, Blohmer JU, et al. Definition and impact of pathologic complete response on prognosis after neoadjuvant chemotherapy in various intrinsic breast cancer subtypes. J Clin Oncol. 2012;30(15):1796-1804

[36]. Guarneri V,et al. Preoperative chemotherapy plus trastuzumab, lapatinib, or both in human epidermal growth factor receptor 2-positive operable breast cancer: results of the randomized phase II CHER-LOB study. J Clin Oncol, 2012

[37]. Robodoux A , et al, Lapatinib as a component of neoadjuvant therapy for HER2- positive operable breast cancer (NSABP protocol B-41): an open-label, randomised phase 3 trial. Lancet ONCO, 2013

[38]. Lisa AC, et al. Molecular Heterogeneity and Response to Neoadjuvant Human Epidermal Growth Factor Receptor 2 Targeting in CALGB 40601, a Randomized Phase III Trial of Paclitaxel Plus Trastuzumab With or Without Lapatinib. JCO, 2016

[39]. Harvey V, et al. Phase III trial comparing three doses of docetaxel for treatment of second-line advanced breast cancer. JCO, 2006

[40]. Fujiwara K, et al. Phase II dose escalation: a novel approach to balancing efficacy and toxicity of anticancer agents. JDOCSG. Anticancer Res.1999

[41]. Sato N, et al. combination docetaxel and trastuzumab treatment for patients with HER-2 overexpressing metastatic breast cancer: a multicenter, phase II study. Breast Cancer, 2006

[42]. Z.Liu, Z.Li, J.Qu, et al., Radiomics of multiparametric MRI for pretreatment prediction of pathologic complete response to neoadjuvant chemotherapy in breast cancer: a multicenter study[J], Clin. Cancer Res. 25 (12) (2019) 3538–3547.]

[43]. Perez-Garcia, J.M., et al., Chemotherapy de-escalation using an (18)F-FDG-PET-based pathological response-adapted strategy in patients with HER2-positive early breast cancer (PHERGain): a multicentre, randomised, open-label, non-comparative, phase 2 trial. Lancet Oncol, 2021. 22(6): p. 858-871]

**Attachment 1: Clinical Staging Criteria for Breast Cancer**

**(AJCC Breast Cancer TNM Staging)**

| Stage 0 | TisN0M0 |
| --- | --- |
| Stage I | T1N0M0 |
| Stage IIA | T0N1M0 |
|  | T1N1M0 |
|  | T2N0M0 |
| Stage IIB | T2N1M0 |
|  | T3N0M0 |
| Stage IIIA | T0N2M0 |
|  | T1N2M0 |
|  | T2N2M0 |
|  | T3N1、2M0 |
| Stage IIIB | T4N0M0，T3N1M0，T4N2M0 |
| Stage IIIC | Any T，N3M0 |
| Stage IV | Any T Any N，M1 |

**Attachment 2: ECOG Performance Status Scale**

**(Eastern Cooperative Oncology Group)**

| Score | Description |
| --- | --- |
| 0 | Asymptomatic, fully active, able to carry on all pre-disease performance without restriction. |
| 1 | Symptomatic, but completely ambulatory, capable of all self-care but unable to carry out any work activities. Up and about more than 50% of waking hours. |
| 2 | Symptomatic, in bed less than 50% of waking hours, capable of only limited self-care, unable to work. |
| 3 | Symptomatic, in bed more than 50% of waking hours, but not bedridden, unable to work, requires considerable assistance and attention. |
| 4 | Completely disabled, bedridden, unable to self-care, requires total care and attention. |
| 5 | Dead. |

### **Attachment 3:** **Criteria for Evaluation of Solid Tumor Response**

Criteria for Evaluation of Solid Tumor Response - Version 1.1 (Excerpt)

（New Response Evaluation Criteria in Solid Tumors: Revised RECIST Version 1.1）

Note: This attachment is internal translation material for reference only. Please refer to the English version for actual operation.

1. Background (omitted)
2. Purpose (omitted)
3. Measurability of Tumors at Baseline

3.1 Definition: At baseline, tumor lesions/lymph nodes will be classified as measurable and non-measurable according to the following definitions:

3.1.1 Measurable Lesions

Tumor lesions: At least one precisely measurable diameter (recorded as the maximum diameter) must be present, with a minimum length as follows:

-CT scan: 10mm (with slice thickness not exceeding 5mm)

-Clinical routine examination instrument: 10mm (tumors that cannot be accurately measured with a caliper should be recorded as non-measurable)

-Chest X-ray: 20mm

-Malignant lymph nodes: Pathologically enlarged and measurable, with a short-axis diameter of ≥15mm on CT scan (recommended slice thickness not exceeding 5mm). Only the short-axis diameter will be measured and followed up at baseline and subsequent visits.

3.1.2 Non-measurable Lesions

All other lesions, including small lesions (longest diameter <10mm or pathological lymph nodes with a short-axis diameter ≥10mm to <15mm) and unmeasurable lesions. Unmeasurable lesions include: meningeal disease, ascites, pleural or pericardial effusion, inflammatory breast cancer, carcinomatous lymphangitis of the skin/lungs, abdominal masses that cannot be confirmed and followed up radiographically, and cystic lesions.

3.1.3 Special Considerations for Lesion Measurement

Bone lesions, cystic lesions, and lesions that have undergone prior local treatment require special note:

Bone lesions:

-Bone scans, PET scans, or plain radiographs are not suitable for measuring bone lesions, but can be used to confirm the presence or disappearance of bone lesions.

-Osteolytic lesions or mixed osteolytic/osteoblastic lesions with a definite soft tissue component that meets the above measurability definition can be considered measurable lesions if they can be evaluated using cross-sectional imaging techniques such as CT or MRI.

-Osteoblastic lesions are non-measurable.

Cystic lesions:

-Lesions that meet the criteria for pure cysts on radiological imaging should not be considered malignant lesions solely because they are defined as simple cysts, and are neither measurable nor non-measurable.

-If the cystic lesion is a metastatic lesion and meets the above measurability definition, it can be considered a measurable lesion. However, if non-cystic lesions are present in the same patient, non-cystic lesions should be preferentially selected as target lesions.

Locally treated lesions:

Lesions located in areas that have undergone radiotherapy or other local regional treatment are generally considered non-measurable unless there is clear progression of the lesion. The study protocol should describe in detail the conditions for these lesions to be considered measurable.

3.2 Instructions for Measurement Methods

3.2.1 Lesion Measurement: All tumor measurements during clinical evaluation should be recorded in metric units. All baseline assessments of tumor lesion size should be completed as close to the start of treatment as possible and must be completed within 28 days (4 weeks) before the start of treatment.

3.2.2 Evaluation Methods

The same techniques and methods should be used for baseline assessment and subsequent measurements of lesions. Except for lesions that cannot be evaluated using imaging examinations and can only be evaluated clinically, all lesions must be evaluated using imaging examinations. Clinical lesions: Clinical lesions can only be considered measurable if they are superficial and have a diameter ≥10mm when measured (such as skin nodules). For subjects with skin lesions, it is recommended to use color photographs with rulers measuring the size of the lesions for archiving. When both imaging and clinical examinations are used to evaluate a lesion, imaging should be preferred whenever possible due to its objectivity and reproducibility for review at the end of the study.

Chest X-ray: When tumor progression is an important endpoint in the study, chest CT should be preferred as it is more sensitive than X-ray, especially for detecting new lesions. Chest X-ray is only suitable when the boundary of the measured lesion is clear and lung ventilation is good.

CT, MRI: CT is currently the best available and reproducible method for evaluating treatment efficacy. The definition of measurability in this guideline is based on CT scans with a slice thickness of ≤5 mm. If the CT slice thickness is greater than 5mm, the minimum measurable lesion should be twice the slice thickness. MRI is also acceptable in some cases (such as whole-body scanning).

Ultrasound: Ultrasound should not be used as a measurement method for evaluating lesion size. Due to its operator-dependency, ultrasound lacks reproducibility after the measurement is completed and cannot guarantee consistency in technique and measurements between different measurements. If new lesions are detected using ultrasound during the trial, CT or MRI should be used for confirmation. MRI can be used as an alternative if considering the radiation exposure of CT.

Endoscopy, laparoscopy: These techniques are not recommended for objective tumor evaluation, but they can be used to confirm CR when biopsy specimens are obtained. They can also be used to confirm recurrence in trials with CR as the endpoint, followed by recurrence or surgical resection.

Tumor Markers: Tumor markers cannot be used alone to evaluate objective tumor response. However, if the marker level exceeds the upper limit of normal at baseline, it must return to normal levels when evaluating complete response. As tumor markers vary from disease to disease, this factor needs to be considered when writing the measurement criteria into the protocol. Specific criteria for CA-125 response (recurrent ovarian cancer) and PSA (recurrent prostate cancer) response have been published. The Gynecologic Cancer InterGroup (GCIG) has also developed CA-125 progression criteria that will be added to the objective tumor response criteria for first-line ovarian cancer treatment protocols.

Cytological/histological techniques: Under specific conditions specified in the protocol, these techniques can be used to identify PR and CR (e.g., residual benign tumor tissue often present in lesions of germ cell tumors). When effusion may be a potential side effect of a certain therapy (e.g., treatment with taxane compounds or angiogenesis inhibitors), and measurable tumors meet the criteria for response or disease stability, the appearance or worsening of tumor-related effusion during treatment can be confirmed by cytological techniques to distinguish between response (or disease stability) and disease progression.

4 Evaluation of Tumor Response

4.1 Assessment of Total Tumor Burden and Measurable Lesions

To evaluate objective response or potential future progression, it is necessary to perform a baseline assessment of the total tumor burden of all tumor lesions as a reference for subsequent measurements. In clinical protocols where objective response is the primary treatment endpoint, only subjects with measurable lesions at baseline are eligible for inclusion. Measurable lesions are defined as the presence of at least one measurable lesion. For trials where disease progression (time to progression or progression at a fixed date) is the primary treatment endpoint, the protocol inclusion criteria must clearly specify whether only subjects with measurable lesions are eligible or if those without measurable lesions can also be included.

4.2 Baseline Record of Target Lesions and Non-Target Lesions

When there are more than one measurable lesion at baseline assessment, all lesions should be recorded and measured, with a total of not more than 5 (not exceeding 2 per organ), selected as target lesions to represent all involved organs (this means that subjects with only one or two cumulative organs can select a maximum of two or four target lesions for baseline measurement).

Target lesions must be selected based on size (longest diameter) and must represent all involved organs, and the measurements must have good repeatability. Sometimes, when the largest lesion cannot be repeatedly measured, a new largest lesion that can be repeatedly measured may be selected.

Lymph nodes require special attention as they are normal tissues and can be detected by imaging even without tumor metastasis. Pathological lymph nodes defined as measurable nodules or even target lesions must meet the following criteria: a short diameter ≥15mm measured by CT. Only the short diameter needs to be measured at baseline. Radiologists often use the short diameter of the nodule to determine whether there is tumor metastasis in the nodule. The nodule size is generally expressed as two-dimensional data detected by imaging (CT uses the axial plane, while MRI selects one plane from the axial, sagittal, or coronal planes). The minimum value is taken as the short diameter. For example, an abdominal nodule of 20mm x 30mm has a short diameter of 20mm, which can be considered a malignant and measurable nodule. In this example, 20mm is the measured value of the nodule. Nodules with a diameter ≥10mm but <15mm should not be considered target lesions. Nodules <10mm are not pathological nodules and do not need to be recorded or further observed.

The sum of the diameters of all target lesions (including the longest diameter of non-nodal lesions and the short diameter of nodal lesions) will be reported as the baseline sum of diameters. If lymph node diameters are included, as mentioned above, only the short diameters will be calculated. The baseline sum of diameters will serve as a reference value for the baseline level of disease.

All remaining lesions, including pathological lymph nodes, can be considered non-target lesions and do not require measurement, but should be recorded at baseline assessment. They can be recorded as "present," "absent," or in rare cases, "unequivocal progression." Widespread target lesions can be recorded together with the target organs (e.g., extensive pelvic lymph node enlargement or massive liver metastases).

4.3 Response Criteria

4.3.1 Evaluation of Target Lesions

Complete Response (CR): Disappearance of all target lesions, and the short diameter of all pathological lymph nodes (including target and non-target nodes) must be reduced to <10mm. Partial Response (PR): At least a 30% decrease in the sum of diameters of target lesions, taking as reference the baseline sum diameters. Progressive Disease (PD): An increase of at least 20% in the sum of diameters of target lesions, taking as reference the smallest sum on study (this includes the baseline sum if that is the smallest on study); in addition, there must be an absolute increase of at least 5mm (the appearance of one or more new lesions is also considered progressive disease). Stable Disease (SD): Neither sufficient shrinkage to qualify for PR nor sufficient increase to qualify for PD, taking as reference the smallest sum of diameters on study.

4.3.2 Considerations for Target Lesion Evaluation

Lymph Nodes: Even if lymph nodes identified as target lesions decrease to less than 10mm, the actual short diameter value corresponding to the baseline measurement should still be recorded each time (consistent with the anatomical plane used for baseline measurement). This means that if a lymph node is a target lesion, it cannot be considered as completely disappeared, even if it meets the criteria for CR, as the short diameter of a normal lymph node is defined as <10mm. In the CRF or other recording methods, target lymph node lesions should be specifically recorded in a designated section: for CR, all lymph node short diameters must be <10mm; for PR, SD, and PD, the actual measured short diameters of target lymph nodes will be included in the sum of target lesion diameters.

Too Small to Measure Target Lesions: In clinical studies, all lesions (nodal or non-nodal) recorded at baseline should be recorded with actual measured values during subsequent assessments, even if the lesions are very small (e.g., 2mm). However, sometimes the lesions may be too small to produce a clear image on CT scans, making it difficult for radiologists to define an exact numerical value, and they may report it as "too small to measure." In such cases, it is important to record the previous value on the CRF. If the radiologist believes that the lesion has disappeared, it should be recorded as 0mm. If the lesion is present but too blurry to give an accurate measurement, a default value of 5mm can be used. (Note: This is unlikely to occur with lymph nodes, as they usually have measurable dimensions under normal conditions or are often surrounded by fatty tissue, such as in the retroperitoneal space; however, if such a situation occurs, a default value of 5mm should also be used.) The default value of 5mm is derived from the slice thickness of CT scans (this value does not change with different slice thicknesses). Since it is unlikely for the same measurement to be repeated, providing this default value will reduce the risk of misassessment. However, it should be reiterated that if the radiologist can provide an exact numerical value for the lesion size, even if it is less than 5mm, the actual value must be recorded.

Separated or Confluent Lesions: When non-nodal lesions split into fragments, the longest diameters of the separated parts are added together to calculate the sum of lesion diameters. Similarly, for confluent lesions, they can be distinguished by the planes between the confluent parts, and the maximum diameters of each part are calculated. However, if the confluent lesions are inseparable, the longest diameter should be taken as the overall longest diameter of the fused lesion.

4.3.3 Evaluation of Non-Target Lesions

This section defines the response criteria for non-target lesions. Although some non-target lesions are actually measurable, they do not require measurement and only require qualitative assessment at specified time points as defined in the protocol. Complete Response (CR): Disappearance of all non-target lesions and normalization of tumor markers. All lymph nodes are non-pathological size (short diameter <10mm). Non-Complete Response/Non-Progressive Disease: Persistence of one or more non-target lesions and/or tumor marker levels above normal. Progressive Disease: Unequivocal progression of pre-existing non-target lesions. Note: The appearance of one or more new lesions is also considered progressive disease.

4.3.4 Special Considerations for Non-Target Lesion Progression Evaluation

For the supplementary explanation of the definition of non-target lesion progression, it is noted that when there are measurable non-target lesions in the subject, even if the target lesions are assessed as stable or partially responsive, a clear definition of progression based on non-target lesions must be made when the overall deterioration degree of non-target lesions has reached the extent that requires termination of treatment. However, general enlargement in the size of one or more non-target lesions is often insufficient to meet the progression criteria. Therefore, when the target lesions are stable or partially responsive, it is rarely the case that overall tumor progression can be defined solely based on changes in non-target lesions.

When non-target lesions in subjects are all non-measurable: In some phase III trials, this scenario arises when the inclusion criteria do not stipulate the necessity of measurable lesions. The overall assessment still refers to the above-mentioned criteria, but since there is no measurable data available for the lesions, it is not easy to evaluate the deterioration of non-target lesions. Therefore, when the change in non-target lesions leads to an increase in the overall disease burden equivalent to the progression of target lesions, an effective detection method needs to be established for evaluation. For example, when the increase in tumor burden is equivalent to an additional 73% in volume (corresponding to a 20% increase in the diameter of measurable lesions), or when peritoneal effusion changes from "minimal" to "large," lymphatic lesions spread from "local" to "widespread dissemination," or it is described in the protocol as "sufficient to change treatment methods." Examples include pleural effusion increasing from trace amounts to large amounts, lymphatic involvement spreading from the primary site to distant locations, or may be described in the protocol as "necessitating changes in treatment." If definitive progression is observed, the subject should be considered to have disease progression overall at that time point. It is preferable to have objective criteria applicable to the evaluation of non-measurable lesions, and it is important to note that the criteria for progression must be reliable.

4.3.5 New Lesions

The appearance of new malignant lesions indicates disease progression; therefore, evaluation of these new lesions is crucial. Currently, there are no specific criteria for imaging detection of lesions, but the discovery of a new lesion should be unequivocal. For instance, progression cannot be attributed to differences in imaging techniques, changes in imaging morphology, or lesions other than tumors (e.g., some so-called new bone lesions may merely be healing or recurrence of the original lesion). This is particularly important when the patient's baseline lesions show partial or complete response, for example, necrosis of a liver lesion may be misinterpreted as a new cystic lesion on a CT report, which is not actually the case.

Lesions detected during follow-up but not found in baseline examinations will be considered new lesions and indicate disease progression. For example, a subject with visceral lesions detected at baseline who develops intracranial metastases during a CT or MRI scan of the head will be considered to have disease progression based on the intracranial metastases, even if a head scan was not performed during baseline examination.

If a new lesion is unclear, such as due to its small size, further treatment and follow-up evaluation are needed to confirm whether it is a new lesion. If repeated examinations confirm that it is a new lesion, then the time of disease progression should be calculated from the initial time of detection.

FDG-PET evaluation of lesions generally requires additional testing for confirmation. It is reasonable to combine FDG-PET findings with supplementary CT results to assess progression (especially for new suspicious lesions). New lesions can be definitively identified through FDG-PET, following these procedures:

If the baseline FDG-PET result is negative, and the subsequent FDG-PET result is positive, it indicates disease progression.

If a baseline FDG-PET examination is not performed, and the subsequent FDG-PET result is positive: if the new lesions found on the FDG-PET scan are consistent with the CT scan results, it confirms disease progression.

If the positive FDG-PET result identifies new lesions that are not confirmed by CT, further CT examination is needed for confirmation (if confirmed, the time of disease progression is calculated from the initial abnormal FDG-PET result).

If the positive FDG-PET result is consistent with existing lesions on CT, and there is no progression in imaging, there is no disease progression.

4.4 Overall Best Response Evaluation

The overall best response evaluation refers to the record of the best therapeutic effect from the beginning to the end of the trial, taking into account any necessary conditions for confirmation. Sometimes, the therapeutic response occurs after the end of treatment, so the protocol should specify whether the evaluation of the therapeutic effect after the end of treatment is considered in the overall best response evaluation. The protocol must clarify how any new treatment prior to progression affects the best response. The subject's best response mainly depends on the outcomes of target lesions and non-target lesions, as well as the performance of new lesions. Additionally, it also relies on the nature of the trial, protocol requirements, and result measurement standards. Specifically, in non-randomized trials, the therapeutic response is the primary objective, and the confirmation of PR or CR is essential to determine which is the overall best response.

4.4.1 Time-Point Response

Assuming that therapeutic responses occur at specific time points in each protocol. Table 1 will provide a summary of the overall therapeutic response at each time point for subjects with measurable disease at baseline, including or excluding non-target lesions.

Table 1: Time-Point Response: Subjects with Target Lesions (Including or Excluding Non-Target Lesions)

| Target Lesions | Non-Target Lesions | New Lesions | Overall Response |
| --- | --- | --- | --- |
| CR | CR | Non | CR |
| CR | Non- CR/ Non- PD | Non | PR |
| CR | Not Evaluable | Non | PR |
| PR | Non-Progression or Not Fully Evaluable | Non | PR |
| SD | Non-Progression or Not Fully Evaluable | Non | SD |
| Not Fully Evaluable | Non-Progression | Non | NE |
| PD | Any Condition | Yes or No | PD |
| Any Condition | PD | Yes or No | PD |
| Any Condition | Any Condition | Yes | PD |
| CR = Complete Response, PR = Partial Response, SD = Stable Disease, PD = Progressive Disease,  NE = Not Evaluable | | | |

If the subject has no measurable lesions (no target lesions), the evaluation can be referred to Table 2.

Table 2: Time-Point Response - Subjects with Only Non-Target Lesions

| Non-Target Lesions | New Lesions | Overall Response |
| --- | --- | --- |
| CR | Non | CR |
| Non- CR or Non- PD | Non | Non- CR or Non- PDa |
| Not Fully Evaluable | Non | NE |
| Not definitive PD | Yes or No | PD |
| Any Condition | Yes | PD |
| a：For non-target lesions, "non-CR/non-PD" refers to a response better than SD (Stable Disease). Since SD is increasingly used as an endpoint indicator for evaluating the efficacy of treatment, the efficacy of non-CR/non-PD is established to address situations where no measurable lesions are specified. | | |

4.4.2 Explanation of Missing and Non-Evaluable Assessments

If lesion imaging or measurement is not possible at a specific time point, the subject will be considered non-evaluable at that time point. If only a partial assessment of lesions is possible during an evaluation, this is generally considered non-evaluable at that time point, unless there is evidence that the missing lesions would not affect the evaluation of the therapeutic response at the specified time point. This scenario is likely to occur in cases of disease progression. For example, if a subject has three lesions with a total of 50mm at baseline, but only two lesions are evaluable later, with a total of 80mm, the subject will be evaluated as having disease progression, regardless of the impact of the missing lesion.

4.4.3 Best Overall Response: Across All Time Points

Once all data for a subject is available, their best overall response can be determined. When the study does not require confirmation of complete or partial responses, the evaluation of the best overall response is: The best response across all time points during the trial (e.g., a subject has SD at the first cycle, PR at the second cycle, and PD at the last cycle, but their best overall response is PR. When the best overall response is SD, it must meet the minimum duration from baseline specified in the protocol. If the minimum duration criterion is not met, even if the best overall response is SD, it is not recognized, and the subject's best overall response will depend on subsequent evaluations. For example, if a subject has SD at the first cycle and PD at the second cycle, but has not met the minimum duration requirement for SD, their best overall response will be PD. Similarly, if a subject is lost to follow-up after being evaluated as SD in the first cycle, they will be considered non-evaluable.

When the study requires confirmation of complete or partial responses, the evaluation of the best overall response is: A complete or partial response can only be declared if each subject meets the partial or complete response criteria specified in the protocol and has a subsequent confirmatory assessment at a specified time point (usually four weeks later) as specified in the protocol. In this case, the best overall response is explained in Table 3.

Table 3: Best Overall Response with Confirmation Required for CR and PR Responses

| First Time Point Overall Response | Subsequent Time Point Overall Response | Best Overall Response |
| --- | --- | --- |
| CR | CR | CR |
| CR | PR | SD，PD or PRa |
| CR | SD | If SD lasts for a sufficient duration, otherwise PD |
| CR | PD | If SD lasts for a sufficient duration, otherwise PD |
| CR | NE | If SD lasts for a sufficient duration, otherwise NE |
| PR | CR | PR |
| PR | PR | PR |
| PR | SD | SD |
| PR | PD | If SD lasts for a sufficient duration, otherwise PD |
| PR | NE | If SD lasts for a sufficient duration, otherwise NE |
| NE | NE | NE |
| CR = Complete Response, PR = Partial Response, SD = Stable Disease, PD = Progressive Disease, NE = Not Evaluable.  a: If CR truly occurred at the first time point and any disease recurs at subsequent time points, even if the subject meets the PR criteria compared to baseline, their response will still be evaluated as PD at later time points (as disease recurrence after CR would indicate progression). The best response depends on whether SD occurs within the shortest treatment interval. However, sometimes an initial evaluation of CR is made, but subsequent scans suggest that small lesions still seem to be present, indicating that the subject's response should have been PR instead of CR at the first time point. In such cases, the initial CR judgment should be revised to PR, and the best response would be PR. | | |

4.4.4 Special Considerations for Response Evaluation

When nodular lesions are included in the overall target lesion assessment and the size of these nodules shrinks to "normal" size (<10 mm), they will still be reported in a lesion size scan. To avoid overestimating response based on increases in nodule size, even if the nodules are normal, the measurement results will still be recorded. As previously mentioned, this means that subjects with complete response (CR) will not be recorded as 0 on the CRF (Case Report Form).

If response confirmation is required during the trial, repeated "non-measurable" time points can complicate the assessment of best response. The analysis plan of the trial must specify how these missing data/evaluations can be interpreted clearly. For example, in most trials, a subject's response pattern of PR-NE-PR can be considered as confirmed response.

When a subject's overall health condition deteriorates to the point where drug administration needs to be stopped, but there is no objective evidence to support this, it should be reported as symptomatic progression. Even after treatment termination, efforts should be made to assess objective progression. Symptomatic deterioration is not a descriptor of objective response; it is a reason for stopping treatment. The objective response of such subjects will be assessed based on the target and non-target lesions as shown in Tables 1 to 3.

Cases defined as early progression, early death, and not evaluable are specific research scenarios and should be explicitly described in each protocol (depending on the treatment interval and treatment cycle).

In some cases, it may be difficult to distinguish local lesions from normal tissue. When the assessment of complete response relies on such definitions, a biopsy is recommended before evaluating the response of local lesions to complete response. When abnormal imaging findings of local lesions in some subjects are considered to represent lesion fibrosis or scar formation, FDG-PET is used as a similar evaluation criterion to biopsy for confirming the response of complete response. In such cases, the use of FDG-PET should be prospectively described in the protocol, with support from specialized medical literature reports for this scenario. However, it must be recognized that due to the limitations of FDG-PET and biopsy itself (including their resolution and sensitivity), false-positive results may occur in the assessment of complete response. For unclear progression findings (such as very small uncertain new lesions; cystic or necrotic lesions of the original lesion), treatment can continue until the next assessment. If disease progression is confirmed in the next assessment, the date of progression should be the date when suspected progression first appeared.

4.5 Frequency of Tumor Re-evaluation

The frequency of tumor re-evaluation during treatment depends on the treatment protocol and should be aligned with the type and schedule of treatment. However, in Phase II trials where the therapeutic benefit is unclear, it is reasonable to conduct follow-up every 6-8 weeks (timed to coincide with the end of a treatment cycle), with adjustments to the interval length in specific protocols or situations. The protocol should specify which tissue sites need to be evaluated at baseline (usually those most likely to be associated with metastases of the tumor type being studied) and the frequency of repeated evaluations. Normally, target lesions and non-target lesions should be evaluated during each assessment. In some optional situations, the evaluation frequency of certain non-target lesions can be reduced, for example, bone scans may only need to be repeated when the response of the target disease is confirmed as CR or when there is suspicion of bone lesion progression. After treatment ends, the re-evaluation of tumors depends on whether the response rate or the time to a certain event (progression/death) is used as the clinical trial endpoint. If it is the time to a certain event (e.g., TTP/DFS/PFS), then regular repeated evaluations specified in the protocol are required. Especially in randomized comparative trials, pre-scheduled evaluations should be listed in the timeline (e.g., 6-8 weeks during treatment, or 3-4 months after treatment), and should not be influenced by other factors such as treatment delays, dosing intervals, and any other events that may lead to imbalance in treatment arms in the timing of disease evaluation.

4.6 Evaluation of Therapeutic Effect/Confirmation of Remission Period

4.6.1 Confirmation

For non-randomized clinical studies with therapeutic effect as the primary endpoint, the therapeutic effect of PR and CR must be confirmed to ensure that the therapeutic effect is not a result of evaluation errors. This also allows for reasonable interpretation of the results in the context of historical data, provided that the therapeutic effect in these historical data has also been confirmed. However, in all other cases, such as randomized trials (Phase II or III) or studies with disease stability or disease progression as the primary endpoint, therapeutic effect confirmation is no longer required as it has no value in interpreting the trial results. However, the cancellation of the requirement for therapeutic effect confirmation makes the central review for preventing bias even more crucial, especially in open-label trials.

In the case of SD, within the shortest time interval after the start of the trial (generally not less than 6-8 weeks), there should be at least one measurement that meets the SD criteria specified in the protocol.

4.6.2 Overall Remission Period

The overall remission period is the time from when the first measurement meets the criteria for CR or PR (whichever is measured first) to the first actual record of disease recurrence or progression, using the smallest measurement value recorded in the trial as a reference for disease progression. The overall complete remission time is the time from when the first measurement meets the criteria for CR to the first actual record of disease recurrence or progression.

4.6.3 Disease Stabilization Period

The disease stabilization period is the time from the start of treatment to disease progression (in randomized trials, from the time of randomization). The smallest sum in the trial is used as a reference (if the baseline sum is the smallest, it is used as a reference for PD calculation). The clinical relevance of the disease stabilization period varies depending on different studies and diseases. If, in a specific trial, the proportion of patients maintaining the shortest stabilization period is used as the endpoint, the protocol should specify the shortest time interval between two measurements in the definition of SD.

Note: The remission period, stabilization period, and PFS are influenced by the frequency of follow-up after baseline evaluation. The definition of standard follow-up frequency is not within the scope of this guideline. The frequency of follow-up should consider many factors, such as disease type and stage, treatment cycle, and standard specifications. However, if comparisons between trials are needed, the limitations of the accuracy of these endpoints should be taken into account.

4.7 PFS/TTP

4.7.1 Phase II Clinical Trials

This guideline primarily focuses on the application of objective remission as an endpoint in Phase II clinical trials. In some cases, the response rate may not be the optimal choice for evaluating the potential anticancer activity of a new drug/regimen. In these situations, PFS/PPF at a defined time point may be considered as a suitable alternative indicator to provide an initial signal of the biological activity of the new drug. However, it is evident that in a non-controlled trial, these evaluations may be questioned as seemingly valuable observations may be associated with biological factors such as patient selection rather than the effect of drug intervention. Therefore, Phase II clinical trials using these as endpoints are best designed with randomized controls. However, for some tumors with consistent clinical manifestations (often with consistently poor conditions), non-randomized trials may also be justified. In these cases, due to the lack of positive controls, it is important to carefully document evidence of efficacy when assessing the expected PFS or PPF.

### **Attachment IV: Nomogram for Measuring Body Surface Area**

#### Nomogram for Measuring Body Surface Area


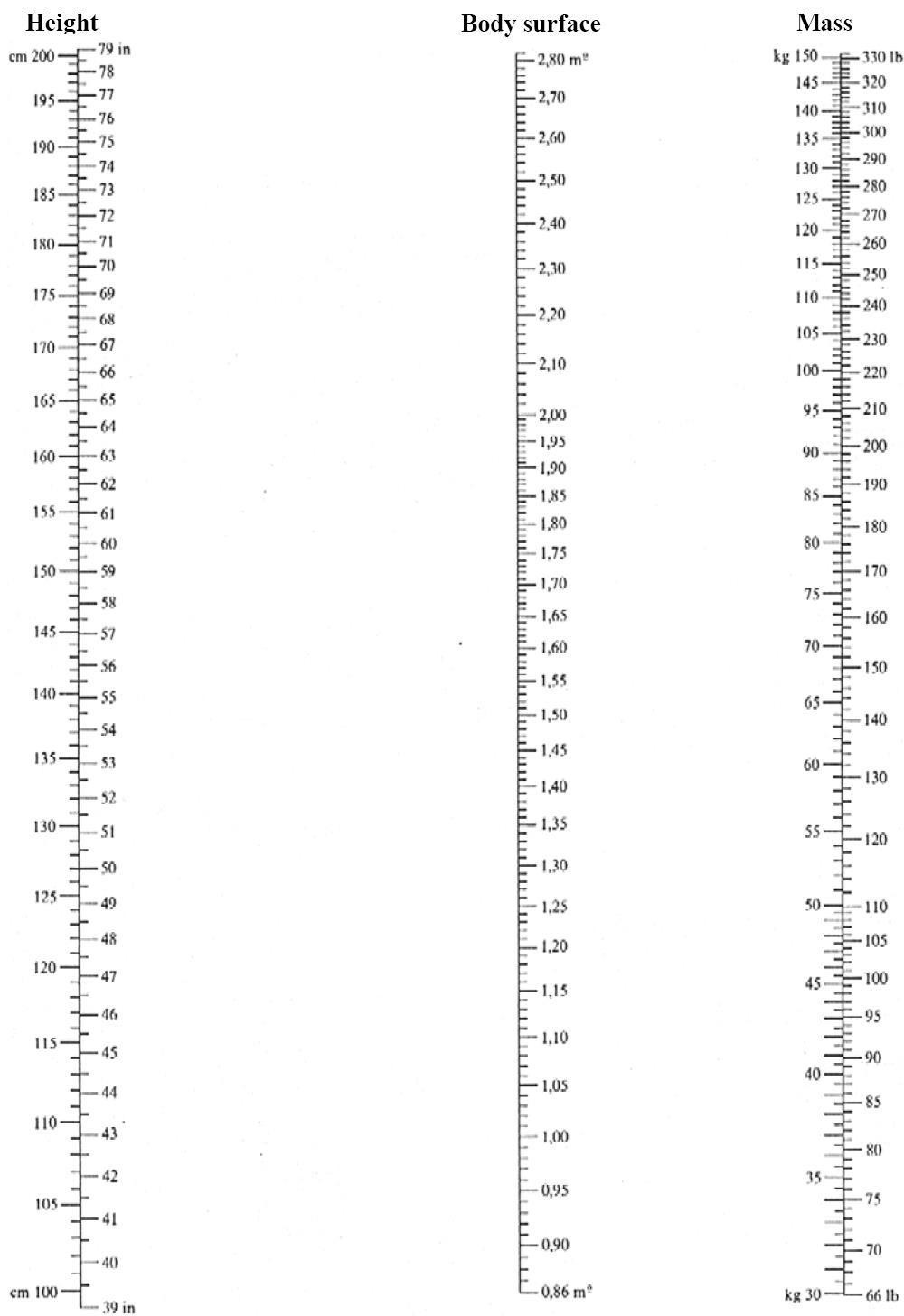


From Du Bois 和Du Bois 的公式，Arch. Intern. Med., 17, 863 (1916)：*0* = *M^0,425^* x*L^0,725^*+x71,84 resp. log*0* =log*M*x0,425+log*L*x0,725+1,8564 (0: "BSA [cm^2^]，M；Weight[kg]：L：Height[cm]

**Attachment V: Standard Guidelines for Surgical Sampling after Neoadjuvant Therapy**

**Standard Guidelines for Surgical Sampling after Neoadjuvant Therapy**

**Locating the Lesion (Tumor or Tumor Bed)**

**Sample Classification**

-Pre-designated location samples refer to those obtained from lesions that have been conspicuously marked using skin tattoos or metal clips before neoadjuvant therapy.

-Samples without pre-designated locations refer to those obtained from lesions that were intended to be pre-marked but were not qualified, or samples without any positioning markers. For instance:

a) Pre-designated but unqualifiedly positioned samples refer to those obtained from lesions that were not clearly marked before neoadjuvant therapy but were located during surgery.

b) Samples without any positioning markers refer to those obtained from lesions that were neither marked before neoadjuvant therapy nor during surgery.

The following table will provide a reference for pathologists to determine the location of the lesion.

|  | Modified Radical Surgery | Breast-Preserving Surgery |
| --- | --- | --- |
| Pre-positioning  • Skin tattoos  • Metal clips or other markers | Marking corresponding areas | Identification based on surgical markers |
| Non-pre-positioned  • Positioning only before surgery  • Without any positioning markers | Identify quadrants based on distinct anatomical markers on the sample;  Locate the quadrant where the clinically diagnosed mass was recorded;  Quadrant identification | Identification based on surgical markers |

Surgical Markers

-When performing breast-preserving surgery

-Method: Use markers at two different spots (as shown in the example figure below; positioning is determined by clinicians and pathologists at each center based on the specific situation and case-by-case analysis principles)

Figure 1: Example of Surgical Markers


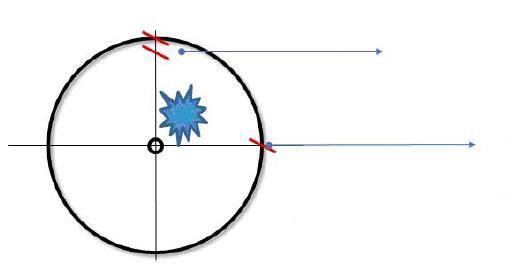


Determining Lesion Size (Tumor or Tumor Bed) Carefully identify the lesion and determine the size of the tumor or tumor bed using three different diameters:

-Pre-designated location samples: Carefully identify the lesion under the marker and determine the size of the tumor or tumor bed using three diameters.

-Pre-designated but unqualifiedly positioned samples: Carefully identify the lesion in the corresponding quadrant (quadrant positioning refers to the "surgical markers" mentioned above, same below) and measure the size of the tumor or tumor bed using three different diameters.

-Samples without any positioning markers: Carefully identify the quadrant where the lesion is located and determine the size of the tumor or tumor bed using three diameters.

Excision Tumor Sample

**Breast Tissue**

-Pre-designated location samples: Samples obtained from breast tissue corresponding to the pre-designated marker (such as a skin tattoo). Make a cut every 1 cm along the long axis of the breast tissue and divide the sample into several tissue blocks. Obtain at least one sample from each tumor block. For example, obtain at least 5 samples from a 5 cm large tissue.

-Pre-designated but unqualifiedly positioned samples and samples without any positioning markers: Samples should be carefully obtained from the quadrant corresponding to the primary tumor mass.

1. Samples with significant tumor mass: Make a cut every 1 cm along the long axis of the sample and divide it into several tissue blocks. Obtain at least one sample from each tumor block. For example, obtain at least 5 samples from a 5 cm large tissue.

a) If there is microscopic non-pathological complete response (pCR), no additional sampling is necessary.

b) If there is microscopic pCR, obtain samples from all quadrants (if it is breast-preserving surgery, obtain samples from the entire breast tissue).

2. Samples with insignificant tumor mass but fibrotic tumor bed: Use the same method to obtain samples from the fibrotic tumor bed.

a) If there is microscopic non-pCR, no additional sampling is necessary.

b) If there is microscopic pCR, obtain samples from all quadrants (if it is breast-preserving surgery, obtain samples from the entire breast tissue).

3. Samples without significant tumor mass or fibrotic tumor bed: Obtain samples from all areas under the marker (if it is breast-preserving surgery, obtain samples from the entire breast tissue).

**Lymph Nodes**

-Thoroughly examine the lymph nodes in axillary adipose tissue.

-Slice the lymph nodes along their long axis.

-Submit the largest section for histological evaluation of adjacent connective tissue. All identified axillary lymph nodes should be submitted for histological examination. For more detailed information on sentinel lymph node biopsy, please refer to respective guideline documents. This sampling guideline only provides standard post-surgical sampling methods after neoadjuvant therapy.

#### Attachment 6: Flowchart for Breast Tissue Sampling

#### Flowchart of Breast Tissue Sampling


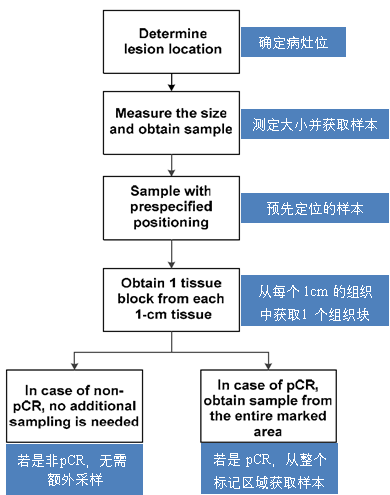

Supplement: Supplementary file 2 — Protocol of the Neopather trial [file 41392_2025_2138_MOESM2_ESM.doc]
